# Sexual Health After Neurological Disorders: A Comprehensive Umbrella Review of Treatment Evidence

**DOI:** 10.3390/medsci14010037

**Published:** 2026-01-10

**Authors:** Alfredo Manuli, Andrea Calderone, Desiree Latella, Fabrizio Quattrini, Gianluca Pucciarelli, Rocco Salvatore Calabrò

**Affiliations:** 1 Department of Biomedicine and Prevention, University of Rome Tor Vergata, 00133 Rome, Italy; alfredo.manuli@irccsme.it (A.M.); gianluca.pucciarelli@uniroma2.it (G.P.); 2 IRCCS Centro Neurolesi Bonino-Pulejo, S.S. 113 Via Palermo, C.da Casazza, 98124 Messina, Italy; desiree.latella@irccsme.it (D.L.); roccos.calabro@irccsme.it (R.S.C.); 3 Università degli Studi Niccolò Cusano—Telematica Roma (UniCusano), 00166 Rome, Italy; fabrizio.quattrini@unicusano.it

**Keywords:** neurological disorders, sexual dysfunction, sexual health, neurorehabilitation, umbrella review, Parkinson’s disease, spinal cord injury, multiple sclerosis, epilepsy, quality of life

## Abstract

**Background/Objectives:** Sexual dysfunction (SD) and broader sexual health problems are common after neurological disorders, yet interventional evidence is fragmented across conditions and outcomes. This umbrella review mapped and appraised systematic review-level evidence on interventions targeting SD and sexual health in neurological populations and qualified conclusions using certainty of evidence. **Methods:** PubMed, Web of Science, Cochrane Library, Embase, PsycINFO, EBSCOhost, and Scopus were searched from inception to 27 November 2025. Two reviewers screened records, extracted data, assessed review quality with AMSTAR 2, and rated certainty across intervention–outcome pairings using a GRADE-informed approach that integrated review confidence and primary-study risk-of-bias as reported by the source reviews. **Results:** Twenty-six systematic reviews were included. Overall confidence was frequently limited (17/26 critically low and 6/26 low), with only a small subset rated moderate or higher. Evidence was most coherent for phosphodiesterase type 5 (PDE5) inhibitors improving erectile function in men with spinal cord injury, whereas most other interventions and outcomes were supported by low or very low certainty. Women were represented in 16/26 reviews, yet validated female sexual function outcomes were synthesized in 6/26 reviews and relationship/couple outcomes in 3/26; furthermore, 10/26 reviews restricted inclusion to men, and no review synthesized pediatric intervention trials. **Conclusions:** Evidence supports PDE5 inhibitors for improving erectile function in men with spinal cord injury, while evidence for other interventions and sexual health domains remains limited. Methodological limitations highlight the need for more inclusive trials, broader standardized outcomes, and longer follow-up within neurorehabilitation pathways.

## 1. Introduction

Neurological disorders are a heterogeneous group of diseases affecting the central, peripheral, and autonomic nervous systems, including the brain, spinal cord, and cranial and peripheral nerves [1,2]. They encompass acquired conditions (e.g., stroke, traumatic brain injury (TBI), spinal cord injury (SCI), epilepsy, headache) and neurodegenerative disorders (e.g., multiple sclerosis (MS), Parkinson’s disease (PD), dementias, neuromuscular disorders), which can disrupt motor, sensory, cognitive, and autonomic functions and thereby limit independence and quality of life [1,2]. Recent analyses from the Global Burden of Disease 2021 study indicate that disorders affecting the nervous system now represent the leading cause of disability-adjusted life years (DALYs) worldwide [1]. In 2021, an estimated 3.4 billion individuals (43.1% of the global population) were living with at least one neurological condition, accounting for about 11.1 million deaths and 443 million DALYs, with disproportionate impact in low- and middle-income countries [1].

Within this context, sexual health is an integral dimension of human well-being [3,4]. The World Health Organization (WHO) defines sexual health as “a state of physical, emotional, mental and social well-being in relation to sexuality, and it is not merely the absence of disease, dysfunction or infirmity, “requiring” a positive and respectful approach to sexuality and sexual relationships, as well as the possibility of having pleasurable and safe sexual experiences, free of coercion, discrimination and violence” [5,6,7]. Sexual dysfunction (SD) is highly prevalent in neurological populations and often exceeds rates in the general population [8]. At least half of stroke survivors report difficulties with desire, arousal, and satisfaction [9], and SD affects around 60% of women and 64–91% of men with MS [10] and approximately 40–90% of individuals with PD [11]. In epilepsy, sexual problems occur 2–4 times more frequently than in control populations, with prevalence estimates of 18–79% [12], and in SCI, SD is almost universal and may reach 80–90% [13]. To standardize terminology across reviews and outcomes, we use SD to indicate persistent or recurrent difficulties in desire, arousal, orgasm, or sexual pain that are clinically meaningful because they generate distress and/or interpersonal difficulty. Sexual health and sexual well-being are used more broadly to include satisfaction, intimacy, participation, and relationship functioning, while sexual quality of life is reserved for outcomes explicitly framed as quality-of-life constructs. Where evidence is limited to erectile endpoints, we refer specifically to erectile function to avoid overgeneralizing findings.

Neurological conditions can affect sexuality through interacting neurophysiological, symptom-related, and psychosocial pathways [14,15]. Lesions in networks supporting desire and genital response may directly impair sexual function, while fatigue, spasticity, pain, sensory changes, and bladder or bowel dysfunction can reduce feasibility and comfort of sexual activity [14]. Cognitive and emotional sequelae, including mood and anxiety disorders and post-traumatic symptoms, may further contribute to sexual distress and avoidance of intimacy [16,17]. These processes can undermine body image and sexual identity and place additional strain on couple relationships as partners adapt to disability and changed roles [18,19]. Although sexual health needs extend across the lifespan, interventional evidence is often sparse in childhood-onset conditions, and available work more commonly documents needs and barriers than supported treatment effects [20,21,22,23,24,25].

Despite the high burden, care pathways remain fragmented, and sexual concerns are frequently unaddressed because of stigma, limited training, and time constraints in neurology and rehabilitation settings [26,27,28,29,30]. When SD is discussed, management may focus narrowly on physiological symptoms (e.g., erectile function) without systematically addressing distress, relationship functioning, or contextual determinants of sexual well-being [28,30]. In parallel, sexual rehabilitation has increasingly been framed within multidisciplinary, person-centered neurorehabilitation models consistent with international perspectives that position sexual health as participation and quality of life, including partner involvement where appropriate [31,32,33,34]. However, the interventional evidence base remains heterogeneous and uneven, with many small, short-term, condition-specific studies and non-standardized outcomes; pharmacological trials in men with SCI or MS often emphasize erectile endpoints, while psychosocial and couple-focused interventions frequently show promising but limited evidence [35,36]. In several populations, particularly pediatric CP, childhood-onset epilepsy, and rare neuromuscular disorders, rigorous intervention research remains sparse [37,38].

An umbrella review is therefore well suited to this field because it can synthesize systematic review-level evidence, map where intervention data are concentrated versus absent, and qualify certainty within condition-specific intervention–outcome pairings rather than ranking treatments across heterogeneous disorders. Accordingly, the target of the present work is evidence mapping and certainty-informed interpretation to distinguish clinically actionable signals from hypothesis-generating findings.

The objective of this umbrella review is to systematically identify, appraise, and synthesize systematic reviews of pharmacological, psychological, rehabilitation-based, and multidisciplinary interventions targeting SD or impaired sexual health in neurological populations across the lifespan, summarizing effectiveness and safety or tolerability on sexual function, sexual satisfaction, broader sexual well-being, and, where reported, partner- and couple-level outcomes. We assessed review quality using AMSTAR 2 and qualified conclusions using a GRADE-informed certainty assessment applied to each intervention–outcome pairing, explicitly reflecting review-level confidence, primary-study limitations reported by the source reviews, and the consistency and directness of available effects.

## 2. Materials and Methods

This umbrella review addressed which pharmacological, psychological, rehabilitation-based, and multidisciplinary interventions improve sexual health outcomes in people with neurological disorders across the lifespan. The research question and eligibility criteria were structured using the PICO (population, intervention, comparator, outcome) framework applied to the primary studies contained within each included systematic review [39]. For terminology, we used SD to denote clinically meaningful problems in desire, arousal, orgasm, or sexual pain associated with distress and/or interpersonal difficulty, whereas sexual health/sexual well-being were used to include broader constructs such as satisfaction, intimacy, participation, and relational functioning; sexual quality of life was reserved for outcomes explicitly framed as quality-of-life constructs. These terms were applied both to eligibility (at least one eligible sexual outcome required) and to synthesis (domain-based interpretation when possible).

### 2.1. Research Question and PICO Framework

The population comprised individuals with a diagnosed neurological disorder associated with SD or impaired sexual health, including adults (≥18 years) and pediatric/adolescent participants (<18 years). Eligible neurological conditions included stroke, ABI of traumatic or non-traumatic origin, SCI, MS, PD, epilepsy, cerebral palsy (CP), neuromuscular disorders, and other central or peripheral nervous system diseases where SD represented a direct or frequent consequence of the condition. To increase transparency on coverage, we prespecified childhood-onset conditions (e.g., CP) and neuromuscular disorders as eligible, but we anticipated that eligible systematic intervention reviews would be uneven across conditions and age groups; in practice, several pediatric populations (including pediatric CP) and a number of specific neuromuscular disorders lacked eligible systematic intervention reviews and were therefore treated as evidence gaps rather than as areas with synthesizable intervention effects.

Systematic reviews enrolling mixed clinical populations were eligible only when neurological data were extractable and reported separately or when at least 70% of the included participants had a neurological disorder. To operationalize the ≥70% rule in mixed populations, we used the proportion reported by the review authors at the level of included primary studies or pooled samples; for example, a review combining neurological and non-neurological etiologies of SD was eligible when ≥70% of participants were drawn from neurological diagnoses (e.g., SCI/MS/stroke) even if the remaining minority reflected other etiologies, whereas reviews with a lower neurological proportion were included only if neurological subgroup effects were presented separately.

Eligible interventions were any treatments primarily intended to prevent or manage SD or to improve sexual health in the context of neurological disorders. Pharmacological interventions included phosphodiesterase type 5 (PDE5) inhibitors, dopaminergic drugs, hormonal therapies, and other centrally or peripherally acting medications. Psychological and behavioral interventions included individual or couples-based sex therapy, cognitive behavioral interventions, psychoeducation, counseling, and relationship-focused programs. Rehabilitation and multidisciplinary interventions included pelvic floor and genital-focused physiotherapy, neuromodulation or neuromuscular stimulation, structured sexual rehabilitation within neurorehabilitation services, and broader pathways with a clearly defined sexual health component. Comparators included placebo, sham/attention control, usual care, no treatment, waiting list, or active alternatives. Pre–post designs without explicit controls were considered only when review authors treated them as part of a coherent intervention evidence base.

Outcomes of interest were patient-level measures of sexual function and sexual health. Primary outcomes included validated instruments such as the International Index of Erectile Function, the Female Sexual Function Index, the Multiple Sclerosis Intimacy and Sexuality Questionnaire, and comparable condition-specific or generic measures of sexual function and sexual satisfaction. Secondary outcomes included broader sexual well-being, relationship and partner outcomes, sexuality-related distress, and health-related quality of life with a sexual health component. Safety outcomes included adverse events, discontinuation due to adverse effects, and serious harms. Systematic reviews were eligible when at least one outcome mapped onto sexual function, sexual satisfaction, sexual well-being, or sexual quality of life at the patient level.

### 2.2. Inclusion Criteria

This umbrella review included published systematic reviews with or without meta-analysis that synthesized interventional studies on treatments for SD or impaired sexual health in people with neurological disorders. Reviews were required to report a clear research question framed around a neurological population with SD and at least one defined intervention. A systematic search of at least one electronic database and a reproducible search strategy were mandatory features. Reviews also needed to describe a study selection process that involved screening of titles and abstracts and full texts and to provide quantitative or qualitative synthesis of treatment effects. Eligible reviews considered adults, pediatric patients, or mixed-age populations. Reviews focusing on conditions with onset in childhood, such as cerebral palsy, were explicitly eligible when outcomes related to sexual function, sexual health, or intimate relationships in adolescence or adulthood were reported. Reviews could focus on a single neurological condition or pool across different neurological populations, provided that sexual health outcomes were clearly identified. For reviews including mixed clinical populations, eligibility required that at least 70% of the included participants had a neurological diagnosis or that neurological subgroup data were extractable and reported separately. This rule was applied to preserve clinical interpretability while avoiding indirectness driven by non-neurological etiologies. Randomized controlled trials, quasi-experimental designs, and observational intervention studies at the primary study level were all acceptable within the included reviews. Reviews that addressed pharmacological, psychological, rehabilitation-based, or multidisciplinary interventions, or any combination of these, were eligible. Only reviews published in peer-reviewed journals and written in English were considered. This restriction was applied to ensure feasibility of duplicate screening and consistent appraisal across reviewers. However, we acknowledge that it may have excluded relevant evidence published in other languages and therefore may contribute to selection and publication-related biases.

### 2.3. Exclusion Criteria and Management of Overlapping Reviews

We excluded narrative reviews, expert opinions, editorials, commentaries, guidelines, and scoping reviews without systematic methods. Reviews focused exclusively on non-neurological causes of SD were excluded unless neurological subgroup data met the PICO-aligned eligibility requirements. Reviews addressing sexual education or sexual risk behavior without assessing sexual function, sexual satisfaction, or sexual quality of life were excluded, as were reviews reporting sexual outcomes only as incidental adverse events of treatments targeting the neurological condition. Reviews limited to prevalence/correlates without intervention evaluation and non-peer-reviewed reports (including conference abstracts without full publication) were excluded.

Overlapping systematic reviews were handled using a structured clustering approach. We formed “overlap clusters” defined by the same neurological population (or closely comparable populations), the same intervention category (e.g., PDE5 inhibitors), and the same primary outcome domain (e.g., erectile function). Within each cluster, we identified a single primary review to anchor effect interpretation and to minimize double-counting of underlying primary trials. The primary review was selected using a pre-specified hierarchy: Cochrane reviews were prioritized when available; otherwise, we selected the review with the highest AMSTAR 2 overall confidence and/or the most comprehensive and up-to-date coverage of the intervention–outcome pairing. To indicate how this was applied in major domains, PDE5 inhibitors in SCI were anchored to the network meta-analytic synthesis, pharmacological treatment of erectile dysfunction in MS was anchored to the Cochrane review, and stroke/ABI evidence was anchored to the Cochrane review; overlapping reviews within these clusters were used to capture complementary outcomes or subgroup information and to assess stability of conclusions rather than to contribute additional, duplicative effect estimates.

Because trial identifiers were inconsistently reported across reviews, we did not calculate a formal overlap metric (e.g., corrected covered area). Instead, overlap was treated explicitly as a potential source of indirectness and double-counting; this was addressed by extracting pooled estimates only once per overlap cluster (from the primary review) and reflecting residual overlap uncertainty conservatively in certainty judgements.

### 2.4. Search Strategy

The search strategy was designed to maximize sensitivity for systematic reviews of treatments for SD in neurological populations while preserving specificity for the sexual health focus and for review-level evidence. Searches covered the period from database inception to 27 November 2025 with no a priori date restrictions. This comprehensive and highly sensitive strategy was justified by the expectation that relevant evidence on sexual health after neurological disorders would be dispersed across multiple clinical specialties (neurology, rehabilitation, sexual medicine, psychology) and published over several decades using heterogeneous terminology. A broad, multi-database search without a priori date restrictions was therefore necessary to minimise the risk of missing influential but older or discipline-specific reviews, to reduce selection bias, and to allow a robust mapping of the full review-level evidence base on treatments for SD in neurological populations. The following databases were searched using controlled vocabulary and free text terms adapted to each platform. The databases were PubMed, Web of Science, the Cochrane Library, Embase, EBSCOhost, PsychINFO, and Scopus. A validated or explicit filter for systematic reviews and meta-analyses was applied where possible.

Core concepts covered three domains. The first domain captured neurological disorders and neurorehabilitation populations. The second domain captured SD and sexual health outcomes, including SD, erectile dysfunction, female SD, libido, sexual desire, sexual satisfaction, and sexual quality of life. The third domain captured treatments and review design aspects and included terms for therapy, pharmacological and non-pharmacological interventions, rehabilitation, and systematic review or meta-analysis. Searches in MEDLINE used MeSH terms and keywords in three complementary core strings that can be adapted to other databases. These strings were developed without truncation or wildcards and are reported with field tags for transparency.

String 1: (“Stroke” OR “Brain Injuries” OR “Spinal Cord Injuries” OR “Multiple Sclerosis” OR “Parkinson Disease” OR “Epilepsy”) AND (“Sexual Dysfunction, Physiological” OR “Sexual Dysfunctions, Psychological” OR “Erectile Dysfunction”) AND (“Rehabilitation” OR “Neurological Rehabilitation” OR “Psychotherapy” OR “Sex Counseling”).

String 2: (“Stroke” OR “Brain Injuries” OR “Spinal Cord Injuries” OR “Multiple Sclerosis” OR “Parkinson Disease” OR “Epilepsy” OR “Cerebral Palsy” OR “Neuromuscular Diseases”) AND (“Sexual Dysfunction, Physiological” OR “Sexual Dysfunctions, Psychological” OR “Erectile Dysfunction” OR “Sexual Partners” OR “Marital Relations” OR “Interpersonal Relations”) AND (“Sex Counseling” OR “Psychotherapy” OR “Cognitive Behavioral Therapy” OR “Behavior Therapy” OR “Neurological Rehabilitation” OR “Physical Therapy Modalities”).

String 3: (“Child” OR “Adolescent” OR “Cerebral Palsy”) AND (“Spinal Cord Injuries” OR “Epilepsy” OR “Brain Injuries” OR “Multiple Sclerosis” OR “Parkinson Disease” OR “Neurological Disorders”) AND (“Sexual Dysfunction, Physiological” OR “Sexual Dysfunctions, Psychological” OR “Sexuality” OR “Sexual Health”) AND (“Psychotherapy” OR “Sex Counseling” OR “Rehabilitation” OR “Sexual Interventions” OR “Pharmacological Treatments”).

**Figure 1:**
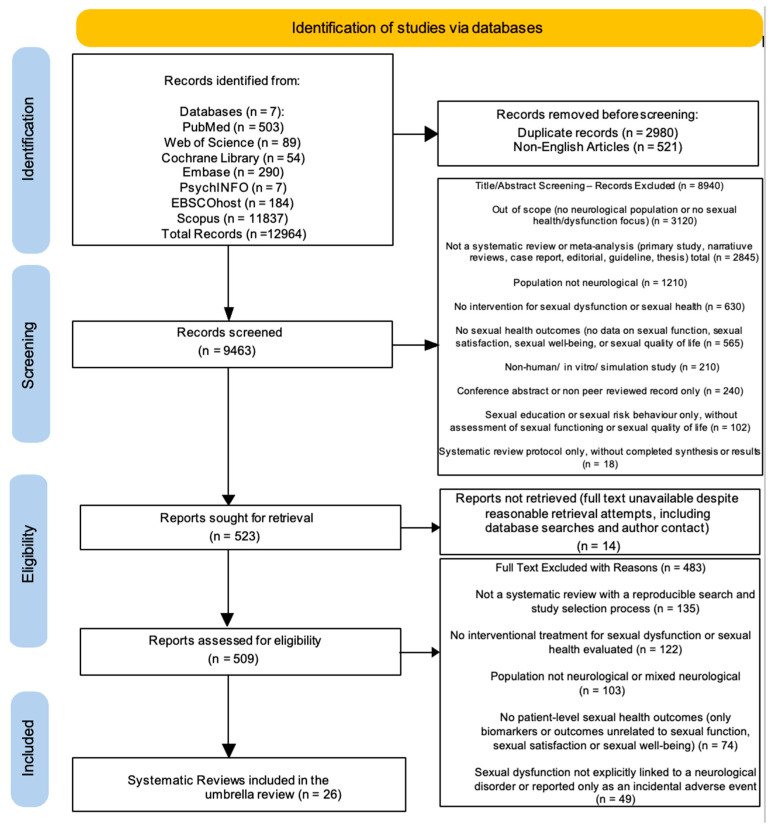
PRISMA 2020 flow diagram of evaluated studies.

These strings were adapted to databases by replacing MeSH terms with the corresponding thesaurus terms or subject headings and by using title, abstract, and keyword fields as appropriate. Reference lists of all included systematic reviews and of key guideline and narrative papers on sexual health in neurological disorders were screened to identify additional eligible reviews. Citation tracking in Web of Science and Scopus was used to detect related and more recent systematic reviews. This systematic review was performed in accordance with the PRISMA (Preferred Reporting Items for Systematic Reviews and Me-ta-Analyses) guidelines as illustrated in Figure 1 [40].

#### 2.4.1. Study Selection, Data Extraction and Management

Two reviewers (AM and AC) independently screened titles and abstracts and then assessed full-text articles against the predefined eligibility criteria using piloted standardized forms. Any disagreements were discussed until a shared decision was reached. Cases that remained unresolved were referred to RSC, who acted as a third reviewer. Inter-rater agreement at both screening stages was quantified using Cohen’s kappa with 95 percent confidence intervals and was interpreted according to conventional thresholds, with values of 0.61 or higher indicating substantial agreement and values above 0.81 indicating almost perfect agreement [41]. AM and AC also carried out data extraction independently using structured templates that were specifically developed and piloted for this umbrella review. Each included systematic review contributed bibliographic details, the journal, the year of publication, and the country of the corresponding author. Methodological features were recorded, including databases searched, date ranges, language restrictions, presence of a registered protocol, eligibility criteria, risk of bias tools, and methods used for qualitative or quantitative synthesis.

Population characteristics were extracted with attention to age groups and developmental stage. Information included diagnostic categories, inclusion and exclusion criteria applied to primary studies, sample sizes, proportions of adult and pediatric participants, sex distribution, age range, disease duration, and setting of care. Interventions were coded by category, such as pharmacological, psychological, rehabilitation-based, or multidisciplinary, and described in relation to content, intensity, duration, delivery format, and context. Comparators were classified as placebo, sham, active control, usual care, or pre–post designs, and details on the number and design of primary studies and total sample sizes were captured. Sexual health outcomes were recorded with emphasis on the instruments used, psychometric properties were reported, direction of scoring, and timing of outcome assessment.

Meta-analytic results were extracted when present, including effect estimates, confidence intervals, heterogeneity measures, statistical models, and results of subgroup or sensitivity analyses relevant to age, sex, or specific neurological diagnoses such as CP or SCI. Safety and tolerability data, including adverse events, serious adverse events, and treatment discontinuation, were extracted for each intervention where available. Funding sources and authors’ conflicts of interest were recorded. All extracted data were cross-checked between AM and AC, and any discrepancies were resolved through consensus. Data were entered into Microsoft Excel workbooks that used locked templates, predefined validation rules, and range checks to limit transcription errors and to maintain a transparent audit trail of all decisions. Built-in filters and pivot table functions supported internal consistency checks and preparation of the dataset for synthesis. The protocol for this umbrella review was registered prospectively in PROSPERO (CRD420251240006), which provided a prespecified methodological framework and strengthened transparency and resistance to selective reporting [42].

#### 2.4.2. Methodological Quality, Risk of Bias Assessment and Certainty of Evidence

Methodological quality of included systematic reviews was assessed using AMSTAR 2 [43] by two independent reviewers (AM and AC) after calibration, with arbitration by a third reviewer if needed. Risk-of-bias assessments of primary studies were extracted as reported in each review without modification. When reported, tools such as RoB 2 for randomized trials [44] and ROBINS-I for non-randomized studies [45] were recorded.

Certainty of evidence was graded using an adaptation of GRADE applied at the umbrella-review level to each intervention–outcome pairing [46]. For each condition-specific pairing, we defined a “body of evidence” by the overlap cluster described above, anchored by a primary review and supplemented by overlapping reviews for triangulation rather than additive pooling. The starting certainty reflected the predominant primary-study design contributing to the cluster (high when evidence was mainly randomized trials; low when mainly non-randomized intervention studies). Downgrading followed standard GRADE domains (risk of bias, inconsistency, indirectness, imprecision, publication bias) but was made explicit for umbrella-level challenges: risk of bias incorporated both the primary-study appraisals and review-level credibility, such that critically low or low AMSTAR 2 confidence increased the likelihood of downgrading when core review safeguards (e.g., protocol transparency, bias assessment, or synthesis methods) were insufficiently reported. Inconsistency was evaluated using direction of effects across reviews and heterogeneity indices when available. Indirectness captured population mixing (including reliance on the ≥70% rule when subgroup effects were unavailable), intervention heterogeneity within categories, outcome-domain mismatch, and—importantly—unquantified overlap, which was treated conservatively as a potential source of double-counting and indirectness rather than ignored. Imprecision was judged based on sample size, confidence interval width, and event counts when reported. Publication bias considered small-study effects and funding signals as reported by the source reviews. Upgrading was considered only when supported by consistent, precise effects across multiple higher-credibility syntheses with clinically coherent estimates. Final certainty ratings (high, moderate, low, and very low) were assigned by consensus and used to qualify the wording of conclusions and clinical implications.

### 2.5. Narrative Synthesis Framework (SWiM)

The evidence synthesis followed a structured narrative approach aligned with guidance for syntheses without meta-analysis (SWiM) and for overviews of reviews [47]. The systematic review was treated as the primary unit of analysis, and the focus of interpretation was the pattern and consistency of treatment effects across reviews for each neurological condition and intervention category.

Studies were first grouped by neurological diagnosis into categories such as SCI, MS, stroke/ABI, PD, epilepsy, and mixed or less-studied neurological conditions, recognizing a priori that evidence relevant to childhood-onset conditions would likely be sparse at the systematic review level. Across categories, outcomes were synthesized by domain (e.g., erectile function, broader sexual function, satisfaction/quality-of-life constructs, relationship/partner outcomes), and we reported the direction of effects as presented by the source reviews without recalculating pooled estimates. Each diagnostic group was then organized by intervention type, including pharmacological, psychological, rehabilitation-based, and multidisciplinary or combined approaches. Each intervention type was summarized according to the direction and magnitude of effects on sexual function and sexual health outcomes, together with information on precision, heterogeneity, and methodological quality as reported in the source reviews.

Meta-analyses within reviews were given descriptive priority, and pooled effect estimates were reported alongside the number of primary studies and participants, without undertaking any de novo quantitative synthesis of primary data. Overlapping reviews were compared in terms of methods, primary study coverage, and conclusions; the most recent and methodologically robust review was prioritized, and areas of agreement or disagreement were explicitly described. Sex-specific and gender-relevant findings were highlighted whenever separate analyses for men and women or for different sexual orientations were available. Particular attention was paid to couples-based interventions and partner-related outcomes, where reported.

Certainty of evidence ratings derived using the GRADE-based approach described above were integrated into the narrative synthesis so that conclusions and clinical implications reflected both the observed effects and the robustness of the underlying evidence. The final synthesis aimed to provide clinically meaningful and mechanism-informed conclusions regarding the capacity of available treatments to improve sexual health outcomes after neurological disorders in both adult and pediatric populations and to identify areas where robust interventional evidence remains limited. A comprehensive overview of all methodological components, including detailed PICO operationalization, full eligibility criteria, complete search strings, data extraction domains, and risk-of-bias procedures, is provided in Table 1.

## 3. Results

We searched seven electronic databases (PubMed, Web of Science, Cochrane Library, Embase, PsychINFO, EBSCOhost, and Scopus) and identified 12,964 records (PubMed n = 503; Web of Science *n* = 89; Cochrane Library *n* = 54; Embase *n* = 290; PsychINFO *n* = 7; EBSCOhost *n* = 184; Scopus *n* = 11,837). After removal of 2980 duplicate records and 521 non-English articles, 9463 records remained for title and abstract screening. At this stage, 8940 records were excluded because they were out of scope (no neurological population or no sexual health/dysfunction focus; *n* = 3120), were not a systematic review or meta-analysis (primary studies, narrative reviews, case reports, editorials, guidelines, theses; *n* = 2845), included a non-neurological population (*n* = 1210), did not evaluate an intervention for SD or sexual health (*n* = 630), reported no sexual health outcomes (no data on sexual function, sexual satisfaction, sexual well-being, or sexual quality of life; *n* = 565), were non-human, in vitro, or simulation studies (*n* = 210), were conference abstracts or other non-peer-reviewed records only (*n* = 240), addressed only sexual education or sexual risk behaviour without assessment of sexual functioning or sexual quality of life (*n* = 102), or were systematic review protocols without completed synthesis or results (*n* = 18). The remaining 523 records were sought for retrieval; 14 full texts could not be obtained despite repeated database checks and contact with corresponding authors, leaving 509 reports for full-text assessment. Of these, 483 were excluded because they were not systematic reviews with a reproducible search and study selection process (*n* = 135), did not evaluate any interventional treatment for SD or sexual health (*n* = 122), involved non-neurological or mixed populations without stratified neurological data (*n* = 103), reported no patient-level sexual health outcomes (only biomarkers or outcomes unrelated to sexual function, sexual satisfaction, or sexual well-being; *n* = 74), or described SD that was not explicitly linked to a neurological disorder or was reported only as an incidental adverse event (*n* = 49). The final sample comprised 26 systematic reviews included in this umbrella review (Figure 1) [48,49,50,51,52,53,54,55,56,57,58,59,60,61,62,63,64,65,66,67,68,69,70,71,72,73].

Neurological conditions covered by the 26 systematic reviews included SCI, MS, PD, stroke and ABI, epilepsy, CP neuromuscular diseases, and mixed central or peripheral neuropathies [48,49,50,51,52,53,54,55,56,57,58,59,60,61,62,63,64,65,66,67,68,69,70,71,72,73] (see Supplementary Table S1). Across conditions, SCI and MS accounted for most included systematic reviews (11/26 and 8/26, respectively), whereas evidence in stroke/ABI (4/26), PD (2/26), and epilepsy (1/26) was comparatively sparse. The large majority of reviews focused on adults, while only a few explicitly considered adolescents or young adults with childhood-onset CP, epilepsy, or neuromuscular disorders [58,59,60,72,73]. Pharmacological interventions, particularly PDE5 inhibitors and other agents for neurogenic erectile dysfunction, represented the most extensively evaluated treatment category, whereas psychological, rehabilitation-based, and multidisciplinary approaches were examined less frequently and were described with greater heterogeneity in terms of content, intensity, and delivery [48,49,50,51,52,54,55,56,57,58,59,64,66,67,68,69,70,71,72,73]. Sex-related coverage was unbalanced: 10/26 reviews restricted inclusion to men, women were represented in 16/26 reviews, validated female sexual function outcomes (most commonly FSFI) were synthesized in 6/26 reviews, and relationship/couple outcomes were synthesized in 3/26 reviews. Half of the reviews reported at least one meta-analysis, mainly for pharmacological or physiotherapy-based interventions, whereas the remainder relied on narrative synthesis of small and methodologically diverse primary studies [50,53,54,55,56,57,58,60,61,63,66,70,71,72]. Sexual outcomes were dominated by male erectile function indices such as the International Index of Erectile Function, and data on female sexual function, global sexual satisfaction, sexual quality of life, and partner or couple outcomes were comparatively sparse [48,49,50,51,52,57,58,59,64,70,73]. Follow-up periods were usually short, so evidence on long-term maintenance of treatment effects, safety, tolerability, and acceptability remained limited [48,49,50,54,55,56,57,58,59,63,64,65,66,70,71,72,73]. The evidence base therefore appeared fragmented and highly condition-specific, with persistent gaps for women, pediatric populations, and complex multidisciplinary interventions [48,49,50,51,52,53,54,55,56,57,58,59,60,69,70,71,72,73]. These features motivated a structured appraisal of review quality with AMSTAR 2, integration of these judgments into GRADE-based certainty ratings, and a SWiM-aligned narrative synthesis organized by neurological condition and intervention category [43,46,47].

### 3.1. Quality of Included Systematic Reviews—Risk of Bias (AMSTAR 2)

The methodological quality of the 26 included systematic reviews was appraised with AMSTAR 2 [43], as prespecified in the Methods. Two reviewers (AM and AC) independently applied the tool and resolved disagreements by consensus, and full item-level ratings are reported in Supplementary Table S2. AMSTAR 2 judgments were used both descriptively and to inform the risk-of-bias domain of the GRADE-based certainty assessments for each intervention–outcome pairing [46].

Overall confidence in the results of the reviews was frequently limited. Seventeen reviews were rated as critically low confidence, including broad overviews of neurogenic SD and condition-specific syntheses in MS, SCI, PD, stroke, and other neurological populations [48,49,51,52,57,59,60,61,62,63,64,65,67,68,69,72,73]. Six reviews were judged low confidence (Jia et al. [54], García-Perdomo et al. [55], Gopal et al. [58], Couper et al. [66], Yavas et al. [70], and Gao et al. [71]). Two Cochrane reviews, one on interventions for SD after stroke (Stratton et al. [50]) and one on sildenafil for erectile dysfunction in MS (Xiao et al. [53]), were rated moderate confidence. One recent PROSPERO-registered network meta-analysis of PDE5 inhibitors in men with SCI-related erectile dysfunction (Tienforti et al. [56]) was judged to have no critical flaws and was rated at the boundary between high and moderate confidence.

Across AMSTAR 2 domains, several strengths were evident. All reviews clearly formulated a PICO-based research question focused on SD or sexual health in neurological populations (Item 1 “Yes” for 26/26). Most implemented a reasonably comprehensive literature search, typically across multiple databases with reproducible strategies (Item 4 “Yes” in 22/26), and reported duplicate study selection and data extraction (Items 5 and 6 “Yes” in 19/26 and 20/26, respectively). Descriptive reporting of included studies was consistently adequate, with all reviews providing at least basic tables of study characteristics (Item 8 “Yes” in 26/26), including condition-specific reviews in MS, SCI, PD, epilepsy and stroke (e.g., Giannopapas et al. [51], DeForge et al. [64], Bahadori et al. [63], Couper et al. [66], Stratton et al. [50]).

However, several critical AMSTAR 2 domains were frequently unmet, driving the predominance of critically low and low overall ratings. Only about one-third of reviews reported a prospectively developed protocol or formal registration (Item 2 “Yes” in 9/26). Protocols were clearly documented in the Cochrane stroke and MS sildenafil reviews (Stratton et al. [50] and Xiao et al. [53]), in the SCI-focused network meta-analysis by Tienforti et al. [56], and in a subset of more recent condition-specific reviews in stroke, SCI, PD, and MS (Brandão et al. [61], Chochina et al. [67], Gao et al. [71], Dunya et al. [73], Yavas et al. [70], and García-Perdomo et al. [55]). In contrast, many influential narrative or mixed-methods syntheses of neurogenic SD did not report any prior protocol (Lombardi et al. [48], Del Popolo et al. [49], Pöttgen et al. [52], and Afshar et al. [57]), limiting transparency and increasing susceptibility to selective methods or outcome reporting.

Similarly, documentation of excluded studies with reasons was rare. Only three reviews provided a detailed table of excluded full texts (Item 7 “Yes” in 3/26), notably the Cochrane reviews by Stratton et al. [50] and Xiao et al. [53] and one additional high-quality review, whereas the majority reported only a flow diagram or narrative description without a structured list (e.g., Del Popolo et al. [49], Esteve-Ríos et al. [59], Auger et al. [60], Parittotokkaporn et al. [72]). This limitation reduces reproducibility and makes it difficult to fully assess selection bias at the review level.

Most reviews did undertake some form of risk-of-bias or quality assessment of primary studies (Item 9 “Yes” in 19/26), often using Cochrane tools or condition-specific checklists in MS, SCI, PD, epilepsy and stroke (Stratton et al. [50], Xiao et al. [53], Tienforti et al. [56], Gopal et al. [58], Dunya et al. [73], Yavas et al. [70], and Gao et al. [71]). Nonetheless, five reviews did not apply a formal tool, relying instead on informal judgments or not reporting quality assessment at all (Lombardi et al. [48], Del Popolo et al. [49], Auger et al. [60], McLoughlin et al. [65], and Afferi et al. [68]). Two further reviews provided only partial or non-validated grading schemes (Giannopapas et al. [51] and Pöttgen et al. [52]). A particularly consistent weakness concerned reporting of funding and conflicts of interest in primary studies (Item 10), which were systematically collected in none of the reviews and only partially summarised in three (Stratton et al. [50], Xiao et al. [53], and Tienforti et al. [56]). For most syntheses, including several recent meta-analyses in SCI, MS and PD (García-Perdomo et al. [55], Jia et al. [54], Bahadori et al. [63], Parittotokkaporn et al. [72], and Couper et al. [66]), potential financial conflicts at the trial level remain poorly characterised.

Where meta-analysis was feasible, statistical methods were generally appropriate. Half of the reviews conducted at least one quantitative synthesis (Item 11 “Yes” in 13/26), including RCT-based pairwise meta-analyses and, in one case, network meta-analysis (Xiao et al. [53], Stratton et al. [50], García-Perdomo et al. [55], Jia et al. [54], Tienforti et al. [56], Gopal et al. [58], Couper et al. [66], Gao et al. [71], Yavas et al. [70]). However, only a minority clearly described how primary-study risk of bias influenced their synthesis and conclusions (Item 12 “Yes” in 5/26). Explicit integration of RoB and, where used, GRADE into interpretation was clearest in Cochrane contributions (Stratton et al. [50], Xiao et al. [53]) and in the best-performing network meta-analysis (Tienforti et al. [56]); other reviews often acknowledged limitations qualitatively without formally linking RoB judgments to effect estimates (e.g., Afshar et al. [57], Afferi et al. [68]).

Interpretation of results in light of study limitations (Item 13) was rated as adequate in most reviews (20/26 “Yes”), with many authors providing at least a narrative discussion of methodological weaknesses and generalisability (Lombardi et al. [48], Del Popolo et al. [49], Afshar et al. [57], Dunya et al. [73], and Couper et al. [66]). Heterogeneity was formally examined in the subset of reviews that undertook meta-analysis (Item 14 “Yes” in 12/26), particularly in the Cochrane and network meta-analytic work (Stratton et al. [50], Xiao et al. [53], Tienforti et al. [56], García-Perdomo et al. [55], Jia et al. [54]). By contrast, the assessment of publication bias and small-study effects was one of the most notable gaps: only two reviews carried out formal tests or funnel plot inspection, five discussed publication bias qualitatively, and 19 provided no assessment (Item 15). This limitation was evident across older SCI-focused meta-analyses and more recent MS- and PD-specific reviews (García-Perdomo et al. [55], Jia et al. [54], Esteve-Ríos et al. [59], Dunya et al. [73], Bahadori et al. [63]). Almost all reviews, however, reported their own funding sources and conflicts of interest transparently (Item 16 “Yes” in 25/26), including recent high-quality contributions (Tienforti et al. [56], Brandão et al. [61], Couper et al. [66]).

All in all, these AMSTAR 2 findings indicate that the current review-level evidence base on sexual health interventions in neurological disorders is methodologically fragile, with most syntheses affected by multiple critical flaws, particularly lack of protocol registration, incomplete reporting of excluded studies, limited or informal risk-of-bias assessment of primary studies, and sparse consideration of publication bias. In the context of our umbrella review, bodies of evidence dominated by critically low-confidence reviews, for example, many narrative overviews and condition-specific syntheses in MS, SCI, and stroke (Lombardi et al. [48], Del Popolo et al. [49], Giannopapas et al. [51], Pöttgen et al. [52], Afshar et al. [57], Esteve-Ríos et al. [59], Dunya et al. [73]), were treated as hypothesis-generating and were typically downgraded for risk of bias in the GRADE framework [46]. Conversely, the small group of higher-quality reviews, particularly the Cochrane RCT-based syntheses in post-stroke SD and MS sildenafil (Stratton et al. [50], Xiao et al. [53]) and the PROSPERO-registered SCI network meta-analysis (Tienforti et al. [56]), provided the main support for the highest certainty ratings in this umbrella review.

As a result, the strength of clinical inferences that can be drawn about pharmacological, psychological, rehabilitation-based, and multidisciplinary interventions for SD after neurological disorders is constrained not only by the limitations of primary studies but also by substantial heterogeneity and frequent methodological weaknesses at the review level. Given the predominance of critically low and low AMSTAR 2 confidence, pooled estimates and narrative conclusions were interpreted cautiously, and clinically directive statements were limited to intervention–outcome pairings supported by consistent trial-level effects and at least moderate review-level credibility.

### 3.2. SWiM-Structured Narrative Synthesis

The narrative synthesis followed the SWiM framework and treated each systematic review as the primary unit of analysis [47]. Evidence was grouped by neurological diagnosis, including SCI, MS, stroke and ABI, PD, epilepsy, CP, neuromuscular disorders, and mixed central or peripheral neuropathies [48,49,50,51,52,53,54,55,56,57,58,59,60,61,62,63,64,65,66,67,68,69,70,71,72,73]. Within each diagnostic group, interventions were classified as pharmacological, psychological or psychobehavioral, rehabilitation or physiotherapy based, and multidisciplinary or pathway oriented. To improve interpretability across heterogeneous reports, outcomes were characterized by domain whenever possible, distinguishing physiological sexual function endpoints (e.g., erectile function, arousal/lubrication, and orgasm/ejaculation), broader subjective outcomes (e.g., satisfaction and distress), and relational outcomes (e.g., intimacy and relationship quality). Quantitative findings from meta-analyses and trial-level summaries were described according to the reported direction and approximate magnitude of effects and were integrated with qualitative information on study design and populations from each review.

The most coherent body of evidence across conditions concerned PDE5 inhibitors for neurogenic erectile dysfunction in men with SCI [48,49,54,55,56,64,68]. Several condition-specific meta-analyses and one recent network meta-analysis consistently reported large short-term improvements in erectile function scores, successful intercourse attempts, and patient-reported treatment satisfaction with sildenafil, tadalafil, and vardenafil compared with placebo [54,55,56,64,68]. Evidence from intracavernosal injections, vacuum devices, and penile prostheses also indicated high rates of technically [48,49,50,51,52,53,54,55,56,57,58,59,60,61,62,63,64,65,66,67,68,69,70,71,72,73] successful erections and restoration of penetrative capacity, although the underlying studies were predominantly uncontrolled series at high risk of bias [48,49,64,67,68,69]. Outside SCI, pharmacological evidence was sparser and more heterogeneous, with only a few small trials in MS, stroke, and PD [48,49,50,51,52,53,57,60,63,71].

MS, stroke, and PD were mainly informed by small randomized or quasi-experimental studies and observational cohorts, often embedded in broader rehabilitation programs [50,51,52,57,58,59,60,61,62,63,70,71]. Reviews of psychological and psychoeducational interventions, pelvic floor and physiotherapy-based approaches, and multidisciplinary sexual rehabilitation frequently reported improvements in sexual function, intimacy, relationship satisfaction, or sexual distress in intervention groups, yet effect estimates were rarely precise and were difficult to compare because of variability in intervention content, intensity, and duration [52,58,59,60,61,70]. Women, couples, and partners were markedly underrepresented, and only a minority of trials focused specifically on female SD or provided sex-stratified analyses [51,57,59,63,70,73]. Follow-up durations were usually short, most often between four and twelve weeks, so longer-term maintenance of benefits on sexual function and quality of life remains uncertain across conditions [48,49,50,54,55,56,57,58,59,63,64,65,66,70,71,72,73]. Consistent with reviewer concerns regarding pediatric scope, the evidence base for pediatric/adolescent intervention effects could not be summarized as supported findings; instead, where childhood-onset conditions appeared in review-level evidence, they primarily reflected gaps rather than replicable interventional signals.

The synthesis relied entirely on pooled estimates and narrative results reported in the source reviews [47,50,53,54,55,56,57,58,60,61,63,66,70,71,72]. AMSTAR 2 ratings and primary study risk of bias assessments informed GRADE-based certainty judgments for each intervention–outcome pairing, and these certainty ratings were integrated into the wording of the condition-specific summaries [43,46,48,49,50,51,52,53,54,55,56,57,58,59,60,61,62,63,64,65,66,67,68,69,70,71,72,73]. Bodies of evidence derived mainly from high- or moderate-confidence Cochrane or PROSPERO-registered reviews, such as PDE5 inhibitors in SCI and selected psychobehavioral or physiotherapy interventions in MS and stroke, supported higher certainty ratings [50,53,56,58,59,61]. Evidence based on systematic reviews with a narrative approach in synthesis evidence at critically low confidence, small uncontrolled trials, or observational cohorts without appropriate comparators was graded as low or very low certainty and was interpreted as hypothesis generating rather than confirmatory [48,49,51,52,57,58,59,60,63,67,68,69,70,71,72,73]. Table 2 summarises, by neurological condition, the intervention categories, main outcome domains, direction of effect, and umbrella-level certainty ratings.

### 3.3. Restoring Sexual Function After Spinal Cord Injury: Pharmacological and Device-Based Interventions

Systematic reviews on SCI and broader neurogenic SD describe the most extensive intervention evidence base in this umbrella review [48,49,54,55,56,64,67,68,69,72]. Most primary studies enrolled adult men with chronic SCI and erectile dysfunction in specialized neurourology or rehabilitation units, whereas women and pediatric cohorts were rarely represented [48,49,64,68,72]. Pharmacological interventions, especially PDE5 inhibitors, dominated the literature, with additional data on intracavernosal injections, vacuum erection devices, penile prostheses, testosterone replacement, and neuromodulatory or stimulation-based techniques [48,49,54,55,56,64,67,68,69,72]. Review-level quality was mixed. SCI-specific meta-analyses and a recent network meta-analysis were rated between moderate and high confidence, while broader narrative overviews were commonly judged critically low because of incomplete protocols, limited risk of bias assessment, and lack of formal synthesis [48,49,54,55,56,64,67,68,69].

Placebo-controlled randomized trials and pooled analyses of PDE5 inhibitors showed large short-term improvements in erectile function indices, the ability to achieve and maintain erections sufficient for intercourse and global sexual satisfaction in men with SCI [48,49,54,55,56,64,68]. The network meta-analysis by Tienforti et al. [56], which included ten randomized trials and almost fifteen hundred men, reported high response rates for sildenafil, tadalafil, and vardenafil, with successful intercourse often achieved by more than sixty percent of participants [56]. Adverse events were usually mild or moderate, including headache, flushing, and dyspepsia, with very few serious harms reported [48,49,54,55,56,64,68]. These findings supported a moderate certainty rating that PDE5 inhibitors probably improve erectile function and intercourse success in men with SCI-related erectile dysfunction. Evidence on durability beyond three to six months remained limited, so long-term effects on sexual quality of life and partner outcomes are still uncertain [48,49,54,55,56,64,68].

Intracavernosal injections with papaverine, papaverine plus phentolamine, or prostaglandin E1, as well as vacuum erection devices and penile prostheses, achieved high rates of erections sufficient for intercourse in case series and nonrandomized comparative studies, with response rates frequently above eighty percent [48,49,64,67,68,69]. The evidence for these modalities was predominantly single-center and unblinded and was based on small samples, with selection bias and incomplete adverse event reporting [64,67,68,69]. Local pain, hematoma, fibrosis, and priapism were among the most frequent complications of injection therapies, while mechanical failure and infection were reported after penile prosthesis implantation [64,67,68,69]. These interventions were judged to have potential benefit as second line options, although certainty of evidence for their impact on patient-centered sexual outcomes remained low to very low.

Testosterone replacement in men with SCI or TBI, often combined with functional electrical stimulation or resistance training, was evaluated in small trials and prospective cohorts that primarily targeted musculoskeletal and metabolic outcomes [64,65]. Sexual function outcomes were usually secondary and showed inconsistent changes, with some improvement in libido and erectile function but no stable pattern in validated scales [64,65]. Neuromodulatory and stimulation-based interventions, including sacral neuromodulation, perineal electrostimulation, penile vibratory stimulation, and electroejaculation, were mainly examined for ejaculatory function or combined neurogenic bowel, bladder, and sexual outcomes [48,64,69,72]. These techniques achieved high ejaculation success rates in selected men, yet the supporting studies were small and heterogeneous and were often at high risk of bias [69,72]. Certainty ratings for testosterone therapy and neuromodulatory interventions were consequently very low, and their effects on broader sexual function and sexual quality of life remain uncertain.

Interventions for SD after SCI are better studied than in other neurological conditions, yet important gaps persist. Women with SCI, couples and partners, and outcomes beyond erectile function, such as satisfaction, intimacy, and relationship quality, were sparsely evaluated and mostly derived from small observational studies [48,49,64,68,72]. Evidence on pediatric-onset SCI and on culturally diverse populations was virtually absent. Within this context, robust evidence is essentially confined to short-term pharmacological management of neurogenic erectile dysfunction in adult men with SCI.

### 3.4. Multimodal Strategies for Sexual Dysfunction in Multiple Sclerosis

Men predominated in pharmacological trials, whereas women were more frequently included in psychobehavioral and pelvic floor-focused interventions [51,57,58,59,70,73]. Review-level confidence was generally low or critically low because of limited protocols, incomplete reporting of excluded studies, and mixed study designs. A Cochrane review of sildenafil for erectile dysfunction and a large meta-analysis of mixed interventions provided the most robust anchors for certainty assessments [53,57].

Pharmacological evidence concerned mainly PDE5 inhibitors. The Cochrane review by Xiao et al. [53] identified six placebo-controlled randomized trials of sildenafil, tadalafil, or related agents in men with MS-related erectile dysfunction and reported clinically important improvements in erectile function scores and intercourse success compared with placebo [53]. Adverse events were similar to those observed in SCI populations, dominated by headache, flushing, and gastrointestinal discomfort, without new safety signals [48,49,53,57]. Subsequent narrative reviews and the broader meta-analysis by Afshar et al. [57] supported these findings but emphasized that the overall sample was small, follow-up was short, and trial quality was moderate, with occasional unclear allocation and blinding [51,57]. Certainty was rated as moderate that PDE5 inhibitors probably improve erectile function in men with MS, while certainty was low for any effect on broader sexual satisfaction, relationship outcomes, or female SD because of sparse data [51,53,57,58,59,73].

Psychological and psychobehavioral interventions included individual and couples counselling, cognitive behavioural therapy, PLISSIT model programs, and structured psychoeducation targeting communication, body image, and intimacy [52,57,58,59,73]. Systematic reviews by Pöttgen et al. [52], Esteve Ríos et al. [59], and Dunya et al. [73] identified a small set of randomized and quasi-experimental trials and observational series that suggested improvements in sexual function scores, sexual satisfaction, and sexuality-related distress in participants receiving structured psychosexual interventions compared with usual care or waitlist control [52,59,73]. Some programs involved partners and reported gains in relationship satisfaction and communication, although partner outcomes were inconsistently measured [59,73]. Trials were small, often single-center, and at substantial risk of performance and detection bias, and meta-analytic pooling was rarely feasible [52,59,73]. These limitations supported a low certainty rating that psychobehavioral interventions may improve sexual function and related psychosocial outcomes in people with multiple sclerosis.

Physiotherapy-based interventions, particularly pelvic floor muscle training with or without biofeedback or electrostimulation, were examined in meta-analyses by Gopal et al. [58] and Yavaş et al. [70]. A limited number of randomized and controlled studies suggested that pelvic floor programs were associated with moderate to large improvements in sexual function indices, especially in women, and with parallel benefits in urinary symptoms and health-related quality of life [58,70]. Adherence was generally high, and adverse events were rare, usually limited to transient discomfort during exercises or stimulation [58,70]. Methodological quality was mixed, with several trials at high or unclear risk of bias because of inadequate concealment, lack of blinding, and incomplete outcome data [58,70]. Certainty was therefore rated as low to moderate that pelvic floor muscle training and related physiotherapy interventions probably improve sexual function in adults with MS who also experience pelvic floor or lower urinary tract dysfunction.

Taken together, the MS literature suggests that a range of pharmacological, psychobehavioral, and physiotherapy-based interventions may alleviate SD, although the strength of evidence is uneven. Men with erectile dysfunction appear to benefit most consistently from PDE5 inhibitors, while women may gain from psychosexual and pelvic floor programs [51,52,53,57,58,59,70,73]. Evidence for couples-focused therapies, multidisciplinary pathways, and long-term outcomes remains scarce, and most trials report follow-up of only a few months [52,57,58,59,70,73]. Certainty ratings were seldom higher than moderate and were often low or very low for female-specific outcomes, secondary psychological endpoints, and complex intervention packages.

### 3.5. Post-Stroke and Acquired Brain Injury Sexual Rehabilitation: Psychological and Pharmacological Approaches

Systematic reviews in stroke and ABI populations focused mainly on psychological and educational interventions, with very limited evidence on pharmacological treatments [50,60,61,62]. The Cochrane review by Stratton et al. [50] identified only a few heterogeneous randomized trials targeting SD after stroke, including sertraline for premature ejaculation, sildenafil for erectile dysfunction, and brief sexual rehabilitation programs integrated into stroke rehabilitation [50]. Samples were small, follow-up was short, and risk of bias was frequently unclear, so pooled quantitative synthesis was not undertaken [50]. Subsequent reviews by Auger et al. [60], Brandão et al., [61], and Dusenbury et al. [62] added observational and quasi-experimental studies, yet overall confidence remained low because of methodological weaknesses and the predominance of male participants [60,61,62].

Pharmacological interventions after stroke were evaluated in a small number of trials. Sertraline for premature ejaculation appeared to prolong intravaginal ejaculation latency time and improve perceived control compared with placebo, although sexual satisfaction outcomes were not consistently reported and durability beyond the treatment period was unknown [50]. Small studies of sildenafil and related agents for erectile dysfunction suggested possible improvements in erectile function scores, but effect estimates were imprecise and often based on single-center samples [48,50,60]. Adverse events seemed consistent with known drug profiles, without new safety signals, although numbers were too small to inform rare harms [50,60]. Certainty was therefore rated as low or very low that pharmacological treatments may improve selected aspects of sexual function after stroke.

Psychological and psychoeducational interventions were more consistently represented. Auger et al. [60] and Brandão et al. [61] described structured sexual rehabilitation programs that combined education, counselling, communication training, and, in some cases, couples sessions delivered by multidisciplinary teams [60,61]. Across a small number of randomized and controlled studies, these interventions appeared to increase sexual knowledge, reduce fear of resuming sexual activity, and improve self-reported sexual satisfaction or coital frequency compared with usual care [60,61]. Effects on specific domains such as desire, arousal, and orgasm, and on partner outcomes, were inconsistent and often underpowered [60,61,62]. Follow-up rarely exceeded six months [60,61,62]. Certainty was therefore rated as low that psychological and psychoeducational programs may improve sexual satisfaction and readiness to resume sexual activity after stroke.

ABI of traumatic or non-traumatic origin other than stroke was seldom addressed. Most reviews either merged these populations with stroke or provided only brief narrative comments, and dedicated rehabilitation trials for SD after ABI were scarce [48,60,61,62]. Certainty was consequently rated as very low for any intervention effect estimates in acquired brain injury, including outcomes related to intimacy, mood, and relationship quality.

### 3.6. Sexual Dysfunction in Parkinson Disease, Epilepsy, Cerebral Palsy and Neuromuscular or Peripheral Neuropathies: A Fragmented Evidence Base

Systematic reviews addressing neurological conditions other than SCI, MS, and stroke highlighted a fragmented and predominantly observational evidence base [48,49,58,59,60,63,64,65,66,69,70,71,72,73]. In PD, most data are derived from cohorts undergoing subthalamic nucleus deep brain stimulation. Reviews by Bahadori et al. [63] and Gao et al. [71] reported small and inconsistent changes in erectile function or sexual satisfaction, and several cohorts showed no clear benefit at all [63,71]. Sexual outcomes were secondary endpoints and were confounded by disease severity and medication changes, so certainty was rated as very low that deep brain stimulation has any consistent effect on sexual function in PD [63,71].

Evidence in epilepsy came mainly from observational studies of antiseizure medications. The review by Couper et al. [66] described associations between enzyme-inducing drugs, reduced testosterone levels, and erectile dysfunction in men, whereas newer agents such as lamotrigine and levetiracetam appeared comparatively neutral [66]. These findings suggested medication-related contributors to SD, but the absence of randomized switching trials and limited measurement of sexual outcomes yielded low certainty that altering anti-seizure regimens improves sexual health [66].

Reviews of CP, neuromuscular disorders, peripheral neuropathies, and mixed neurogenic SD underscored the near absence of dedicated intervention trials [48,49,58,59,60,69,72,73]. Most available studies reported prevalence or correlates of sexual difficulties, while treatment data were restricted to isolated case series or extrapolations from adult SCI populations [48,49,60,69,72]. Interventions embedded within broader neurorehabilitation pathways, including education and counselling, may offer benefits for communication and coping, yet evidence on sexual function, satisfaction, and partner outcomes remains scarce and methodologically weak, so certainty for all intervention–outcome pairings in these populations was judged very low [58,59,60,72,73].

## 4. Discussion

According to this umbrella review, sexual health is a core yet under-addressed dimension of life after neurological disorders. SD is highly prevalent across SCI, MS, stroke, PD, epilepsy, and other conditions, often persisting over time and substantially affecting quality of life and relationships [8,9,10,11,12,13,14,15,16]. Importantly, this umbrella review was designed to map and qualify the strength of intervention evidence within condition-specific pairings rather than to rank treatments across disorders or issue comparative effectiveness guidance across heterogeneous neurological populations. Pharmacological, psycho-behavioral, physiotherapy-based, and multidisciplinary interventions have been evaluated, but evidence depth and robustness differ across diagnostic groups and intervention classes. The strongest and most coherent signals concern PDE5 inhibitors for neurogenic erectile dysfunction in men with spinal cord injury, supported by several meta-analyses including one network meta-analysis, whereas evidence for other approaches and populations is fragmented [48,49,53,54,55,56,64,68]. Many systematic reviews were judged at low or critically low confidence on AMSTAR 2, and primary trials frequently had substantial risk of bias, small samples, and short follow-up [48,49,50,51,52,57,58,59,60,61,62,63,70,71,72,73]. GRADE-based certainty ratings were therefore moderate only in a few domains and low or very low for most intervention–outcome pairings, constraining inferences about effectiveness and safety. This discussion interprets these findings in a broader clinical and neurophysiological context and outlines implications for practice and research.

### 4.1. Interpreting a Fragmented Landscape of Sexual Rehabilitation: A Qualitative Analysis

The qualitative synthesis highlights a marked imbalance in the therapeutic evidence base across neurological conditions. Interventions for male erectile dysfunction after SCI represent the most mature field: randomized trials show large short-term improvements in erectile function and intercourse success with PDE5 inhibitors [54,55,56,64,68,74,75,76,77]. A crossover trial indicates that tadalafil does not produce clinically relevant adverse cardiovascular responses in men with high thoracic lesions, supporting short-term safety in this group [78]. The consistency of effect estimates, plausible mechanisms, and acceptable safety justify moderate certainty that PDE5 inhibitors probably improve erectile function and intercourse success in men with SCI. In contrast, evidence for second-line strategies such as intracavernosal injections, vacuum devices, and penile prostheses, although suggesting substantial technical efficacy, comes largely from uncontrolled series and observational cohorts, keeping certainty low or very low for patient-centered outcomes [64,67,68,69]. Clinically, a pragmatic interpretation is that PDE5 inhibitors can be considered first-line for adult men with SCI-related erectile dysfunction when not contraindicated, while devices or injections may be considered when PDE5 inhibitors are ineffective or not tolerated, with explicit counselling that patient-centred outcomes and longer-term relational benefits remain uncertain.

In MS, sexual health interventions form a more diversified but methodologically weaker landscape. PDE5 inhibitors again appear beneficial for men with erectile dysfunction, although trials are few and follow-up is short [51,53,57,79]. Cross-sectional and cohort data confirm that SD is highly prevalent, associated with disease burden and vascular risk factors, and linked to poorer health-related quality of life and psychological well-being [80,81]. Narrative syntheses emphasize the multifactorial nature of MS-related SD and the need for integrated pharmacological, rehabilitative, and psychosocial management rather than isolated symptom treatment [82]. Trials of pelvic floor-oriented and psychoeducational programs remain scarce but suggest that structured interventions can improve sexual satisfaction, communication, and psychological outcomes in women with MS [58,70,83]. Overall, these bodies of evidence support low to at best moderate certainty that multifaceted programs may help some people with MS, while durability, optimal components, and generalizability remain uncertain. Clinically, PDE5 inhibitors can be discussed with moderate certainty for male erectile dysfunction, whereas pelvic floor and psychosexual programmes may be considered for selected patients, particularly when pelvic floor or urinary symptoms, distress, or communication difficulties are prominent, while communicating that certainty is lower outside erectile endpoints.

A clear distinction emerges between a small set of findings that can support clinical discussions with relatively greater confidence and a much larger body of evidence that remains hypothesis-generating. In practical terms, PDE5 inhibitors for erectile dysfunction after SCI represent the most consistent signal, whereas most psychobehavioral, physiotherapy-based, and multidisciplinary interventions are supported by small trials with heterogeneous outcomes and limited follow-up, which constrains both generalizability and durability of effects.

Stroke and ABI illustrate an even more fragmented picture. Psychological and educational interventions, including structured sexual rehabilitation programs and couples-based retreats, suggest benefits for sexual knowledge, readiness to resume activity, and perceived intimacy, with randomized and quasi-experimental trials showing short-term improvements in satisfaction and intercourse frequency [8,60,61,62]. However, samples are modest and long-term data scarce. Pharmacological trials for erectile dysfunction or premature ejaculation after stroke are rare, underpowered, and not pooled, limiting inferences [48,50]. For other conditions such as PD, epilepsy, cerebral palsy, neuromuscular disorders, and peripheral neuropathies, evidence still comes mainly from prevalence cohorts and narrative reviews, and treatment strategies are often extrapolated from other populations [58,59,60,63,64,65,66,69,70,71,72,73,82]. Clinically, the most supportable message is that post-stroke/ABI interventions should be framed as targeted support for knowledge, readiness, and adaptation, while medication-based strategies may be trialed cautiously when clear symptom targets exist, with transparent acknowledgement of low/very low certainty and limited long-term data.

#### Gender, Inclusivity, and Outcome Imbalance

Sex-related imbalance represents a structural limitation of the evidence base and directly affects external validity. Across included reviews, 10/26 restricted inclusion to men, and women were represented in 16/26; nevertheless, validated female sexual function outcomes were synthesized in only 6/26 reviews, and relationship/couple outcomes in 3/26. This pattern indicates that much of what is labelled as “sexual rehabilitation” in neurological populations is still operationalized through male erectile endpoints, while broader domains of sexuality, partner experience, and female-specific function remain under-measured. Future trials should therefore pre-specify sex-stratified analyses and adopt outcome sets that capture satisfaction, desire, pain, and relational functioning alongside physiological markers [84,85,86,87,88,89]. Studies in women with MS, stroke, or PD show high rates of desire and arousal difficulties, orgasmic problems, and sexual distress linked to mood, fatigue, coping, and endocrine changes, yet tailored interventions remain rare [63,70,73,87,88,89]. Recent MS cohorts indicate that SD, fatigue, and depression jointly drive poorer quality of life, suggesting that narrow symptom-based approaches may miss key sources of distress [87,88]. Data on non-heterosexual and gender-diverse individuals are almost absent. These gaps contribute directly to lower certainty for many outcomes because estimates derived largely from male erectile endpoints cannot be assumed to generalise to women’s sexual function, distress, or couple-level outcomes; therefore, interpretation should remain conservative outside the relatively well-studied erectile function domain.

Follow-up duration represents a further limitation. Most trials evaluate outcomes over weeks or a few months, whereas sexual adaptation after neurological disorders unfolds over years and interacts with disease progression, comorbidities, and life transitions [54,55,56,57,58,59,63,64,65,66,70,71,72,73,80,81,82]. Adverse events are usually monitored only in the short term and reported incompletely, especially in psychobehavioral and complex rehabilitation interventions. These features support cautious interpretation of positive findings and highlight the need for trial designs that address long-term maintenance, relapse, and real-world adherence.

A further gap concerns developmental stages. Although sexual health needs are clinically relevant in adolescents and young adults with childhood-onset neurological conditions, no included systematic review synthesized pediatric intervention trials. The absence of review-level intervention evidence in this subgroup should be stated as an evidence gap rather than as a supported conclusion and limits any condition-specific clinical recommendations for pediatric populations.

### 4.2. Shared Challenges and Condition-Specific Contrasts

Condition-specific contrasts shape both the expression of SD and intervention opportunities. SCI often causes neurogenic erectile dysfunction and ejaculatory failure with preserved desire, especially in younger men, which aligns with pharmacological approaches acting on peripheral hemodynamics [69,72,74,75,76,77,78]. MS and PD are characterized by fluctuating symptoms, fatigue, spasticity, and autonomic dysfunction that affect desire, arousal, and orgasm in both sexes and interact with mood and medication effects [70,73,80,81,82,87,88,89]. Large MS cohorts confirm that SD is common in women and men, associated with disability, depression, and fatigue, and strongly linked to quality of life [80,81,82,87,88,89]. Stroke and ABI frequently involve cognitive, communicative, and emotional changes that reshape intimacy and couple dynamics even when genital function is relatively preserved [50,60,61,62]. In epilepsy, antiseizure medications, endocrine pathways, and psychosocial factors interact, and cross-sectional studies report high rates of reduced desire, erectile dysfunction, and orgasmic difficulties [66,90]. Clinically, these contrasts imply that mechanistic “targets” differ by condition: erectile function may be the primary target in SCI, whereas desire, distress, fatigue, medication effects, communication, and couple adaptation often represent key targets in MS, PD, stroke/ABI, and epilepsy.

Measurement practices further contribute to heterogeneity and limit cross-study comparison. Many trials rely on erectile function scales or nonvalidated questionnaires, with less frequent use of multidimensional, gender-sensitive instruments that capture desire, arousal, orgasm, pain, satisfaction, and distress [70,73,80,82]. Partner outcomes, couple communication, and relationship quality are seldom assessed, despite qualitative evidence that they are central to adjustment after SCI and stroke [59,60,61,84,85,86]. Life course and intersectional perspectives are underrepresented. MS cohorts suggest that disease duration, comorbidity burden, and social support modulate the impact of SD on quality of life [80,81,82,87,88,89]. Chronic illness frameworks highlight how cultural norms, gendered expectations, and socioeconomic constraints shape sexual well-being and help-seeking, particularly for older adults, migrants, and people in resource-constrained settings, which limits generalizability and should inform future trial design [13,14,15,16,91].

Methodological limitations in the primary and secondary literature amplify these concerns. Many systematic reviews combine randomized, quasi-experimental, and observational designs without stratified analyses or sensitivity checks, and overlap between primary studies is frequent and seldom quantified [57,58,59,60,61,62,63,70,71,72,73]. Trials often recruit small single-center samples, use unclear randomization and concealment procedures, and incompletely report attrition and adverse events [57,58,59,60,61,62,63,70,71,72,73]. Narrative and scoping work in stroke and epilepsy suggests that only a minority of people who report sexual concerns receive structured assessment or evidence-based intervention [90,92,93]. These features contribute to imprecision and possible publication bias, underpinning the predominantly low or very low GRADE ratings for most intervention–outcome pairings. The few domains with stronger design features, such as PDE5 treatment of erectile dysfunction in SCI and MS, illustrate what more robust trial programs might achieve if extended to female SD, couple-based outcomes, and understudied conditions.

### 4.3. Neurophysiological Pathways Linking Neurological Damage, Sexual Dysfunction and Quality of Life

Neurophysiological considerations help integrate heterogeneous findings and explain why intervention effects vary by condition. Sexual arousal and response rely on coordinated cortical, limbic, brainstem, and spinal networks mediated by autonomic and somatic pathways [69,72,94,95,96,97]. Thus, supratentorial lesions more often disrupt desire and emotional processing, whereas infratentorial or spinal lesions more directly impair genital arousal, lubrication, erection, and ejaculation [72,94,95,96,97]. In MS and PD, combined involvement of central networks and autonomic outflow contributes to multi-domain SD phenotypes and helps interpret outcome-specific, heterogeneous treatment signals [80,81,82,87,88,89,98,99].

SCI illustrates a direct mechanism–treatment link. Lesions above sacral segments may preserve reflexogenic erections while disrupting psychogenic erections, whereas lower lesions may impair both mechanisms [48,49,64,69,72]. Accordingly, downstream approaches (intracavernosal injections, vacuum devices, penile prostheses) can induce erections despite severely compromised supraspinal input [64,67,68,69], while PDE5 inhibitors, facilitating nitric oxide-mediated vasodilation, typically require partial neural preservation, plausibly explaining variable responses by lesion completeness [64,68,74,75,76,77,78]. In MS and PD, pathological changes across cortical networks, basal ganglia, hypothalamus, and autonomic pathways, together with medication effects, further shape SD and should be considered when interpreting intervention effects and counselling [73,80,81,82,87,88,89,98,99]. PD cohorts also report dopaminergic treatment-related hypersexuality in a subset [98,99], reinforcing the importance of medication review. In epilepsy, antiseizure drugs may alter sex hormones and related axes, and narrative work highlights endocrine, affective, and stigma-related contributors to SD [90,93], supporting optimization and rationalization of drug regimens as part of management. Because SD is tightly linked to mood and participation, quality of life functions as both outcome and mediator in neurological SD [80,81,82,87,88,89,100,101].

Consistent with this integrated model, sexual difficulties rank among the most distressing consequences in MS and SCI [87,88,89,100,101], and cross-sectional SCI studies highlight persistent needs in communication, continence management, and pain control that are plausible targets for rehabilitation-oriented interventions [100,101]. Qualitative work shows that body image, mobility, and communication reshape intimacy after SCI and stroke [59,60,61,84,85,86], providing a rationale for multimodal approaches addressing relational and psychosocial determinants alongside physiological endpoints. While evidence remains limited, these approaches align with mechanistic models and represent a priority for future trials [102,103].

### 4.4. From Evidence to Care: Clinical Pathways and Research Priorities

The findings of this umbrella review support several practical messages for clinicians. Sexual health should be treated as a fundamental component of neurological and rehabilitation care rather than an optional add-on. Routine, nonjudgmental inquiry about sexual function and intimacy, supported by brief screening tools and structured interview guides, can normalize the topic and identify unmet needs, while early referral to specialized sexual counselling or rehabilitation services provides more comprehensive support [60,61]. Clinically, a domain-based approach is useful: clinicians can distinguish primary erectile function concerns, desire/arousal and distress concerns, and relational/intimacy concerns, because these domains align with different intervention types and with different levels of certainty across conditions. Pharmacological treatment of erectile dysfunction in men with SCI and MS can be offered with reasonable confidence regarding short-term efficacy and safety, accompanied by counselling on expectations, contraindications, and the potential need for second-line devices or injections [53,54,55,56,64,68]. For men with SCI specifically, PDE5 inhibitors represent the clearest moderate-certainty option for erectile function, whereas device-based or injection therapies may be discussed as escalation strategies when first-line treatment is ineffective or not tolerated, while acknowledging lower certainty for broader quality-of-life and partner outcomes.

Psychobehavioral, physiotherapy-based, and multidisciplinary interventions should be framed as promising but not yet supported by high-certainty evidence. Clinicians may still consider them when patients express interest and local expertise is available, particularly when potential benefits extend to continence, mood, and overall rehabilitation engagement [52,58,59,60,61,70,73]. Clear communication about limited trial data, the need for sustained practice, and the role of partner involvement can help align expectations. Interdisciplinary collaboration between neurologists, physiatrists, urologists, gynecologists, psychologists, sex therapists, physiotherapists, and nurses is likely crucial for implementation [48,49,50,51,52,57,58,59,60,61,62,63]. Because training curricula in neurology and rehabilitation rarely cover sexual health systematically, targeted educational programs may improve clinician confidence and patient outcomes. Across conditions, clinicians can apply two consistent principles: when certainty is moderate (e.g., PDE5 inhibitors for male erectile dysfunction in SCI and MS), treatment can be presented as likely beneficial short-term; when certainty is low or very low, interventions should be presented as preference-sensitive options with transparent uncertainty, prioritizing shared decision-making and outcomes that matter to patients and partners.

The research agenda emerging from this review is extensive. Larger, methodologically rigorous multicenter trials are needed across a broader range of neurological conditions, with particular priority for women, couples, and pediatric and life course perspectives [57,58,59,63,70,73]. Trials should embed standardized, validated sexual health and quality-of-life measures that capture multiple domains and partner outcomes and should report adverse events and adherence transparently. Comparative effectiveness studies of combined pharmacological and psychobehavioral packages, together with implementation research on service models and cost-effectiveness, would help bridge the gap between efficacy and real-world practice [58,59,60,61,70,73]. Patient and partner involvement in the co-design of interventions and outcome measures is likely to improve relevance and uptake. Consistent with reviewer priorities, future trials should be sex- and gender-sensitive by design, adequately powered for women and diverse relationship structures, and should explicitly include partner/couple endpoints when the intervention target includes communication, intimacy, or relational adjustment.

### 4.5. Methodological Strengths and Limitations

This umbrella review is strengthened by a comprehensive multi-database search, prospective protocol registration, duplicate screening and extraction, and a SWiM-guided narrative synthesis that prioritized intervention evidence when meta-analysis was not appropriate [43,46,47,48]. The combined use of AMSTAR 2 and GRADE was intended to make the main message clinically interpretable: review credibility and trial-level limitations were explicitly translated into certainty statements for each intervention–outcome pairing, so that conclusions reflect not only the direction of effect but also how much confidence clinicians can place in that effect.

Key limitations arise from the umbrella design and from the quality of the underlying evidence base. First, we relied entirely on published systematic reviews and meta-analyses and did not re-extract primary trial data, meaning that any inaccuracies in study selection, extraction, or risk-of-bias assessment in the source reviews could not be corrected at this level. Second, overlap across reviews could not be quantified with a formal coverage metric because trial identifiers were inconsistently reported; overlap was therefore treated conservatively as a source of indirectness and potential double-counting and was reflected in certainty judgements [47,48,49,50,51,52,57,58,59,60,61,62,63,70,71,72,73]. Third, restricting inclusion to English-language peer-reviewed reviews may have excluded relevant evidence and should be considered when interpreting apparent gaps across conditions and populations.

The predominance of low or critically low AMSTAR 2 confidence, together with small samples, short follow-up, incomplete adverse event reporting, and frequent risk-of-bias concerns in primary studies, especially in psychobehavioral and complex rehabilitation interventions, contributed to predominantly low or very low certainty for many intervention–outcome pairings [47,48,49,50,51,52,57,58,59,60,61,62,63,70,71,72,73]. Additional uncertainty stems from heterogeneity in neurological phenotypes, intervention content and intensity, and outcome measurement, with many studies relying on non-harmonized instruments; this limited quantitative synthesis in the source reviews and increased reliance on structured narrative integration under SWiM [46,47,48].

Overall, these constraints mean the findings should be used as a certainty-informed map: identifying where evidence is sufficiently coherent to support cautious clinical use, where signals remain hypothesis-generating, and where major gaps—particularly regarding women, couples, diverse populations, and longer-term outcomes—require higher-quality trials and harmonized outcome sets [47,48,49,50,51,52,57,58,59,60,61,62,63,70,71,72,73].

## 5. Conclusions

This umbrella review indicates that interventional evidence for SD and broader sexual health outcomes after neurological disorders remains uneven and frequently limited by review-level and trial-level weaknesses. The most consistent signal concerns PDE5 inhibitors for erectile dysfunction in men with SCI, whereas most other interventions across MS, stroke/ABI, PD, and epilepsy are supported by low- or very low-certainty evidence that should be interpreted as hypothesis-generating. The evidence base is also structurally imbalanced, with limited synthesis of validated female sexual function outcomes and scarce relationship/partner measures and no review-level synthesis of pediatric intervention trials. Advancing the field will require inclusive trial designs, harmonized outcome sets that extend beyond erectile endpoints, transparent reporting, and longer follow-up embedded within multidisciplinary neurorehabilitation pathways.

## Figures and Tables

**Table 1:** Methodological overview of the umbrella review.

Section	Methodology	Details
Search Strategy	Databases and overall scope	PubMed, Web of Science, Cochrane Library, Embase, EBSCOhost (including PsycINFO where available), and Scopus were searched from inception to 27 November 2025. The strategy targeted systematic reviews and meta-analyses of sexual health interventions in adult and paediatric populations with neurological disorders, using controlled vocabulary (e.g., MeSH) and free-text terms tailored to each database.
Search Strategy	Conceptual blocks	The search was structured into three conceptual blocks aligned with PICO: (i) neurological conditions and neurorehabilitation populations; (ii) SD and sexual health outcomes; (iii) intervention and review design, including pharmacological and non-pharmacological treatments, rehabilitation, psychotherapy, sex therapy, and terms for systematic reviews and meta-analyses. Strings were adapted for each database with appropriate subject headings and review filters.
Search Strategy	Search String 1	(“Stroke” OR “Brain Injuries” OR “Spinal Cord Injuries” OR “Multiple Sclerosis” OR “Parkinson Disease” OR “Epilepsy”) AND (“Sexual Dysfunction, Physiological” OR “Sexual Dysfunctions, Psychological” OR “Erectile Dysfunction”) AND (“Rehabilitation” OR “Neurological Rehabilitation” OR “Psychotherapy” OR “Sex Counseling”).
Search Strategy	Search String 2	(“Stroke” OR “Brain Injuries” OR “Spinal Cord Injuries” OR “Multiple Sclerosis” OR “Parkinson Disease” OR “Epilepsy” OR “Cerebral Palsy” OR “Neuromuscular Diseases”) AND (“Sexual Dysfunction, Physiological” OR “Sexual Dysfunctions, Psychological” OR “Erectile Dysfunction” OR “Sexual Partners” OR “Marital Relations” OR “Interpersonal Relations”) AND (“Sex Counseling” OR “Psychotherapy” OR “Cognitive Behavioral Therapy” OR “Behavior Therapy” OR “Neurological Rehabilitation” OR “Physical Therapy Modalities”).
Search Strategy	Search String 3	(“Child” OR “Adolescent” OR “Cerebral Palsy”) AND (“Spinal Cord Injuries” OR “Epilepsy” OR “Brain Injuries” OR “Multiple Sclerosis” OR “Parkinson Disease” OR “Neurological Disorders”) AND (“Sexual Dysfunction, Physiological” OR “Sexual Dysfunctions, Psychological” OR “Sexuality” OR “Sexual Health”) AND (“Psychotherapy” OR “Sex Counseling” OR “Rehabilitation” OR “Sexual Interventions” OR “Pharmacological Treatments”).
Search Strategy	Search period and execution	No a priori date limits were applied. Searches covered database inception to 27 November 2025. Reference lists of included reviews and key narrative or guideline papers on sexual health in neurological disorders were screened, and forward citation tracking in Web of Science and Scopus identified additional and more recent reviews. Only peer-reviewed articles in English were eligible to enable duplicate screening and consistent appraisal across reviewers.
Study Selection	Screening process and PRISMA 2020	Study selection followed PRISMA 2020. Two reviewers (AM, AC) independently screened titles/abstracts using piloted forms, then assessed full texts against predefined criteria. A PRISMA 2020 flow diagram documented records identified, screened, excluded (with reasons), and included at each stage.
Study Selection	Inter-rater agreement and adjudication	Disagreements at either screening stage were resolved through discussion; unresolved cases were adjudicated by a third reviewer (RSC). Inter-rater agreement was quantified using Cohen’s κ with 95% confidence intervals and interpreted using conventional thresholds (κ ≥0.61 substantial, κ ≥0.81 almost perfect) [41].
Inclusion Criteria	Population and condition	Eligible populations were adults (≥18 years) and paediatric/adolescent patients (<18 years) with neurological disorders in which SD or impaired sexual health represented a direct or frequent consequence. Conditions included stroke, acquired brain injury (traumatic or non-traumatic), spinal cord injury, multiple sclerosis, Parkinson’s disease, epilepsy, cerebral palsy, neuromuscular disorders, and other central or peripheral nervous system diseases. Reviews of mixed clinical populations were eligible only when neurological data were reported separately or when ≥70% of participants had a neurological diagnosis; this threshold was operationalised using proportions reported by review authors at the level of pooled samples or included primary studies (e.g., mixed-etiology SD reviews were eligible when ≥70% of participants were drawn from neurological diagnoses such as SCI/MS/stroke, even if a minority reflected other etiologies). Several paediatric populations (including paediatric cerebral palsy) and multiple specific neuromuscular disorders lacked eligible systematic intervention reviews and were treated as evidence gaps rather than as synthesizable intervention effects.
Inclusion Criteria	Intervention and comparator requirements	Included reviews synthesised interventional studies where the primary aim was to prevent or manage SD or to improve sexual health in a neurological context. Interventions could be pharmacological, psychological/behavioural, rehabilitation-based, or multidisciplinary. Acceptable comparators included placebo or sham, attention control, usual care, no treatment, waiting list, or active alternative interventions. Pre–post designs without formal control were accepted if clearly treated as part of an intervention evidence base by review authors.
Inclusion Criteria	Outcomes, designs, and review-level requirements	Reviews had to report at least one patient-level sexual outcome (e.g., validated measures of sexual function and/or satisfaction), and could include randomised, quasi-experimental, or observational intervention designs. For eligibility and synthesis, SD was used to denote clinically meaningful problems in desire, arousal, orgasm, or sexual pain associated with distress and/or interpersonal difficulty, whereas sexual health/sexual well-being were used for broader constructs (e.g., satisfaction, intimacy, participation, relational functioning); sexual quality of life was reserved for outcomes explicitly framed as quality-of-life constructs rather than isolated physiological endpoints. Outcomes were extracted and interpreted by domain wherever possible, including erectile function, female sexual function, broader sexual function indices, satisfaction/distress measures, and partner/couple outcomes, alongside safety/tolerability (adverse events, discontinuations, serious harms).
Exclusion Criteria	Review type and clinical focus	Narrative reviews, expert opinions, editorials, commentaries, guidelines, and scoping reviews without systematic methods were excluded. Reviews focused exclusively on non-neurological causes of SD (e.g., cardiovascular, endocrine, oncological, primary psychiatric) were not eligible unless separate data for neurological subgroups were available. Reviews addressing only sexual education, sexual risk behaviour, or reproductive outcomes without sexual function or satisfaction outcomes were excluded.
Exclusion Criteria	Outcome and design exclusions; overlapping reviews	Reviews in which SD was not explicitly linked to a neurological disorder, or where sexual outcomes were reported only as incidental adverse events of treatments targeting the neurological condition, were excluded. Reviews restricted to cross-sectional prevalence or correlates of SD without any therapeutic intervention were ineligible. Conference abstracts without a full review, theses, and non-peer-reviewed reports were excluded. Overlapping systematic reviews were managed by forming overlap clusters defined by comparable neurological populations, the same intervention category, and the same primary outcome domain. Within each cluster, a single primary review anchored effect interpretation to minimise double-counting (Cochrane where available; otherwise the review with the highest AMSTAR 2 overall confidence and/or the most comprehensive and up-to-date coverage). For major domains, SCI-PDE5 inhibitor evidence was anchored to the network meta-analysis. MS pharmacological erectile dysfunction evidence to the Cochrane review, and stroke/ABI interventions to the Cochrane review. Other overlapping reviews were used to extract complementary outcomes and subgroup information and to assess the stability of conclusions, but pooled estimates were extracted only once per cluster. Because trial identifiers were inconsistently reported across reviews, a formal overlap metric (e.g., corrected covered area) was not calculated; overlap was treated as a potential source of indirectness and residual double-counting and reflected conservatively in certainty judgements.
PICO Evaluation	Framing of the umbrella review question	The umbrella review question was structured using PICO. Population: adults and paediatric patients with neurological disorders and SD or impaired sexual health. Intervention: pharmacological, psychological/behavioural, rehabilitation-based, or multidisciplinary treatments primarily targeting sexual function or sexual health. Comparator: placebo or sham, usual care, no treatment, waiting list, or active alternative interventions, including pre–post designs when integrated at review level. Outcomes: patient-level sexual function and satisfaction, broader sexual well-being and sexual quality of life, partner and relationship outcomes, and safety/tolerability.
Setting, Delivery & Dose	Clinical settings and intervention delivery	Primary studies were conducted in acute neurology, post-acute neurorehabilitation, outpatient neurology/rehabilitation, and community or mixed settings. During data extraction, interventions were coded by category (pharmacological, psychological, rehabilitation-based, multidisciplinary) and, where reported, described in terms of content, delivery mode (individual vs. couple, face-to-face vs. remote), provider discipline, intensity, duration, and setting, to contextualise feasibility and real-world implementation.
Data Extraction	Templates, items, and procedures	Two reviewers (AM, AC) independently extracted data using standardised, piloted templates. Extracted information included review characteristics (journal, year, country), objectives and eligibility criteria, databases and date ranges, language restrictions, protocol registration, risk-of-bias tools, and synthesis methods. Population data included diagnostic categories, age groups, sex distribution, sample sizes, disease duration, and care settings. Intervention and comparator details, sexual health outcomes (including instruments and timing), and, when available, meta-analytic estimates and safety outcomes were recorded. Data were managed in structured Excel workbooks with validation rules, range checks, and filters to ensure data integrity and traceability.
Risk of Bias & Tools	Methodological quality of reviews and primary-study risk of bias	Methodological quality of the included systematic reviews was assessed independently by two reviewers (AM, AC) using AMSTAR 2 [43]. Each domain was rated according to published guidance and used to derive an overall confidence rating. Risk-of-bias assessments of primary studies were extracted as reported, and use of tools such as Cochrane RoB 2 for randomised trials [44] and ROBINS-I for non-randomised studies [45] was recorded. The risk-of-bias profile of the underlying evidence was taken into account during synthesis.
Risk of Bias & Tools	Certainty of evidence (GRADE-based approach)	Certainty of evidence for key intervention–outcome pairings was graded using an adapted GRADE approach applied at umbrella-review level [46]. For each condition-specific pairing, a body of evidence was defined at the overlap-cluster level (anchored to the primary review and triangulated with overlapping reviews rather than additively pooled). Certainty started high when evidence was predominantly from randomised controlled trials and low when primarily from non-randomised intervention studies, and was then downgraded or upgraded across standard domains (risk of bias, inconsistency, indirectness, imprecision, publication bias). Risk of bias incorporated both primary-study appraisals reported in the reviews and review-level credibility, such that low or critically low AMSTAR 2 confidence increased the likelihood of downgrading when core review safeguards (e.g., protocol transparency, risk-of-bias assessment, synthesis methods, assessment of small-study effects) were insufficiently reported. Inconsistency was evaluated using direction and overlap of effects across reviews and heterogeneity indices when available. Indirectness captured population mixing (including reliance on the ≥70% rule when subgroup effects were unavailable), intervention heterogeneity within categories, and outcome-domain mismatch, and also accounted for unquantified overlap across reviews; extracting pooled effects once per overlap cluster and treating residual overlap uncertainty conservatively mitigated double-counting. Upgrading was considered only when consistent and precise effects were observed across multiple higher-credibility syntheses with clinically coherent estimates. Final certainty ratings (high, moderate, low, very low) were reached by consensus and used explicitly to qualify clinical conclusions and implications.
Registration & Reporting	Protocol registration and transparency	The umbrella review protocol was prospectively registered in PROSPERO (CRD420251240006), providing a prespecified methodological framework and enhancing transparency and resistance to selective reporting [42]. Any deviations from the protocol were documented and justified in the final report.
Registration & Reporting	Reporting standards	Reporting followed PRISMA 2020 guidance for systematic reviews and umbrella reviews, with detailed reporting of search methods, study selection, risk-of-bias assessment, and synthesis procedures [41]. Narrative synthesis was structured in line with SWiM (Synthesis Without Meta-analysis) guidance for overviews without de novo meta-analysis [47].
Synthesis Approach (SWiM)	Grouping and stratification of reviews	A structured narrative synthesis was adopted given expected heterogeneity in conditions, interventions, and outcome measures. Systematic reviews were first grouped by neurological diagnosis (e.g., SCI, MS, CP and other developmental conditions, PD, stroke/acquired brain injury, epilepsy, other nervous system diseases). Within each diagnostic group, reviews were organised by intervention type: pharmacological, psychological/behavioural, rehabilitation-based, and multidisciplinary or combined approaches.
Synthesis Approach (SWiM)	Direction-of-effect summaries and overlapping reviews	For each intervention type and diagnosis, findings were summarised by outcome domain (e.g., erectile function, broader sexual function, satisfaction/quality-of-life constructs, and relationship/partner outcomes), considering precision, heterogeneity, and methodological quality. Meta-analyses from the primary review within each overlap cluster were given descriptive priority and reported alongside numbers of studies and participants, without de novo pooling. Overlapping reviews were compared for methods, primary-study coverage, and conclusions; the primary review anchored effect statements to minimise double-counting, while overlapping reviews were used for triangulation and to capture unique outcomes or subgroup data. Sex-specific, gender-relevant, and couple-level findings were highlighted when available, and GRADE-based certainty ratings were integrated into the interpretation.

*Legend*: *Population*, *Intervention*, *Comparator*, *Outcome* (*PICO*); *Sexual Dysfunction* (*SD*); *Acquired Brain Injury* (*ABI*); *Spinal Cord Injury* (*SCI*); *Multiple Sclerosis* (*MS*); *Parkinson’s Disease* (*PD*); *Cerebral Palsy* (*CP*); *Sexual Quality of Life* (*SQoL*); *Quality of Life* (*QoL*); *Confidence Interval* (*CI*); *Randomized Controlled Trial* (*RCT*); *Preferred Reporting Items for Systematic Reviews and Meta-Analyses* (*PRISMA*); *Synthesis Without Meta-analysis* (*SWiM*); *International Prospective Register of Systematic Reviews* (*PROSPERO*); *A MeaSurement Tool to Assess Systematic Reviews, version* 2 (*AMSTAR* 2); *Risk of Bias* 2 (*RoB* 2); *Risk Of Bias In Non-randomized Studies of Interventions* (*ROBINS-I*); *Grading of Recommendations Assessment*, *Development and Evaluation* (*GRADE*).

**Table 2:** Condition-specific summary of intervention evidence, outcome domains, and umbrella-level certainty.

Condition	Intervention Category (Examples)	Primary Review Used for Synthesis (AMSTAR 2 Confidence)	Outcome Domain(s) Assessed	Main Outcome Direction (Umbrella Synthesis)	Umbrella-Level Certainty (GRADE)	Key Notes (Population Focus, HARMS, and Evidence Gaps)
SCI	Pharmacological (PDE5 inhibitors; sildenafil, tadalafil, vardenafil) [48,49,54,55,56,64,68]	Network meta-analysis [56] (moderate–high)	Erectile function; intercourse success; patient satisfaction	Consistent short-term improvement in erectile outcomes and intercourse success versus placebo	Moderate (probably beneficial for ED in adult men with SCI)	Evidence mainly from adult men with chronic SCI; follow-up typically weeks and rarely beyond 3–6 months; partner/relationship outcomes rarely measured; adverse events typically mild (headache, flushing, dyspepsia) [48,49,54,55,56,64,68]
SCI	Devices/procedures (intracavernosal injections, vacuum devices, penile prostheses) [48,49,64,67,68,69]	Neurogenic SD review/meta-synthesis [64] (low–moderate) and broader overviews [48,49,67,68,69] (often low/critically low)	Erection sufficient for intercourse; technical success; selected satisfaction measures; complications	High technical success rates reported, but patient-centred outcomes inconsistently assessed and comparators often absent	Low to very low (hypothesis-generating; likely benefit for erection, uncertain for broader sexual outcomes)	Predominantly case series/nonrandomized studies with selection bias and incomplete harms reporting; common complications include pain, hematoma/fibrosis, priapism (injections) and infection/mechanical failure (prostheses) [64,67,68,69]
SCI	Neuromodulation/stimulation (penile vibratory stimulation, electroejaculation, sacral/perineal stimulation) [48,64,69,72]	Neurogenic SD review [72] (low) and broader SCI reviews [48,64,69] (low/critically low)	Ejaculatory function; combined bowel/bladder/sexual endpoints; limited satisfaction/QoL	Signals of benefit for ejaculation in selected men; broader sexual function effects unclear	Very low (uncertain effectiveness beyond selected physiological endpoints)	Small heterogeneous studies; outcomes often not aligned with multidimensional sexual health; applicability mainly to selected male fertility/ejaculation goals [69,72]
SCI	Hormonal/adjunct (testosterone ± exercise/FES) [64,65]	SCI/TBI-focused synthesis [64] (low–moderate) and adjunct trials summary [65] (low)	Libido and erectile outcomes (often secondary); global sexual function scales	Inconsistent changes in sexual outcomes; no stable pattern across validated measures	Very low (uncertain benefit for sexual outcomes)	Interventions primarily targeted metabolic/musculoskeletal outcomes; sexual measures secondary and underpowered; generalizability limited [64,65]
MS	Pharmacological (PDE5 inhibitors) [48,49,51,52,53,57]	Cochrane review [53] (high)	Erectile function and intercourse success (men); limited broader domains	Improves erectile function and intercourse success versus placebo in men	Moderate (probably beneficial for ED in men with MS)	Overall sample sizes modest and follow-up short; evidence sparse for sexual satisfaction, relationship outcomes, and female SD (these remain uncertain) [51,53,57,58,59,73]
MS	Physiotherapy (pelvic floor muscle training ± biofeedback/electrostimulation) [58,70]	Meta-analyses [58,70] (moderate/low)	Female sexual function indices; urinary symptoms; health-related QoL	Improvement reported in several trials, particularly among women; co-benefits on urinary symptoms common	Low to moderate (probably beneficial in selected adults; certainty limited by trial quality)	Heterogeneous protocols and risk-of-bias concerns (concealment/blinding/incomplete data); adverse events uncommon and usually mild/transient [58,70]
MS	Psychobehavioral (CBT, psychoeducation, PLISSIT-based programs; individual/couples counselling) [52,57,58,59,73]	Psychosexual intervention reviews [52,59,73] (often low/critically low)	Sexual satisfaction; sexual distress; communication; occasional partner outcomes	Potential improvements in satisfaction/distress in some trials; effects variable and difficult to compare	Low (may be beneficial; evidence imprecise and heterogeneous)	Mostly small single-centre trials with performance/detection bias; partner outcomes inconsistently measured; durability beyond a few months uncertain [52,59,73]
MS	Multidisciplinary/pathways (structured sexual rehabilitation within MS care) [52,57,58,59,70,73]	Mixed-intervention synthesis [57] (low–moderate) and narrative reviews [51,52,73] (low)	Broad sexual function; satisfaction; participation/QoL; limited relationship outcomes	Promising signals, but effects cannot be attributed to specific components and estimates are imprecise	Low to very low (hypothesis-generating)	Complex packages vary in content/intensity; limited standardization and short follow-up; women and couples underrepresented [51,57,70,73]
Stroke/ABI	Psychobehavioral/education (sexual rehabilitation, counselling, communication training; occasional couples sessions) [60,61,62]	Cochrane review [50] (high) anchored; supplemented by later reviews [60,61,62] (low)	Sexual knowledge; readiness to resume activity; satisfaction; coital frequency; limited domain granularity	Short-term improvements reported in some studies for satisfaction/readiness and knowledge; domain-specific effects inconsistent	Low (may be beneficial; limited and heterogeneous trials)	Trials small and often quasi-experimental; partner/couple outcomes underpowered; long-term maintenance uncertain [60,61,62]
Stroke/ABI	Pharmacological (sildenafil for ED; sertraline for PE after stroke) [48,50,60]	Cochrane review [50] (high)	Erectile function or ejaculation latency; satisfaction variably reported	Possible improvement in selected physiological outcomes, but estimates imprecise and not consistently replicated	Low to very low (uncertain effectiveness; sparse trials)	Evidence based on few small studies; adverse event data limited for rare harms; broader sexual well-being outcomes seldom assessed [50,60]
Stroke/ABI	ABI beyond stroke (TBI/other ABI-specific sexual rehabilitation) [48,60,61,62]	No dedicated high-credibility intervention review; evidence mainly narrative [48,60,61,62]	Intimacy, mood, relationship functioning; variable sexual function measures	Insufficient review-level intervention evidence to draw supported conclusions	Very low (evidence gap)	ABI populations often merged with stroke; dedicated intervention trials scarce; recommendations largely extrapolated [48,60,61,62]
PD	Procedural (deep brain stimulation cohorts) [63,71]	DBS-focused reviews [63,71] (low/critically low)	Erectile function; sexual satisfaction (usually secondary endpoints)	Mixed or no clear benefit; findings confounded by disease severity and medication changes	Very low (uncertain effect on sexual outcomes)	Sexual outcomes rarely primary; limited control groups; interpretation limited by confounding and imprecision [63,71]
PD	Pharmacological/rehabilitation approaches [48,49,50,51,52,53,57,60,63,71]	Evidence dispersed across mixed-condition reviews [48,49,60] (low) and PD-focused syntheses [63,71] (low)	Multi-domain sexual function (desire/arousal/orgasm) infrequently measured	No coherent intervention evidence base at review level	Very low (evidence gap for tested interventions)	Clinical management often relies on assessment of medication effects and comorbid factors rather than supported interventional trials [63,71]
Epilepsy	Medication-related management (switching/selection of antiseizure medications) [66]	Medication-focused review [66] (low)	Sex hormones/testosterone; erectile function; libido (often observational)	Associations suggest drug-related contributors; intervention effects from switching strategies not established	Low to very low (uncertain benefit of medication changes for sexual outcomes)	Randomized switching trials largely absent; outcome measurement inconsistent; evidence mainly associative [66]
Epilepsy	Psychosexual/rehabilitation interventions [58,59,60,69,72,73]	Mixed-condition reviews [58,59,60,72,73] (low/critically low)	Sexual well-being; relationship outcomes; distress	No eligible synthesised intervention effects; evidence largely absent	Very low (evidence gap)	Interventional research scarce; future work needed with standardized outcomes and inclusion of partners [58,59,60,72,73]
CP/Neuromuscular	Across categories (sexual counselling/education within rehabilitation; extrapolated approaches) [48,49,58,59,60,69,72,73]	No robust condition-specific intervention review; evidence mainly descriptive [48,49,58,59,60,69,72,73]	Needs/prevalence; sexual participation; relationship issues (treatment effects seldom evaluated)	No supported interventional conclusions at review level	Very low (evidence gap across conditions and ages)	Pediatric/adolescent intervention reviews largely absent, including pediatric CP; data mainly describe barriers and unmet needs rather than treatment effectiveness [48,49,58,59,60,69,72,73]

*Legend*: *ABI*, *acquired brain injury*; *CBT*, *cognitive behavioural therapy*; *CP*, *cerebral palsy*; *DBS*, *deep brain stimulation*; *ED*, *erectile dysfunction*; *FES*, *functional electrical stimulation*; *GRADE*, *Grading of Recommendations*, *Assessment*, *Development and Evaluation*; *IIEF*, *International Index of Erectile Function*; *MS*, *multiple sclerosis*; *NMA*, *network meta-analysis*; *PD*, *Parkinson’s disease*; *PDE*5, *phosphodiesterase type* 5; *PDE*5 *inhibitor*, *phosphodiesterase type* 5 *inhibitor*; *PE*, *premature ejaculation*; *PLISSIT*, *Permission*, *Limited Information*, *Specific Suggestions*, *Intensive Therapy*; *QoL*, *quality of life*; *SCI*, *spinal cord injury*; *SD*, *sexual dysfunction*; *TBI*, *traumatic brain injury*.

## Data Availability

No new data were created or analyzed in this study.

## Supplementary Materials

The following supporting information can be downloaded at: https://www.mdpi.com/article/10.3390/medsci14010037/s1, Supplementary Table S1: Summary of the included systematic reviews (neurological condition, population, interventions, comparators, outcomes, and key findings). Supplementary Table S2: AMSTAR 2 risk-of-bias assessment for all systematic reviews included in the umbrella review on sexual health after neurological disorders.

## Abbreviations

**Acronym**	**Full term**
ABI	Acquired brain injury
AMSTAR 2	A MeaSurement Tool to Assess systematic Reviews, version 2
CC BY	Creative Commons Attribution license
CI	Confidence interval
CP	Cerebral palsy
DALYs	Disability-adjusted life years
GRADE	Grading of Recommendations Assessment, Development and Evaluation
MEDLINE	Medical Literature Analysis and Retrieval System Online
MeSH	Medical Subject Headings
MS	Multiple sclerosis
PD	Parkinson’s disease
PDE5	Phosphodiesterase type 5
PICO	Population, intervention, comparator, outcome
PLISSIT	Permission, Limited Information, Specific Suggestions, Intensive Therapy
PRISMA	Preferred Reporting Items for Systematic Reviews and Meta-Analyses
PROSPERO	International Prospective Register of Systematic Reviews
QoL	Quality of life
RCT	Randomized controlled trial
RoB	Risk of bias
RoB 2	Risk of Bias 2 (revised Cochrane risk-of-bias tool for randomized trials)
ROBINS-I	Risk Of Bias In Non-randomized Studies of Interventions
RSC	Rocco Salvatore Calabrò
SCI	Spinal cord injury
Scopus	Scopus database
SD	Sexual dysfunction
SQoL	Sexual quality of life
SWiM	Synthesis Without Meta-analysis
TBI	Traumatic brain injury
WHO	World Health Organization
