# Supplementary material for: Sexual Health After Neurological Disorders: A Comprehensive Umbrella Review of Treatment Evidence
Source: Med Sci (Basel). 2026 Jan 10;14(1):37. doi: 10.3390/medsci14010037 (PMC12821412; doi:10.3390/medsci14010037)
Supplement: Supplementary file 1 [file medsci-14-00037-s001.zip › Supplementary Table 1.pdf]

Supplementary Table 1: AMSTAR 2 Risk of Bias Assessment – Umbrella Review on Sexual Health After Neurological Disorders

Study (Author, year)	Items 1–2: PICO; protocol/registration	Items 3–4: Design justification; search	Items 5–6: Selection; data extraction	Items 7–8: Excluded studies; description of included	Items 9–10: RoB of primary studies; funding of primary studies	Items 11–12: MA methods; RoB impact on synthesis	Items 13–14: RoB in interpretation; heterogeneity	Items 15–16: Publication bias; review funding/COI	Overall A2 rating (confidence in results)
Lombardi et al., 2015 [48]	**Item 1:** Y (clearly formulated PICO for CNS-related SD).<br><br>**Item 2:** N (no protocol/registration reported).	**Item 3:** PY (study designs mentioned but not explicitly justified).<br><br>**Item 4:** PY (search in 2 DBs + references; limited languages).	**Item 5:** Y (study selection by ≥2 reviewers).<br><br>**Item 6:** Y (data extraction by multiple independent reviewers).	**Item 7:** N (no table of excluded studies with reasons).<br><br>**Item 8:** Y (adequate description of included studies).	**Item 9:** N (no formal RoB/quality assessment of primary studies).<br><br>**Item 10:** N (funding of primary studies not systematically reported).	**Item 11:** NA (no MA undertaken).<br><br>**Item 12:** NA (not applicable without MA).	**Item 13:** N (RoB of primary studies not systematically considered in discussion).<br><br>**Item 14:** NA (no statistical heterogeneity to explain).	**Item 15:** N (no assessment of publication bias or small-study effects).<br><br>**Item 16:** Y (review funding/COI clearly reported).	Critically low – ≥3 critical flaws (items 2, 7, 9, 15) despite otherwise acceptable reporting.
Del Popolo et al., 2020 [49]	**Item 1:** Y (explicit PICO for NSD treatments).<br><br>**Item 2:** N (no protocol registration).	**Item 3:** PY (eligible designs defined but rationale not fully justified).<br><br>**Item 4:** Y (multi-database search with strategy described; English-only).	**Item 5:** Y (dual independent screening).<br><br>**Item 6:** Y (dual independent data extraction).	**Item 7:** N (no detailed list of excluded full texts with reasons).<br><br>**Item 8:** Y (characteristics of included studies summarised).	**Item 9:** N (no structured RoB/quality appraisal reported).<br><br>**Item 10:** N (funding of primary studies rarely documented).	**Item 11:** NA (purely narrative synthesis).<br><br>**Item 12:** NA.	**Item 13:** PY (general discussion of study limitations without formal RoB integration).<br><br>**Item 14:** NA (no MA/heterogeneity statistics).	**Item 15:** N (no assessment of publication bias).<br><br>**Item 16:** Y (review funding/COI declared).	Critically low – multiple critical weaknesses (items 2, 7, 9, 15) in otherwise PRISMA-aligned SR.
Stratton et al., 2020 [50]	**Item 1:** Y (clear PICO on post-stroke SD interventions).<br><br>**Item 2:** Y (Cochrane protocol published a priori).	**Item 3:** Y (restriction to RCTs explicitly justified by objectives).<br><br>**Item 4:** Y (extensive multi-database + trials-register search with full strategies).	**Item 5:** Y (two authors independently screened studies).<br><br>**Item 6:** Y (independent duplicate data extraction).	**Item 7:** Y (table of excluded studies with reasons).<br><br>**Item 8:** Y (detailed characteristics of included RCTs).	**Item 9:** Y (Cochrane RoB tool applied to each trial).<br><br>**Item 10:** PY (funding of primary studies reported but not fully tabulated).	**Item 11:** Y (standard Cochrane MA methods; limited pooling due to heterogeneity).<br><br>**Item 12:** Y (RoB and GRADE explicitly used to interpret pooled and narrative findings).	**Item 13:** Y (RoB and trial quality central to discussion and conclusions).<br><br>**Item 14:** Y (sources of heterogeneity explored qualitatively; planned subgroup/sensitivity analyses).	**Item 15:** NA/PY (formal publication-bias tests not feasible with 3 RCTs but issue acknowledged).<br><br>**Item 16:** Y (review funding/COI transparently reported).	Moderate – generally robust Cochrane methods with only minor non-critical limitations (items 10 and 15).
Giannopapas et al., 2023 [51]	**Item 1:** Y (focused PICO on SD therapies in PwMS).<br><br>**Item 2:** N (no protocol/registration).	**Item 3:** N (no explicit justification of included study designs).<br><br>**Item 4:** N (single-database search in PubMed plus reference checking).	**Item 5:** Y (two reviewers screened records).<br><br>**Item 6:** Y (two reviewers extracted data using a standard form).	**Item 7:** N (no list of excluded studies with reasons).<br><br>**Item 8:** Y (basic descriptive summary of each included study).	**Item 9:** PY (informal quality grading using Marrie–Wolfson criteria but no structured RoB domains).<br><br>**Item 10:** N (funding of primary studies rarely reported).	**Item 11:** NA (no MA).<br><br>**Item 12:** NA.	**Item 13:** PY (narrative comments on methodological weaknesses without systematic RoB weighting).<br><br>**Item 14:** NA (no statistical heterogeneity analysis).	**Item 15:** N (no consideration of publication bias).<br><br>**Item 16:** Y (review funding/COI stated).	Critically low – several critical flaws (items 2, 4, 7, 15) and only partial attention to RoB (item 9).
Pöttgen et al., 2020 [52]	**Item 1:** Y (clear PICO for psychobehavioural SD interventions in MS).<br><br>**Item 2:** N (no protocol registration).	**Item 3:** N (study designs eligible but not explicitly justified).<br><br>**Item 4:** Y (multi-database search with defined strategy and criteria).	**Item 5:** Y (two of four reviewers independently selected studies).<br><br>**Item 6:** Y (data extraction in duplicate with consensus).	**Item 7:** N (no detailed excluded-studies table).<br><br>**Item 8:** Y (adequate tabular description of included trials).	**Item 9:** Y (CASP tools used to appraise primary-study quality).<br><br>**Item 10:** N (funding sources of individual studies not systematically reported).	**Item 11:** NA (no quantitative MA conducted).<br><br>**Item 12:** NA.	**Item 13:** Y (study quality explicitly considered in narrative synthesis and conclusions).<br><br>**Item 14:** NA (no heterogeneity statistics).	**Item 15:** N (no formal publication-bias assessment).<br><br>**Item 16:** Y (review funding/COI disclosed).	Critically low – at least two critical weaknesses (items 2, 7, 15) despite appropriate use of quality appraisal tools.

García-Perdomo et al., 2016 [55]	**Item 1:** Y (clearly defined PICO for PDE5Is vs placebo in SCI-related ED).<br><br>**Item 2:** Y (protocol registered prospectively in PROSPERO: CRD42014010477).	**Item 3:** Y (restriction to RCTs justified to obtain high-level evidence).<br><br>**Item 4:** Y (comprehensive multi-database search without language or date limits, strategies reported).	**Item 5:** Y (two investigators independently and blindly screened records).<br><br>**Item 6:** Y (standardised data extraction by ≥2 reviewers with cross-checking).	**Item 7:** N (no separate table of excluded studies with reasons, only PRISMA flow).<br><br>**Item 8:** Y (detailed description of each trial, including design, participants and outcomes).	**Item 9:** Y (Cochrane RoB tool applied to all included trials).<br><br>**Item 10:** N (funding and COI of primary studies not systematically reported).	**Item 11:** Y (random-effects MA with appropriate effect measures and heterogeneity assessment).<br><br>**Item 12:** Y (RoB, heterogeneity and trial quality considered when interpreting pooled effects).	**Item 13:** Y (limitations of evidence, including RoB and imprecision, explicitly discussed).<br><br>**Item 14:** Y (sources of statistical heterogeneity explored via subgroup/sensitivity analyses).	**Item 15:** PY (formal publication-bias tests limited by few studies, but small-study effects discussed).<br><br>**Item 16:** Y (review funding/COI clearly declared).	Low – one critical weakness (item 7) and minor concerns about reporting of primary-study funding.
Jia et al., 2016 [54]	**Item 1:** Y (explicit PICO for PDE5Is vs placebo in SCI-related ED).<br><br>**Item 2:** N (no evidence of protocol registration).	**Item 3:** Y (restriction to double-blind placebo-controlled RCTs justified by objective).<br><br>**Item 4:** Y (search of MEDLINE, EMBASE and Cochrane Library without language limits, plus hand-searching).	**Item 5:** Y (two reviewers independently screened titles/abstracts and full texts).<br><br>**Item 6:** Y (duplicate data extraction with predefined forms).	**Item 7:** N (no detailed list of excluded studies with reasons).<br><br>**Item 8:** Y (baseline characteristics and design features of all six RCTs tabulated).	**Item 9:** Y (Cochrane RoB domains assessed and trials graded A–C for quality).<br><br>**Item 10:** N (funding/COI of individual trials not systematically summarised).	**Item 11:** Y (appropriate fixed/random-effects MA based on heterogeneity; subgroup analyses by molecule).<br><br>**Item 12:** Y (RoB and trial quality considered when interpreting pooled outcomes).	**Item 13:** Y (discussion explicitly links limitations of trials and RoB to strength of conclusions).<br><br>**Item 14:** Y (heterogeneity quantified with I^2 and explored via sensitivity analyses).	**Item 15:** N (no formal publication-bias assessment such as funnel plots reported).<br><br>**Item 16:** Y (review funding/COI statement provided).	Low – at least one critical flaw (items 2, 7, 15) but otherwise methodologically robust meta-analysis.
Tienforti et al., 2025 [56]	**Item 1:** Y (clear PICO comparing individual PDE5Is for SCI-related ED).<br><br>**Item 2:** Y (NMA protocol prospectively registered in PROSPERO: CRD42023433166).	**Item 3:** Y (restriction to RCTs justified; inclusion/exclusion criteria by design clearly stated).<br><br>**Item 4:** Y (multi-database search with explicit strategy and PRISMA-NMA reporting).	**Item 5:** Y (two reviewers independently selected studies with third-party arbitration).<br><br>**Item 6:** Y (duplicate data extraction using piloted forms).	**Item 7:** N (no tabular listing of excluded studies with reasons, only flow diagram).<br><br>**Item 8:** Y (comprehensive tables describing RCT characteristics, interventions and outcomes).	**Item 9:** Y (Cochrane RoB assessment applied to all RCTs with aggregated summary).<br><br>**Item 10:** PY (trial funding/COI partly reported in text but not fully tabulated).	**Item 11:** Y (appropriate pairwise MA and frequentist NMA with assessment of transitivity and consistency).<br><br>**Item 12:** Y (RoB and study quality incorporated into interpretation of both MA and NMA rankings).	**Item 13:** Y (limitations of evidence base, including RoB and indirectness, explicitly discussed).<br><br>**Item 14:** Y (heterogeneity and inconsistency evaluated using network metrics and node-splitting).	**Item 15:** PY (small-study effects and publication bias considered qualitatively; formal tests limited by small network).<br><br>**Item 16:** Y (funding sources and COI for the review clearly declared).	High/Moderate – no critical flaws; minor concerns relate to incomplete reporting of primary-study funding and limited publication-bias testing.
Xiao et al., 2012 [53]	**Item 1:** Y (focused PICO on sildenafil vs placebo for ED in MS).<br><br>**Item 2:** Y (Cochrane protocol developed a priori).	**Item 3:** Y (restriction to RCTs justified; study designs clearly prespecified).<br><br>**Item 4:** Y (highly sensitive multi-database and trials-register search without language restrictions).	**Item 5:** Y (two authors independently selected studies).<br><br>**Item 6:** Y (independent duplicate data extraction with consensus procedures).	**Item 7:** Y (Cochrane 'Characteristics of excluded studies' table with reasons for exclusion).<br><br>**Item 8:** Y (detailed tables of trial characteristics and outcome measures).	**Item 9:** Y (Cochrane RoB tool used for both sequence generation and other domains).<br><br>**Item 10:** PY (funding/COI of primary studies reported but not systematically summarised).	**Item 11:** Y (fixed-effect MA with appropriate effect measures for SEP and GAQ outcomes).<br><br>**Item 12:** Y (GRADE used to rate certainty; RoB explicitly incorporated in Summary of Findings).	**Item 13:** Y (interpretation of results explicitly conditioned on RoB, imprecision and indirectness).<br><br>**Item 14:** Y (heterogeneity assessed and discussed, though pooling limited by few trials).	**Item 15:** NA/PY (formal publication-bias testing not feasible with two trials, but limitation acknowledged).<br><br>**Item 16:** Y (Cochrane review funding and author COI transparently reported).	Moderate – strong Cochrane methods with only minor non-critical limitations (items 10 and 15).

Afshar et al., 2022 [57]	**Item 1:** Y (PICO on interventions for sexual function/SD in individuals with MS).<br><br>**Item 2:** N (no protocol registration reported).	**Item 3:** PY (broad inclusion of interventional designs described but not fully justified).<br><br>**Item 4:** Y (extensive multi-database search with keywords/blocks reported and no language limits beyond abstract).	**Item 5:** PY (selection process described, but dual independent screening for all stages not clearly stated).<br><br>**Item 6:** Y (data extraction conducted independently by two authors).	**Item 7:** N (no table of excluded studies with reasons).<br><br>**Item 8:** Y (two structured tables summarising key characteristics of sexual function and SD studies).	**Item 9:** Y (custom quality checklist plus Cochrane RoB domains applied to included trials).<br><br>**Item 10:** N (funding/COI of individual primary studies not systematically reported).	**Item 11:** Y (RevMan-based MA for homogeneous subsets with appropriate models and heterogeneity statistics).<br><br>**Item 12:** PY (study quality considered in narrative synthesis, but not consistently integrated into pooled estimates).	**Item 13:** PY (limitations related to RoB, heterogeneity and reporting discussed in general terms).<br><br>**Item 14:** Y (statistical heterogeneity assessed using Chi² and I²; some exploration in subgroup analyses).	**Item 15:** N (no formal publication-bias assessment reported).<br><br>**Item 16:** Y (review funding and author COI disclosed).	Critically low – several critical flaws (items 2, 5, 7, 10, 15) despite reasonably thorough search and RoB assessment.
Gopal et al., 2021 [58]	**Item 1:** Y (clearly defined PICO for PT-scope interventions targeting sexual dysfunction in adults with MS).<br><br>**Item 2:** N (no prospective protocol registration reported).	**Item 3:** Y (predefined inclusion of level-II evidence or higher, with study designs specified a priori).<br><br>**Item 4:** Y (multi-database search of PubMed, CINAHL and PEDro with explicit terms and a PRISMA flow diagram).	**Item 5:** Y (study selection performed independently by two reviewers with consensus procedures).<br><br>**Item 6:** Y (data extraction undertaken in duplicate with agreement checking).	**Item 7:** N (no structured list of excluded studies with reasons).<br><br>**Item 8:** Y (included studies described in adequate detail, including MS characteristics, PT modalities, comparators and outcomes).	**Item 9:** Y (risk of bias of individual trials and cohort studies assessed with PEDro and STROBE checklists and summarised).<br><br>**Item 10:** N (funding sources of primary studies not systematically reported).	**Item 11:** Y (appropriate fixed/random-effects meta-analyses using Cohen's d with Q and I² statistics for heterogeneity).<br><br>**Item 12:** PY (study quality and RoB considered narratively but not explicitly used to weight or stratify pooled estimates).	**Item 13:** Y (interpretation explicitly considers small sample sizes, design limitations and overall RoB when formulating conclusions).<br><br>**Item 14:** Y (between-study heterogeneity quantified and used to justify model choice).	**Item 15:** N (no formal assessment of publication bias or small-study effects).<br><br>**Item 16:** Y (review funding/COI clearly reported).	Low – critical weaknesses in items 2, 7, 10 and 15, although otherwise sound methods and transparent MA support cautious confidence in the findings.
Auger et al., 2021 [60]	**Item 1:** Y (clearly formulated PICO question on allied health professional–delivered post-stroke sexual rehabilitation).<br><br>**Item 2:** N (no prospectively registered protocol or protocol reference).	**Item 3:** Y (explicit rationale for including diverse quantitative and qualitative designs in an emerging field).<br><br>**Item 4:** Y (multi-database search in several databases plus reference and citation tracking, with dates and strategy reported).	**Item 5:** Y (titles/abstracts and full texts screened independently by two reviewers with consensus procedures).<br><br>**Item 6:** PY (data extraction described inconsistently; main text suggests single extractor using piloted forms).	**Item 7:** N (no table of excluded full-text studies with individual reasons; only aggregated PRISMA counts).<br><br>**Item 8:** Y (detailed tabular and narrative description of populations, interventions and outcomes).	**Item 9:** PY (study quality considered via design-based evidence levels rather than a full domain-level RoB tool).<br><br>**Item 10:** N (funding of individual primary studies not systematically extracted or reported).	**Item 11:** NA (no statistical MA performed).<br><br>**Item 12:** NA (not applicable in the absence of MA).	**Item 13:** PY (limitations of the evidence base discussed qualitatively but not consistently linked to specific outcomes).<br><br>**Item 14:** NA (no heterogeneity analysis required without MA).	**Item 15:** N (no formal assessment of publication bias or small-study effects).<br><br>**Item 16:** Y (review-level funding and COI statements clearly reported).	Critically low – multiple critical weaknesses (items 2, 7, 15) and only partial consideration of RoB and study quality.
Brandão et al., 2025 [61]	**Item 1:** Y (explicit PICO-style question on psychological interventions to improve post-stroke sexual health).<br><br>**Item 2:** Y (protocol prospectively registered in PROSPERO).	**Item 3:** Y (clear justification for including both quantitative and qualitative designs to capture complex sexual outcomes).<br><br>**Item 4:** Y (multi-database search with transparent strategies and dates, supplemented by reference screening).	**Item 5:** N (study selection undertaken primarily by a single reviewer with consultation, not independent duplicate screening).<br><br>**Item 6:** N (data extraction conducted by one reviewer without explicit duplicate verification).	**Item 7:** N (no full list of excluded full-text studies with reasons beyond PRISMA flow counts).<br><br>**Item 8:** Y (comprehensive description of included studies and interventions in text and tables).	**Item 9:** Y (RoB assessed using appropriate tools matched to study design with domain-level judgements reported).<br><br>**Item 10:** N (funding or sponsorship of primary studies not systematically extracted or tabulated).	**Item 11:** NA (no quantitative MA was undertaken).<br><br>**Item 12:** NA (not applicable without MA).	**Item 13:** Y (RoB and methodological limitations explicitly integrated into the narrative synthesis and conclusions).<br><br>**Item 14:** NA (no statistical heterogeneity to explain).	**Item 15:** N (no formal assessment of publication bias or small-study effects).<br><br>**Item 16:** Y (review funding and COI declarations clearly reported).	Critically low – two critical weaknesses (items 7 and 15) plus non-critical limitations in selection and extraction methods.

Esteve-Ríos et al., 2020 [59]	**Item 1:** Y (well-framed PICO question on interventions to improve sexuality in women with MS).<br><br>**Item 2:** N (no prospectively registered protocol reported).	**Item 3:** PY (restriction to RCTs and quasi-RCTs is reasonable but not explicitly justified in relation to the review question).<br><br>**Item 4:** Y (comprehensive search across multiple databases and grey literature with strategies and dates reported).	**Item 5:** Y (titles/abstracts and full texts screened by two independent reviewers with arbitration for disagreements).<br><br>**Item 6:** Y (data extraction performed in duplicate using piloted forms).	**Item 7:** N (no structured list of excluded full-text studies with individual reasons for exclusion).<br><br>**Item 8:** Y (detailed tabular description of participant characteristics, interventions and outcomes).	**Item 9:** Y (methodological quality of each trial appraised using a validated scale and summarised).<br><br>**Item 10:** N (funding of primary studies rarely reported and not systematically extracted).	**Item 11:** NA (no MA conducted because of heterogeneity of interventions and outcomes).<br><br>**Item 12:** NA (not applicable in the absence of MA).	**Item 13:** Y (trial quality and risk of bias explicitly considered when interpreting patterns of effect).<br><br>**Item 14:** NA (no statistical heterogeneity assessment required).	**Item 15:** N (no formal assessment of publication bias or small-study effects).<br><br>**Item 16:** Y (review funding and COI statements clearly reported).	Critically low – ≥2 critical flaws (items 2, 7, 15) despite otherwise rigorous search, selection and quality assessment.
Bahadori et al., 2024 [63]	**Item 1:** Y (clearly defined PICO for adults with PD treated with STN-DBS and assessed for sexual function and QoL).<br><br>**Item 2:** N (no explicit mention of a pre-registered protocol or PROSPERO/other registration).	**Item 3:** PY (observational cohort and case–control designs are appropriate for surgical exposures, but the rationale for excluding trials or other designs is only briefly implied).<br><br>**Item 4:** Y (multi-database search to June 2024 with transparent search strategy and PRISMA-style reporting).	**Item 5:** PY (study selection procedures described, but independence/duplication of screening is not clearly stated).<br><br>**Item 6:** PY (data extraction performed by multiple reviewers with consensus, although explicit independent duplicate extraction is not fully detailed).	**Item 7:** N (no full table of excluded studies with individual reasons beyond aggregate counts in the flow-diagram).<br><br>**Item 8:** Y (key characteristics of included cohorts—setting, sample size, DBS target, follow-up and outcomes—are adequately reported).	**Item 9:** Y (risk of bias of observational studies assessed with ROBINS-I and summarised across domains).<br><br>**Item 10:** N (funding sources for individual cohorts are not systematically extracted or incorporated into the synthesis).	**Item 11:** Y (random-effects MA with appropriate effect measures, heterogeneity statistics and sensitivity analyses).<br><br>**Item 12:** PY (ROBINS-I assessments are acknowledged in grading and interpretation of results but not consistently used to weight analyses or conduct RoB-based sensitivity analyses).	**Item 13:** Y (authors explicitly discuss major limitations of the evidence, including observational design, residual confounding and selective reporting).<br><br>**Item 14:** Y (clinical and statistical heterogeneity are evaluated using I^2 and subgroup analyses, and their implications for interpretation are discussed).	**Item 15:** Y (small-study effects/publication bias explored with funnel plots and Egger/Begg tests where data allowed).<br><br>**Item 16:** Y (review-level funding and COI clearly reported; authors state no external funding and no conflicts of interest).	Critically low – at least two critical weaknesses (items 2 and 7) and additional concerns about incomplete reporting of reviewer processes and primary-study funding, despite otherwise robust contemporary methods.
DeForge et al., 2006 [64]	**Item 1:** Y (well-defined PICO focused on sexuality and ED in males with SCI and effectiveness of available interventions).<br><br>**Item 2:** N (no explicit statement of an a priori protocol or formal registration, although the work was commissioned as an evidence report).	**Item 3:** Y (inclusion of RCTs, observational studies and case series is justified by the broad therapeutic question and scarcity of high-quality trials).<br><br>**Item 4:** Y (comprehensive search of six databases, conference proceedings and industry submissions over several decades with clearly described strategies).	**Item 5:** Y (titles/abstracts and full texts screened in duplicate by pairs of reviewers with arbitration of disagreements).<br><br>**Item 6:** Y (data abstraction performed in duplicate by two of four trained reviewers using piloted forms and consensus procedures).	**Item 7:** PY (reasons for exclusion documented in the full AHRQ evidence report but not presented as a complete study-level table in the journal summary).<br><br>**Item 8:** Y (detailed tables describe populations, interventions, comparators and outcomes for RCTs, observational cohorts and case series).	**Item 9:** Y (methodological quality of RCTs, observational studies and case series assessed using Jadad, Newcastle–Ottawa and a 19-item checklist, respectively).<br><br>**Item 10:** N (funding sources of primary studies are not systematically collected or considered in the synthesis).	**Item 11:** Y (random-effects pooling of response proportions for key interventions such as ICIs and sildenafil with appropriate variance estimation).<br><br>**Item 12:** PY (quality assessments are summarised and design differences considered, but no formal RoB-stratified or sensitivity analyses are conducted).	**Item 13:** Y (limitations related to predominance of case series, short follow-up and generalisability are explicitly discussed when drawing conclusions).<br><br>**Item 14:** PY (clinical heterogeneity is acknowledged; statistical heterogeneity is not consistently quantified or explored beyond descriptive comparison).	**Item 15:** N (no formal assessment of publication bias or small-study effects, despite several pooled analyses of uncontrolled series).<br><br>**Item 16:** Y (review-level sponsorship by AHRQ and absence of commercial conflicts are clearly reported).	Critically low – multiple critical weaknesses (items 2, 7 and 15) and limited consideration of primary-study funding, although search, selection, data abstraction and quality appraisal procedures are otherwise rigorous for its era.

Dusenbury et al., 2017 [62]	**Item 1:** Y (clearly framed question on determinants of sexual function and effectiveness of sexual rehabilitation after stroke).<br><br>**Item 2:** N (no evidence of a prospectively registered or published protocol).	**Item 3:** PY (inclusion of quantitative observational and interventional designs is reasonable for the topic, but justification of eligible designs is brief).<br><br>**Item 4:** Y (systematic search of multiple databases from 2000 to 2016 with reported key terms and limits).	**Item 5:** PY (screening and eligibility assessment described, but independence and duplication of study selection are not explicitly stated).<br><br>**Item 6:** Y (two primary reviewers independently extracted data with involvement of a third author to resolve discrepancies).	**Item 7:** N (no complete table of excluded full-text articles with reasons; only aggregated numbers are presented in the flow diagram).<br><br>**Item 8:** Y (included studies are described with respect to design, sample, determinants/interventions and key outcomes).	**Item 9:** Y (study quality appraised using the EPHPP tool, with ratings reported and used to characterise the evidence base).<br><br>**Item 10:** N (funding sources and potential conflicts for primary studies are not systematically extracted or discussed).	**Item 11:** NA (no quantitative MA conducted because of heterogeneity in designs and outcomes).<br><br>**Item 12:** NA (not applicable in the absence of MA).	**Item 13:** Y (limitations, including predominance of weak observational designs, small samples and measurement issues, are discussed in depth when formulating conclusions).<br><br>**Item 14:** NA (no pooled effect estimates; statistical heterogeneity across studies is not formally assessed).	**Item 15:** N (publication bias/small-study effects are not formally assessed or discussed, even qualitatively).<br><br>**Item 16:** N (no explicit statement on review-level funding sources or authors' conflicts of interest).	Critically low – at least two critical weaknesses (items 2, 7 and 15) together with incomplete reporting of selection procedures and primary-study funding, despite appropriate use of a validated quality-assessment tool.
McLoughlin et al., 2023 [65]	**Item 1:** Y (well-defined PICO focused on testosterone therapy in adult men with SCI or TBI and its effects on body composition and functional outcomes).<br><br>**Item 2:** N (no prospectively registered protocol or formal registration is reported).	**Item 3:** PY (eligible designs, clinical trials, cohort studies and case series, are described but justification for including non-randomised designs is only implicit).<br><br>**Item 4:** Y (systematic search of PubMed and EMBASE from 1992–2022 with clearly reported terms and PRISMA flow diagram).	**Item 5:** Y (two reviewers independently screened titles/abstracts and full texts with arbitration by a third reviewer).<br><br>**Item 6:** Y (data abstraction performed independently by two reviewers using a predefined spreadsheet).	**Item 7:** N (reasons for exclusion are summarised in the PRISMA diagram but no complete study-level table of excluded reports with reasons is provided).<br><br>**Item 8:** Y (included studies are described in multiple tables detailing design, participants, interventions, outcomes and key conclusions).	**Item 9:** N (no formal, domain-based risk-of-bias or quality assessment tool is applied to individual primary studies; limitations are instead summarised narratively).<br><br>**Item 10:** N (funding sources and conflicts of interest for the primary studies are not systematically extracted or used in interpretation).	**Item 11:** NA (no meta-analysis undertaken; all results are synthesised narratively with descriptive tables).<br><br>**Item 12:** NA (not applicable in the absence of meta-analysis).	**Item 13:** Y (study-level limitations such as small samples, short duration and lack of controls are explicitly discussed and used to temper conclusions).<br><br>**Item 14:** NA (no statistical heterogeneity assessment required without pooled effect estimates).	**Item 15:** N (no formal or informal assessment of publication bias or small-study effects is reported).<br><br>**Item 16:** Y (review-level funding and authors' conflicts of interest are clearly stated).	Critically low – several critical weaknesses (items 2, 7, 9 and 15) despite appropriate searching, duplicate study selection and transparent tabular reporting of included studies.
Couper et al., 2025 [66]	**Item 1:** Y (clearly defined PICO on how specific antiseizure medications (ASMs) affect sexual hormones, sexual function and semen parameters in adult males with epilepsy).<br><br>**Item 2:** N (no prospectively registered or publicly available protocol; methods are described only in the final publication, with the internal protocol available on request).	**Item 3:** Y (eligible designs, clinical trials, cohort, case-control, cross-sectional, pre-post and case series,are justified to capture a broad evidence base on ASM–hormone/sexual outcomes).<br><br>**Item 4:** PY (systematic search of Embase, PubMed and MEDLINE with explicit strategy and PRISMA flow, but no trial-registry or grey-literature search and only three databases used).	**Item 5:** Y (two reviewers independently screened titles/abstracts and full texts with arbitration by a third reviewer).<br><br>**Item 6:** Y (study characteristics and outcome data were extracted by one reviewer and independently checked by a second, with disagreements resolved by discussion and third-reviewer input).	**Item 7:** PY (PRISMA diagram summarises numbers of full-text exclusions with reasons, but no separate table lists each excluded study with the specific reason).<br><br>**Item 8:** Y (included studies are described in detail in large summary tables covering design, ASM exposure, comparators, outcomes and main findings).	**Item 9:** Y (risk of bias for primary studies was assessed using NIH quality tools for observational and pre–post designs and the Cochrane RoB-2 tool for RCTs).<br><br>**Item 10:** N (funding sources and conflicts of interest of individual primary studies were not systematically extracted or synthesised).	**Item 11:** Y (random-effects meta-analyses with appropriate effect measures, mean differences and rate ratios, and heterogeneity statistics were applied to clinically coherent study groupings).<br><br>**Item 12:** PY (risk-of-bias assessments and study quality are discussed when interpreting pooled results, but there is no formal weighting or sensitivity analysis based explicitly on RoB levels).	**Item 13:** Y (limitations such as small samples, uncontrolled confounding and high between-study heterogeneity are explicitly considered when drawing conclusions and clinical implications).<br><br>**Item 14:** Y (statistical heterogeneity is quantified with I^2 and explored through stratified analyses by ASM type and outcome).	**Item 15:** PY (no formal funnel plots or tests for publication bias were feasible for most meta-analyses because of few studies, but the potential for small-study and reporting biases is acknowledged in the discussion).<br><br>**Item 16:** Y (review-level funding source and authors' conflicts of interest are clearly reported, with no relevant COI declared).	Low – one critical weakness (lack of a prospectively registered protocol) and several non-critical limitations (restricted search sources, incomplete reporting of excluded studies and primary-study funding), but otherwise robust methods including duplicate processes, structured RoB assessment and appropriate meta-analytic techniques.

Chochina et al., 2016 [67]	**Item 1:** Y (clearly framed PICO on the efficacy of intracavernous injections (ICIs) for ED in men with SCI and on potential prognostic factors of response).<br><br>**Item 2:** Y (a detailed protocol was developed and prospectively registered in PROSPERO (CRD42014009288), and the review states that the analysis followed this protocol).	**Item 3:** Y (eligible study designs, RCTs, non-randomised cohort studies and case series, are explicitly prespecified as appropriate to capture all available data on ICI response in SCI).<br><br>**Item 4:** Y (comprehensive multi-database search in PubMed-Medline, Embase, EBSCO, Web of Science and Cochrane Library with defined keywords and supplementary reference checking; restricted to English/French/Spanish).	**Item 5:** Y (eligibility assessment performed independently and in a blinded standardised manner by two reviewers, with disagreements resolved by consensus or a third reviewer).<br><br>**Item 6:** Y (data extraction undertaken independently by two reviewers using predefined forms, with consensus and third-reviewer arbitration for discrepancies).	**Item 7:** N (no full list of excluded full-text articles with individual reasons is provided; only aggregate exclusion numbers appear in the PRISMA flow diagram).<br><br>**Item 8:** Y (characteristics of included studies, including sample size, design, ICI drug/dose, response definition, and quality scores, are reported in detailed tables).	**Item 9:** Y (risk of bias/quality of primary studies assessed using the IHE checklist for case series and the STROBE checklist for other designs, with scores summarised by study).<br><br>**Item 10:** N (funding sources and conflicts of interest of the individual primary studies are not systematically extracted or discussed).	**Item 11:** Y (appropriate meta-analytic methods applied, pooling response proportions with fixed- or random-effects models based on heterogeneity (I^2 and Q statistics) and conducting subgroup analyses by drug type).<br><br>**Item 12:** PY (study quality is described and acknowledged as generally low to moderate, but RoB does not explicitly inform weighting, sensitivity analyses or subgroup interpretation beyond descriptive comments).	**Item 13:** Y (limitations related to non-randomised designs, heterogeneous outcome definitions and possible reporting bias are explicitly integrated into the discussion and conclusions).<br><br>**Item 14:** Y (clinical and statistical heterogeneity is quantified and discussed, and separate analyses are presented for different ICI agents).	**Item 15:** N (publication bias could not be investigated with funnel plots or formal tests because of substantial heterogeneity, and no alternative assessment of small-study effects is provided).<br><br>**Item 16:** Y (review-level funding/COI statements are clearly reported, indicating no relevant conflicts of interest).	Critically low – at least two critical weaknesses (lack of a full excluded-studies list and absence of any formal publication-bias assessment) together with incomplete reporting of primary-study funding, despite otherwise rigorous search, duplicate processes and appropriate meta-analytic methods.
Afferi et al., 2020 [68]	**Item 1:** Y (clearly framed PICO on performance and safety of all ED treatments in men with SCI).<br><br>**Item 2:** N (no prospectively registered or published review protocol is reported).	**Item 3:** PY (all human clinical studies on ED treatments in SCI were eligible, but the rationale for including multiple non-randomised designs alongside RCTs is not explicitly justified).<br><br>**Item 4:** PY (systematic search in two major databases, PubMed and EMBASE, with defined keywords and PRISMA flow, but no use of additional databases or grey literature and restriction to four languages).	**Item 5:** PY (study selection steps are described with PRISMA terminology, but the use of duplicate independent screening is not clearly stated).<br><br>**Item 6:** N (data extraction procedures and number of reviewers involved are not described in sufficient detail).	**Item 7:** N (no table listing excluded full-text articles with reasons, only aggregate numbers in the PRISMA diagram).<br><br>**Item 8:** Y (included studies are summarised in multiple detailed tables covering design, interventions, outcomes and performance/safety results).	**Item 9:** N (no formal risk-of-bias or methodological quality assessment of the individual primary studies is reported).<br><br>**Item 10:** N (funding sources and conflicts of interest of the primary studies are not systematically collected or discussed).	**Item 11:** NA (no quantitative meta-analysis is performed; results are synthesised narratively).<br><br>**Item 12:** NA (not applicable in the absence of a meta-analysis).	**Item 13:** N (while some general limitations of the evidence are mentioned, the absence of a structured risk-of-bias assessment means that study limitations are not explicitly integrated into the interpretation of results).<br><br>**Item 14:** NA (no statistical heterogeneity measures are available because no MA was conducted).	**Item 15:** N (no assessment of publication bias or small-study effects is attempted).<br><br>**Item 16:** Y (review-level conflict-of-interest statement is clearly reported, with authors declaring no relevant COI).	Critically low – multiple critical weaknesses, including lack of protocol registration, absence of a formal risk-of-bias assessment and publication-bias analysis, and no list of excluded studies, despite otherwise acceptable PRISMA-guided reporting and comprehensive descriptive tables.

Chehensse et al., 2013 [69]	**Item 1:** Y (clear PICO-style question on ejaculation capacity after SCI and spinal control, including implications for a putative spinal generator of ejaculation).<br><br>**Item 2:** N (no prospectively registered or publicly available review protocol is mentioned).	**Item 3:** PY (inclusion of observational cohorts and case series is appropriate given the physiological question and rarity of RCTs, but the choice of designs is not explicitly justified).<br><br>**Item 4:** Y (multi-database search of MEDLINE, EMBASE, EBSCOhost and the Cochrane Library with strategy and time frame reported).	**Item 5:** N (study selection procedures are described but there is no statement that titles/abstracts and full texts were screened independently by two reviewers).<br><br>**Item 6:** N (data extraction appears to have been performed by a single reviewer or team without explicit duplicate abstraction).	**Item 7:** N (no table of excluded full-text articles with reasons is provided; only aggregate numbers are shown in a PRISMA-style flow diagram).<br><br>**Item 8:** Y (included studies are described in detail across large tables summarising patient characteristics, lesion profiles, stimulation methods and ejaculation outcomes).	**Item 9:** N (no formal, domain-based risk-of-bias or methodological quality assessment of the observational studies and case series is reported).<br><br>**Item 10:** N (funding sources of primary studies are not systematically collected or incorporated in the synthesis).	**Item 11:** Y (meta-analyses of ejaculation rates by stimulation modality and lesion characteristics are performed using appropriate random- or fixed-effect models for proportions).<br><br>**Item 12:** N (because no study-level RoB assessment was undertaken, potential bias is not explicitly integrated into the quantitative synthesis).	**Item 13:** Y (important design and reporting limitations—heterogeneity in lesion classification, stimulation protocols and outcome ascertainment—are discussed and used to temper inferences about spinal control of ejaculation).<br><br>**Item 14:** Y (between-study heterogeneity is acknowledged and addressed through stratified meta-analyses and statistical comparisons of ejaculation rates across subgroups).	**Item 15:** N (no formal assessment of publication bias or small-study effects, such as funnel plots or statistical tests, is presented).<br><br>**Item 16:** Y (review-level funding and author support are transparently reported in a dedicated funding section).	Critically low – multiple critical weaknesses, including lack of protocol registration, duplicate selection/extraction , primary-study RoB assessment and publication-bias evaluation, despite otherwise comprehensive searches, detailed tables and appropriate meta-analytic methods.
Gao et al., 2024 [71]	**Item 1:** Y (clearly framed PICO on the impact of deep brain stimulation (DBS) on urogenital function, including lower urinary tract symptoms, sexual function and quality of life in adults with Parkinson's disease).<br><br>**Item 2:** Y (review protocol prospectively registered in PROSPERO: CRD42023476661 and cited in the Methods section).	**Item 3:** PY (eligible observational cohorts and self-controlled studies, plus one retrospective cohort, are defined and broadly appropriate for a surgical exposure, but the rationale for focusing on these designs is only briefly implied).<br><br>**Item 4:** Y (systematic multi-database search of PubMed, Embase, Cochrane Library, Web of Science and Scopus from database inception to 27 February 2024, with no language restrictions and example search strategies reported).	**Item 5:** Y (titles/abstracts and full texts screened independently by two reviewers with consensus procedures and third-author arbitration for disagreements).<br><br>**Item 6:** N (data extraction undertaken using a predesigned form, but independent duplicate abstraction is not explicitly reported).	**Item 7:** N (no detailed table of excluded full-text studies with individual reasons; only aggregated counts in a PRISMA-style flow diagram).<br><br>**Item 8:** Y (Table 1 and accompanying text provide adequate description of included studies, including country, sample size, stimulation site, outcomes and MINORS/NOS quality scores).	**Item 9:** Y (risk of bias for primary studies assessed using the Newcastle-Ottawa Scale for the cohort and MINORS for non-randomised studies, with scoring criteria and thresholds for high quality reported).<br><br>**Item 10:** N (funding sources and conflicts of interest for individual primary studies are not systematically extracted or incorporated into the synthesis).	**Item 11:** Y (RevMan-based meta-analyses using appropriate effect measures, mean differences and odds ratios, with I^2 statistics, sensitivity analyses and subgroup analyses for key outcomes).<br><br>**Item 12:** PY (study quality and methodological scores are discussed when interpreting findings, but RoB is not formally used to weight studies or conduct RoB-stratified sensitivity analyses).	**Item 13:** Y (interpretation explicitly acknowledges limitations of the evidence, including predominance of non-randomised designs, possible confounding and instability of some pooled estimates).<br><br>**Item 14:** Y (clinical and statistical heterogeneity explored using I^2, sensitivity analyses and subgroup analyses by stimulation site and dopaminergic medication control).	**Item 15:** Y (publication bias evaluated with Egger's tests and corresponding plots for several principal outcomes).<br><br>**Item 16:** Y (review-level funding and conflict-of-interest statements clearly indicate absence of external financial support and no COI).	Low – one critical weakness (absence of an excluded-studies table) and several non-critical limitations (unclear duplicate data extraction, partial design justification and incomplete reporting of primary-study funding), despite protocol registration, comprehensive searching, formal RoB assessment and appropriate meta-analytic methods.

Parittotokkaporn et al., 2020 [72]	**Item 1:** Y (clearly defined PICO on adults with SCI and neurogenic bowel, bladder and sexual (NBBS) dysfunction treated with non-invasive neuromodulation).<br><br>**Item 2:** N (no evidence of a prospectively registered protocol or PROSPERO/other registration).	**Item 3:** N (eligible designs broadly described as clinical human studies, but inclusion of RCTs, non-RCTs, case series and case reports is not explicitly justified).<br><br>**Item 4:** Y (comprehensive multi-database search of PubMed/Medline, EMBASE, Web of Science, Scopus and Cochrane with detailed keywords and PRISMA-style flow diagram plus reference list screening).	**Item 5:** N (study selection process summarised in a PRISMA flow diagram, but independent duplicate screening is not reported).<br><br>**Item 6:** Y (data extraction explicitly performed independently by two authors to minimise selection bias).	**Item 7:** N (no structured list of excluded full-text articles with individual reasons beyond aggregated counts in the flow diagram).<br><br>**Item 8:** Y (key characteristics of included studies—design, sample size, lesion characteristics, neuromodulation modality, stimulation parameters and NBBS outcomes—are tabulated in detail).	**Item 9:** Y (risk of bias of RCTs and non-RCTs assessed using RoB 2.0 and ROBINS-I, with results summarised in text and supplementary figures).<br><br>**Item 10:** N (funding sources and conflicts of interest for individual primary studies are not systematically extracted or incorporated into the appraisal).	**Item 11:** NA (no formal meta-analysis of treatment effects; results are synthesised narratively across heterogeneous modalities and outcomes).<br><br>**Item 12:** NA (not applicable in the absence of pooled quantitative estimates).	**Item 13:** Y (interpretation explicitly considers high risk of bias, small samples and confounding, and stresses the need for well-designed, sham-controlled RCTs before drawing firm efficacy conclusions).<br><br>**Item 14:** NA (no statistical heterogeneity metrics because no meta-analysis is presented; clinical heterogeneity is acknowledged narratively).	**Item 15:** N (no formal assessment of publication bias or small-study effects such as funnel plots or related tests).<br><br>**Item 16:** Y (review-level funding sources and authors' conflicts of interest are clearly reported).	Critically low – multiple critical weaknesses (items 2, 5, 7, 10 and 15) despite a comprehensive multi-database search and formal RoB assessment; findings should be interpreted with great caution at umbrella-review level.
Dunya et al., 2020 [73]	**Item 1:** Y (clearly formulated PICO on prevalence, symptomatology and management options of sexual dysfunction (SD) in adult women with multiple sclerosis (MS)).<br><br>**Item 2:** Y (review protocol prospectively registered with the International Prospective Register of Systematic Reviews (PROSPERO) and cited in the Methods section).	**Item 3:** PY (eligible observational and interventional designs, cross-sectional, case–control, follow-up and clinical trials, are described and appropriate to the objectives, but the justification for including all design types is only implicitly stated).<br><br>**Item 4:** Y (highly comprehensive multi-database search across 12 databases, including Cochrane, MEDLINE, EMBASE, CINAHL, AMED, PsycINFO, PEDro and rehabilitation/OT databases, with key terms, date limits and PRISMA flow reported).	**Item 5:** Y (titles/abstracts and full texts independently screened by two reviewers with consensus procedures and third-reviewer adjudication for disagreements).<br><br>**Item 6:** Y (data extraction undertaken in duplicate by two reviewers using a standardised form).	**Item 7:** N (no detailed table of excluded full-text articles with individual reasons; only aggregate numbers are shown in the PRISMA flow diagram).<br><br>**Item 8:** Y (observational and interventional studies are described in detail in several tables, including design, sample size, setting, questionnaires and main findings).	**Item 9:** Y (risk of bias/quality of quantitative studies assessed with the Effective Public Health Practice Project (EPHPP) tool, grading selection bias, design, confounders, blinding, data collection and withdrawals).<br><br>**Item 10:** N (funding sources and conflicts of interest of individual primary studies are not systematically extracted or incorporated into the synthesis).	**Item 11:** NA (no meta-analysis of prevalence or intervention effects; findings are summarised narratively and in tables).<br><br>**Item 12:** NA (not applicable because no statistical meta-analysis was undertaken).	**Item 13:** Y (interpretation explicitly reflects the generally weak methodological quality found on EPHPP assessment and emphasises limitations when drawing conclusions on prevalence and treatment evidence).<br><br>**Item 14:** NA (statistical heterogeneity is not evaluated since no pooled effect estimates are calculated).	**Item 15:** N (no formal or informal assessment of publication bias or small-study effects is reported).<br><br>**Item 16:** Y (review-level funding sources and potential conflicts of interest are clearly presented in the article).	Critically low – at least two critical weaknesses (lack of excluded-studies table and absence of publication-bias assessment) together with non-systematic reporting of primary-study funding, despite strengths in protocol registration, comprehensive searching, duplicate selection/extraction and formal quality appraisal.

Yavas et al., 2022 [70]	**Item 1:** Y (objective framed as a PICO question on PFMT for urinary incontinence and sexual function in adults with MS).<br><br>**Item 2:** Y (protocol prospectively registered in PROSPERO with prespecified eligibility criteria).	**Item 3:** Y (eligible study designs restricted a priori to randomised controlled trials and clinical trials, appropriate to the intervention-focused question).<br><br>**Item 4:** Y (comprehensive multi-database search across six databases with explicit strategy and PRISMA flow diagram).	**Item 5:** Y (two reviewers independently screened titles/abstracts and full texts with consensus procedures).<br><br>**Item 6:** Y (data extraction undertaken in duplicate using a standardised form).	**Item 7:** Y (supplementary table provides a list of excluded studies with reasons).<br><br>**Item 8:** Y (included trials described in detail, including MS characteristics, PFMT protocols, comparators and outcomes).	**Item 9:** Y (risk of bias of individual RCTs assessed with the revised Cochrane RoB 2.0 tool and summarised in plots).<br><br>**Item 10:** N (funding sources and COI of primary PFMT trials not systematically reported).	**Item 11:** NA (no quantitative MA conducted; authors appropriately refrained from pooling because of heterogeneity in interventions and outcome measures).<br><br>**Item 12:** NA.	**Item 13:** Y (interpretation of findings explicitly considers small sample sizes, RoB judgements and clinical heterogeneity).<br><br>**Item 14:** NA (no statistical heterogeneity analyses as no MA was performed).	**Item 15:** N (no formal assessment or detailed consideration of publication bias).<br><br>**Item 16:** Y (review funding statement and author COI disclosures reported).	Low – one critical weakness (item 15) and a non-critical limitation (item 10), otherwise methodologically robust with protocol registration, duplicate processes and structured RoB 2 assessment.

Legend: Population, Intervention, Comparator, Outcome (PICO); Risk of Bias (RoB); Meta-analysis (MA); Conflict of Interest (COI); AMSTAR 2 (A2); Central Nervous System (CNS); Sexual Dysfunction (SD); Yes (Y); Partially Yes (PY); Not Applicable (NA); Neurogenic Sexual Dysfunction (NSD); Preferred Reporting Items for Systematic Reviews and Meta-Analyses (PRISMA); Systematic Review (SR); Randomised Controlled Trial (RCT); Grading of Recommendations Assessment, Development and Evaluation (GRADE); People with Multiple Sclerosis (PwMS); Multiple Sclerosis (MS); Critical Appraisal Skills Programme (CASP); Phosphodiesterase Type 5 Inhibitors (PDE5Is); Spinal Cord Injury (SCI); Erectile Dysfunction (ED); International Prospective Register of Systematic Reviews (PROSPERO); MEDLINE Database (MEDLINE); EMBASE Database (EMBASE); Network Meta-analysis (NMA); PRISMA Extension for Network Meta-Analyses (PRISMA-NMA); Sexual Encounter Profile (SEP); Global Assessment Question (GAQ); Physical Therapy (PT); Cumulative Index to Nursing and Allied Health Literature (CINAHL); Strengthening the Reporting of Observational Studies in Epidemiology (STROBE); Subthalamic Nucleus (STN); Deep Brain Stimulation (DBS); Quality of Life (QoL); Risk Of Bias In Non-randomized Studies (ROBINS); Agency for Healthcare Research and Quality (AHRQ); Intracavernosal Injection (ICI); Intracavernosal Injections (ICIs); Effective Public Health Practice Project Quality Assessment Tool (EPHPP); Traumatic Brain Injury (TBI); Antiseizure Medications (ASMs); National Institutes of Health (NIH); EBSCOhost Database (EBSCO); Institute of Health Economics (IHE); Methodological Index for Non-Randomized Studies (MINORS); Newcastle–Ottawa Scale (NOS); Neurogenic Bowel, Bladder and Sexual (NBBS); Risk Of Bias In Non-randomized Studies of Interventions (ROBINS-I); Allied and Complementary Medicine Database (AMED); Occupational Therapy (OT); Pelvic Floor Muscle Training (PFMT); A Measurement Tool to Assess Systematic Reviews (AMSTAR).
