# Supplementary material for: Sexual Health After Neurological Disorders: A Comprehensive Umbrella Review of Treatment Evidence
Source: Med Sci (Basel). 2026 Jan 10;14(1):37. doi: 10.3390/medsci14010037 (PMC12821412; doi:10.3390/medsci14010037)
Supplement: Supplementary file 1 [file medsci-14-00037-s001.zip › Supplementary Table 2.pdf]

Supplementary Table 2. Summary of included systematic reviews on sexual health after neurological disorders.

Author, year / Country / Type of study	Aim of the review / Neurological condition	Population (age, sex, setting)	Intervention categories (type and main examples)	Comparators and primary study design	Sexual health outcomes and main findings	Number and types of included studies	Methodological notes / certainty of evidence
Lombardi et al., 2015 [48]<br><br>**Country:** Italy / Switzerland<br><br>**Type of study:** SR of interventions for SD due to CNS disorders (no MA)	**Aim of the review:** To systematically assess management strategies for SD secondary to CNS disorders, with emphasis on pharmacological, neuromodulatory and device-based treatments.<br><br>**Neurological condition:** Adults with SD due to CNS disease, including SCI, MS, PD, epilepsy, stroke and adult spina bifida.	**Age:** Adults (≥18 years) with established CNS disorder and SD; paediatric cohorts excluded.<br><br>**Sex:** Predominantly men with neurogenic ED after SCI; smaller samples of women with SCI, MS or epilepsy; aggregated M/F counts not reported.<br><br>**Setting:** Neuro-urology, neurology and rehabilitation services in single- and multicentre hospital-based trials.	Pharmacological: PDE5Is (sildenafil, tadalafil, vardenafil) as first-line therapy; additional agents including apomorphine, pergolide, testosterone±anastrozole, fampridine and lamotrigine. Device/neuromodulatory: sacral neuromodulation, perineal electrostimulation, vacuum erection devices, ICI with prostaglandin or Trimix, and penile prostheses.	**Comparators:** Placebo in most PDE5I and fampridine trials; active comparisons between PDE5Is or versus ICI/vacuum devices; usual care or uncontrolled pre–post designs for neuromodulation, hormonal and surgical series.<br><br>**Primary study design:** Mixed body of evidence comprising parallel and crossover RCTs, open-label prospective studies and small observational case series.	Sexual health outcomes were mainly assessed with IIEF-15/IIEF-5 and other validated male and female SF instruments. RCTs in men with SCI showed clinically important improvements in EF, intercourse success and sexual satisfaction with PDE5Is, with acceptable safety. Evidence for PDE5Is in MS, PD and epilepsy, and for female SD, was sparse and inconsistent. The SR concluded that robust evidence is essentially confined to PDE5Is for neurogenic ED in men with SCI, with major gaps for women and other CNS conditions.	**Total:** 31 primary studies.<br><br>**By design:** Majority RCTs evaluating PDE5Is in men with SCI; remaining studies are smaller non-randomised prospective trials, observational cohorts and comparative series; exact counts by design not consistently reported.	**Notes:** PRISMA-based SR with duplicate screening and data extraction; English-language restriction; no registered protocol, no formal RoB or GRADE assessment and no MA; overlap with later NSD SRs.<br><br>**Certainty of evidence:** Overall low to very low at umbrella-review level; confidence somewhat higher for PDE5Is in male SCI populations and very low for female SD and non-SCI CNS disorders.
Del Popolo et al., 2020 [49]<br><br>**Country:** Italy<br><br>**Type of study:** SR of NSD treatments (no MA)	**Aim of the review:** To summarise available treatments for NSD due to CNS disorders and peripheral neuropathies, stratified by neurological aetiology and sex.<br><br>**Neurological condition:** Adults with NSD arising from SCI, MS, PD, peripheral neuropathies and other neurogenic causes of ED or broader SD.	**Age:** Adult patients only; individual trials mainly include middle-aged men; paediatric studies excluded.<br><br>**Sex:** Evidence base dominated by men with neurogenic ED after SCI; relatively few small cohorts of women with MS or SCI.<br><br>**Setting:** Urology and neuro-urology departments, rehabilitation centres and hospital outpatient clinics.	First-line pharmacological therapy with PDE5Is (sildenafil, tadalafil, vardenafil). Second-line ICI with prostaglandin E1, papaverine or phentolamine and vacuum erection devices. Third-line surgical therapy with penile prosthesis implantation; limited additional data for neuromodulation and other agents.	**Comparators:** Placebo in most PDE5I RCTs; active comparisons between different PDE5Is or versus ICI/vacuum devices; usual-care or no-treatment controls in observational series and surgical cohorts.<br><br>**Primary study design:** Mixture of RCTs, retrospective and prospective observational studies and non-randomised comparative trials in adult NSD populations.	Sexual outcomes were chiefly measured with IIEF, SEP and related EF and satisfaction scales. Across RCTs in men with neurogenic ED, PDE5Is produced significant short-term improvements in EF and intercourse outcomes with good tolerability. ICI and vacuum devices were effective second-line options, especially in SCI, and penile prostheses restored penetrative capacity in severe or refractory cases despite higher infection risk. Evidence for treatment of female NSD was extremely limited and heterogeneous, preventing firm therapeutic recommendations.	**Total:** 46 original studies.<br><br>**By design:** Combination of RCTs of PDE5Is and other drugs, non-randomised prospective studies, retrospective cohorts and small case series on ICI, vacuum devices, neuromodulation and penile prostheses; precise counts by design category not tabulated.	**Notes:** PRISMA-compliant SR with PICOS-based selection, multi-database search to July 2019 and duplicate data extraction; non-English reports excluded; no MA or formal RoB/quality grading; substantial overlap with Lombardi 2015 for PDE5I trials and surgical series.<br><br>**Certainty of evidence:** Overall low certainty owing to heterogeneity, small samples and incomplete reporting; confidence higher for PDE5Is in male SCI but very low for peripheral neuropathies and female NSD.
Stratton et al., 2020 [50]<br><br>**Country:** Australia<br><br>**Type of study:** Cochrane SR of RCTs (no MA)	**Aim of the review:** To evaluate the effectiveness and safety of pharmacological and non-pharmacological interventions for SD after stroke.<br><br>**Neurological condition:** Adult stroke survivors with post-stroke SD, including premature ejaculation, ED and broader disturbances of sexual function and intimacy.	**Age:** Adults ≥18 years; individual RCTs enrolled younger men (23–45 years) with premature ejaculation and older mixed-age stroke cohorts.<br><br>**Sex:** Overall sample predominantly male; one RCT included only men with ED, another enrolled mixed-sex stroke survivors (and partners).<br><br>**Setting:** Inpatient stroke rehabilitation units and outpatient rehabilitation/physiotherapy services.	Interventions included sertraline for secondary premature ejaculation, an individualised structured sexual rehabilitation programme combining education and counselling, and targeted PFMT for post-stroke ED and LUTS.	**Comparators:** Sertraline versus methylcobalamin; sexual rehabilitation versus written educational material alone; PFMT versus standard general rehabilitation without specific LUTS/ED treatment.<br><br>**Primary study design:** Three small parallel-group RCTs with short- to medium-term follow-up (up to 6–12 months).	Sexual outcomes were measured with intravaginal ejaculatory latency time, non-validated sexual function ratings, IIEF scores and the Sexual Functioning Questionnaire Short Form. Sertraline modestly increased ejaculatory latency and some sexual function scores versus methylcobalamin but caused frequent mild gastrointestinal adverse effects. Neither structured sexual rehabilitation nor PFMT produced clear or durable improvements in sexual function, QoL or psychological outcomes compared with controls. The authors judged all findings as based on low or very low-quality evidence, insufficient to guide routine clinical practice.	**Total:** 3 RCTs (212 participants).<br><br>**By design:** One pharmacological RCT (sertraline vs methylcobalamin, n=114), one psycho-educational rehabilitation RCT (individualised sexual rehabilitation vs written information, n=68) and one physiotherapy RCT (PFMT vs standard rehabilitation, n=31).	**Notes:** Comprehensive multi-database search to November 2019; explicit inclusion of RCTs only; duplicate screening and data extraction; formal RoB assessment and GRADE 'best evidence' synthesis; substantial heterogeneity of interventions and outcomes precluded MA.<br><br>**Certainty of evidence:** Very low to low certainty across all comparisons due to small sample sizes, high or unclear RoB and imprecision; overall evidence judged insufficient to recommend any specific intervention for post-stroke SD.

Giannopapas et al., 2023 [51]<br><br>**Country:** Greece<br><br>**Type of study:** SR of therapeutic approaches for SD in MS (no MA)	**Aim of the review:** To identify pharmacological and non-pharmacological therapeutic options for managing SD in people with MS and to appraise the methodological quality of the underlying clinical studies.<br><br>**Neurological condition:** Adults with MS presenting with SD; interventional and non-interventional therapeutic studies between 2010 and 2022, excluding purely surgical protocols.	**Age:** Adult people with MS in early to mid-adulthood; individual trials typically report mean ages around 40–45 years; no paediatric cohorts.<br><br>**Sex:** Mixed-sex samples, with male-only ED trials and female-only cohorts defined by FSFI scores; aggregate M/F counts not pooled across studies.<br><br>**Setting:** Outpatient neurology, rehabilitation and urology clinics and community-based exercise programmes.	Pharmacological interventions included sildenafil and tadalafil for male ED and intradetrusor onabotulinumtoxinA injections for MS-related LUTS with secondary sexual outcomes. Non-pharmacological approaches comprised aquatic exercise, structured pelvic floor exercise programmes with and without mindfulness or electro muscular stimulation and electromyograph biofeedback, yoga-based interventions, and assistive devices such as a CVSD and external vibration devices.	**Comparators:** Placebo in most pharmacological RCTs; active comparison of different exercise doses or device types; usual care or no-treatment controls in several behavioural and exercise protocols.<br><br>**Primary study design:** Mixed evidence base including parallel-group RCTs, quasi-experimental trials and small uncontrolled pre–post studies ranked A–C using Marrie–Wolfson criteria.	Sexual outcomes were mainly evaluated using IIEF-15 and FSFI scores, supplemented by symptom scales and voiding diaries. Daily tadalafil and onabotulinumtoxinA improved erectile or female sexual function and LUTS in selected MS cohorts, whereas sildenafil produced limited benefit and was not recommended as routine therapy. Non-pharmacological interventions such as aquatic exercise, pelvic floor–based programmes and assistive devices yielded short-term gains in sexual function or satisfaction, but small samples and heterogeneous designs prevented robust comparative conclusions.	**Total:** 9 clinical studies.<br><br>**By design:** 2 pharmacological RCTs of PDE5Is, 1 interventional onabotulinumtoxinA study with controlled comparison, and 6 non-pharmacological protocols (including higher-quality RCTs of aquatic or pelvic floor exercise and several small non-randomised or pre–post studies of yoga, pelvic floor–mindfulness combinations and assistive devices).	**Notes:** Single-database search in PubMed limited to 2010–2022; duplicate screening and data extraction with informal Marrie–Wolfson grading; no formal RoB or GRADE assessment and no MA; considerable heterogeneity in interventions and outcomes.<br><br>**Certainty of evidence:** Overall very low at umbrella-review level because of small, heterogeneous samples, short follow-up and incomplete reporting; confidence is somewhat higher for onabotulinumtoxinA and tadalafil studies, and very low for non-pharmacological approaches and female-specific interventions.
Pöttgen et al., 2020 [52]<br><br>**Country:** Germany / international RIMS group.<br><br>**Type of study:** SR of psychobehavioural interventions for SD in MS (no MA).	**Aim of the review:** To identify psychological and psychobehavioural interventions used to treat SD in MS and to evaluate their effects on sexual function and related psychological outcomes.<br><br>**Neurological condition:** Adults with clinically definite MS reporting SD or reduced sexual satisfaction; several studies included patients together with their partners or spouses.	**Age:** Adult MS samples with mean ages between approximately 32 and 48 years and mean disease duration of 3.7–10.3 years where reported.<br><br>**Sex:** Predominantly women; some RCTs enrolled only women or couples, whereas others included mixed-sex samples; overall 295 participants across studies.<br><br>**Setting:** Outpatient neurology and rehabilitation clinics and community-based or telephone-delivered programmes in the United States and Iran.	Psychobehavioural interventions included structured psychosexual therapy and counselling, PLISSIT-model–based sexual counselling, a brief multidisciplinary behavioural consultation, telephone-delivered CBT targeting depression, and group mindfulness-based stress reduction and yoga programmes aimed at fatigue or QoL with secondary sexual outcomes.	**Comparators:** Control conditions comprised usual care, educational material on sexuality and intimacy in MS, supportive emotion-focused therapy or, in some studies, no formal control group.<br><br>**Primary study design:** Four small RCTs and two longitudinal pre–post observational studies with intervention durations of 4–16 weeks and one study including short-term follow-up.	Sexual outcomes were assessed using FSFI, the sexual function subscale of MSQOL-54, the Sexual Satisfaction Survey, the MS intimacy and sexuality questionnaire and marital adjustment measures. Across all six studies, psychobehavioural interventions produced statistically significant short-term improvements in sexual function or satisfaction and often in depression, fatigue or relationship indices. However, effect sizes were modest, samples were small and heterogeneous, and evidence of sustained benefit beyond a few months was limited.	**Total:** 6 intervention studies (295 participants).<br><br>**By design:** 4 RCTs (three directly targeting SD and one depression-focused CBT trial with secondary sexual outcomes) and 2 uncontrolled or quasi-experimental pre–post studies of individual or couple-based psychosexual therapy.	**Notes:** Multi-database search (PubMed, MEDLINE, CINAHL, PsycINFO) with hand-searching; inclusion restricted to English/German reports with ≥10 participants and pre–post measurement of sexual outcomes; study quality appraised using CASP tools; no MA due to heterogeneity in interventions and measures.<br><br>**Certainty of evidence:** Overall low certainty, with concerns about performance and detection bias, small samples, variable control conditions and short follow-up; convergent positive findings suggest psychobehavioural therapies are promising but not yet supported by robust confirmatory trials.
Xiao et al., 2012 [53]<br><br>**Country:** China.<br><br>**Type of study:** Cochrane SR with MA of RCTs on sildenafil for ED in MS	**Aim of the review:** To assess the efficacy and safety of sildenafil citrate for ED in male patients with MS.<br><br>**Neurological condition:** Adult men with clinically definite MS and persistent ED, irrespective of MS disease course.	**Age:** Men older than 18 years; individual trials reported mean ages around 40–41 years and mean MS duration of roughly 11 years.<br><br>**Sex:** All participants were male with MS-related ED.<br><br>**Setting:** Multicentre outpatient neurology and andrology services in Europe and Iran.	Pharmacological intervention with oral sildenafil, typically initiated at 50 mg and titrated between 25 and 100 mg once daily or as needed before sexual activity over 4–12 weeks, alongside usual MS care.	**Comparators:** Matching placebo tablets in both RCTs; no active comparators or non-pharmacological co-interventions.<br><br>**Primary study design:** Two parallel-group, double-blind, placebo-controlled RCTs with short-term blinded treatment phases followed by non-randomised open-label extensions not included in the MA.	Sexual outcomes included IIEF erectile function items, SEP questions 2–3 on penetration and intercourse completion, and the GAQ, with QoL scales in one trial. Pooled analyses showed higher proportions of successful intercourse and positive global response with sildenafil versus placebo, although confidence intervals were wide for some endpoints. Sildenafil was generally well tolerated, with increased mild adverse events such as headache, flushing and dyspepsia, and two serious vascular events reported across trials.	**Total:** 2 RCTs (420 men with MS-related ED).<br><br>**By design:** both randomised, double-blind, placebo-controlled parallel-group trials with flexible sildenafil dosing and intention-to-treat analyses for efficacy and safety outcomes.	**Notes:** Comprehensive multi-database search (CENTRAL, MEDLINE, EMBASE, CBM and trial registries) without language restriction; duplicate study selection, data extraction and RoB assessment using Cochrane criteria; fixed-effect MA performed for key binary outcomes.<br><br>**Certainty of evidence:** GRADE assessments indicated low to moderate certainty due to attrition bias, limited number of trials and short follow-up; overall, the review supports short-term efficacy of sildenafil for ED in MS but underscores the need for larger, longer-duration RCTs.

García-Perdomo et al., 2016 [55]<br><br>**Country:** Colombia / Spain<br><br>**Type of study:** SR and MA of PDE5Is for ED after traumatic SCI	**Aim of the review:** To determine the effectiveness of PDE5Is for the treatment of ED in men with traumatic SCI compared with placebo.<br><br>**Neurological condition:** Adult men with ED attributable to traumatic SCI, irrespective of lesion level or completeness.	**Age:** Men older than 18 years; individual RCTs mainly enrolled patients in early to mid-adulthood, with one study restricted to ages 44–55 years.<br><br>**Sex:** All participants were male with SCI-related ED.<br><br>**Setting:** Hospital-based urology and rehabilitation clinics in Europe and North America.	Oral PDE5Is (sildenafil, tadalafil, vardenafil) given on-demand or as flexible-dose regimens over 4–12 weeks, aimed at restoring penetrative EF in men with traumatic SCI.	**Comparators:** Matching placebo tablets in all included trials; no active comparators or adjunctive non-pharmacological interventions.<br><br>**Primary study design:** Six randomised, double-blind, placebo-controlled clinical trials pooled in a random-effects MA.	Sexual outcomes were primarily assessed using IIEF-based EF scores, GEA/GEQ items and SEP questions on penetration and intercourse completion. Pooled analyses showed a large improvement in EF with PDE5Is versus placebo (standardised mean difference ~0.7), indicating clinically important benefit despite high between-study heterogeneity. Adverse events, mainly headache, flushing and gastrointestinal discomfort, were more frequent with PDE5Is but generally mild and transient.	**Total:** 6 RCTs (963 men with SCI-related ED).<br><br>**By design:** all were parallel-group, double-blind, placebo-controlled trials; four evaluated sildenafil, one tadalafil and one vardenafil.	**Notes:** PROSPERO-registered SR with multi-database search (MEDLINE, CENTRAL, EMBASE and others) without language restriction; duplicate screening and data extraction; RoB assessed with Cochrane tool; random-effects MA with subgroup analyses by PDE5I type.<br><br>**Certainty of evidence:** At umbrella-review level, certainty is judged low to moderate: results are consistent and biologically plausible but based on a small number of short-term RCTs with substantial heterogeneity and some unclear RoB domains.
Jia et al., 2016 [54]<br><br>**Country:** China<br><br>**Type of study:** SR and MA of double-blind RCTs of PDE5Is for SCI-related ED	**Aim of the review:** To evaluate the efficacy and safety of PDE5Is for ED secondary to SCI using pooled estimates from high-quality RCTs.<br><br>**Neurological condition:** Adult men with SCI-related ED enrolled in randomised, double-blind, placebo-controlled trials of sildenafil, tadalafil or vardenafil.	**Age:** Adults with mean ages typically in the third to fifth decades; detailed age ranges varied across trials.<br><br>**Sex:** All participants were male with ED after SCI.<br><br>**Setting:** Specialist urology and rehabilitation centres in Europe and the Middle East.	Oral PDE5Is (sildenafil, tadalafil, vardenafil) titrated within licensed dose ranges and administered on-demand for several weeks, with efficacy and safety outcomes systematically pooled.	**Comparators:** Placebo in all six RCTs; no head-to-head comparisons between different PDE5Is within individual trials.<br><br>**Primary study design:** Six parallel-group, double-blind, placebo-controlled RCTs meeting predefined methodological quality criteria and graded A–C for RoB.	Sexual outcomes included GEQ responses on improvement in penile erections and SEP2/SEP3 diary items capturing successful penetration and intercourse completion. MA showed that PDE5Is markedly increased the odds of reporting improved erections (GEQ OR ~12) and higher rates of successful intercourse (relative risks ~1.8–2.7) compared with placebo. Headache, flushing and gastrointestinal discomfort were more common with PDE5Is but largely mild and self-limiting, supporting an overall favourable benefit–risk profile.	**Total:** 6 double-blind RCTs.<br><br>**By design:** four sildenafil vs placebo, one tadalafil vs placebo and one vardenafil vs placebo, all with short-term blinded treatment phases.	**Notes:** Comprehensive search of MEDLINE, EMBASE and Cochrane Library without language restriction; duplicate selection and extraction; Cochrane RoB assessment with most trials graded moderate quality; fixed- and random-effects models used according to heterogeneity.<br><br>**Certainty of evidence:** Low to moderate certainty: pooled effects are large and consistent but limited by small number of trials, short follow-up and some unclear allocation and reporting domains.
Tienforti et al., 2025 [56]<br><br>**Country:** Italy<br><br>**Type of study:** SR and NMA of RCTs comparing different PDE5Is for SCI-related ED	**Aim of the review:** To compare the efficacy of individual PDE5Is in men with SCI-related ED and rank treatments using NMA.<br><br>**Neurological condition:** Adult men with traumatic SCI of at least 6 months' duration and persistent ED, irrespective of lesion level.	**Age:** Adult men typically in early to mid-adulthood; mean ages around 35–45 years across trials.<br><br>**Sex:** All participants were male with SCI-related ED.<br><br>**Setting:** Multicentre urology and spinal units in Europe and North America.	On-demand or flexible-dose oral sildenafil, tadalafil or vardenafil regimens evaluated in blinded RCTs, with each PDE5I compared against placebo and occasionally against another PDE5I.	**Comparators:** Placebo and, in some trials, alternative PDE5Is, allowing construction of a comparison network across molecules.<br><br>**Primary study design:** Ten randomised, blinded RCTs included in pairwise MA and frequentist NMA; primary endpoint was patient-reported improvement in EF irrespective of instrument used.	Sexual outcomes were expressed as the proportion of men reporting improved EF from baseline, based on study-specific questionnaires or IIEF-derived thresholds. Class-effect MA showed that PDE5Is were about four times more effective than placebo in improving EF. NMA ranked tadalafil as the most effective agent (SUCRA ~81%), followed by vardenafil (~68%) and sildenafil (~49%), although confidence intervals overlapped and head-to-head data were limited.	**Total:** 10 RCTs (1,492 men with SCI-related ED).<br><br>**By design:** double-blind placebo-controlled parallel-group trials of sildenafil, tadalafil and/or vardenafil, some with multiple active arms.	**Notes:** PRISMA-compliant SR registered in PROSPERO; multi-database search (PubMed, Web of Science, Scopus, Cochrane Library); duplicate screening, Cochrane RoB assessment and NMA with P-scores/SUCRA ranking; transitivity and consistency formally examined.<br><br>**Certainty of evidence:** Moderate certainty that PDE5Is outperform placebo; relative ranking of agents is less certain owing to sparse indirect comparisons, short follow-up and moderate RoB in several trials.

Afshar et al., 2022 [57]<br><br>**Country:** Iran / USA<br><br>**Type of study:** SR and MA of interventions targeting sexual function or SD in MS	**Aim of the review:** To identify and synthesise interventional studies evaluating sexual function or SD in people with MS and determine the most effective intervention categories.<br><br>**Neurological condition:** Women and men with MS, either unselected regarding sexual status (sexual function studies) or with documented SD	**Age:** Adults with MS, mean ages ranging from late twenties to late forties across included trials.<br><br>**Sex:** Both sexes represented; overall, women predominated, particularly in psychoeducational trials, whereas medical interventions for SD primarily targeted men.<br><br>**Setting:** Neurology and rehabilitation centres in Iran and other countries, plus community-based educational and exercise programmes.	Interventions grouped as psychoeducational (counselling, CBT-based programmes, self-care and skills training), exercise and rehabilitation (aerobic, yoga, aquatic and resistance training), medical (MS therapies and supplements) and multi-type combinations of these modalities, applied either to improve sexual function or to treat defined SD.	**Comparators:** Controls included usual care, wait-list, educational leaflets, sham procedures or alternative doses/contents of the same programme.<br><br>**Primary study design:** Mixed body of 41 interventional studies (27 sexual function, 14 SD) including RCTs, quasi-experimental trials, clinical trials and cohort designs between 1990 and 2021.	Sexual outcomes were measured using MSQOL-54 sexual function subscales and FSFI scores. MA suggested that psychoeducational interventions produced significant improvements in sexual function compared with controls, whereas exercise and rehabilitation programmes showed little or no consistent effect. In SD studies, most interventions improved at least one sexual subscale, with medical treatments effective for male SD and psychoeducational interventions particularly beneficial for women.	**Total:** 41 interventional studies.<br><br>**By design:** 27 sexual function studies (15 RCTs, 9 quasi-experimental and 3 other designs) and 14 SD-focused studies spanning psychoeducational, exercise, medical and multi-type interventions.	**Notes:** PRISMA-guided SR with broad multi-database search (1990–2021); study quality graded on a custom 37-item checklist and RoB appraised using Cochrane domains; MA performed for subsets with homogeneous measures.<br><br>**Certainty of evidence:** Overall low certainty due to variable methodological quality, heterogeneity in interventions and outcomes and frequent lack of blinding; strongest evidence supports psychoeducational programmes, particularly for women, whereas evidence for exercise and pharmacological strategies remains limited.
Gopal et al., 2021 [58]<br><br>**Country:** USA<br><br>**Type of study:** SR and MA of PT interventions for SD in MS	**Aim of the review:** To evaluate the effectiveness of PT interventions in improving sexual function, sexual satisfaction and emotional well-being in individuals with MS and SD.<br><br>**Neurological condition:** Adults with MS reporting SD or dyspareunia/pain with intercourse in trials including PT-scope interventions.	**Age:** Adults with MS; mean ages and disease duration varied but most trials enrolled participants in mid-adulthood.<br><br>**Sex:** Both sexes included, with a predominance of women in PFMT and mindfulness studies; some device trials combined MS and SCI samples.<br><br>**Setting:** Outpatient neurology and PT clinics and community exercise settings in Europe, Iran and North America.	PT-range interventions including yoga and other physical activity programmes, PFMT with or without biofeedback and neuromuscular electrical stimulation, use of clitoral vacuum suction and vibratory devices, and mindfulness-based or combined PFMT–mindfulness protocols.	**Comparators:** Controls ranged from no intervention and usual care to alternative devices, sham stimulation or non-specific group activities.<br><br>**Primary study design:** Seven RCTs and one cohort study (level II evidence or higher) with pre–post patient-reported outcomes; PEDro and STROBE checklists used for quality appraisal.	Sexual outcomes were captured using MSQOL-54 sexual function, FSFI, marital satisfaction scales and Likert ratings. Pooled within-group effects showed large improvements in sexual function, moderate gains in sexual satisfaction and moderately large gains in emotional well-being after PT interventions, with significant between-group advantage for sexual satisfaction. PFMT and mindfulness-based approaches emerged as particularly effective components across studies.	**Total:** 8 studies.<br><br>**By design:** 7 RCTs (most with small sample sizes) and 1 cohort study evaluating PT-based interventions with pre–post and between-group comparisons.	**Notes:** PRISMA-based SR using PubMed, CINAHL and PEDro; inclusion required MS diagnosis, SD or pain with intercourse and PT-scope intervention; effect sizes calculated and pooled using fixed/random-effects models depending on heterogeneity.<br><br>**Certainty of evidence:** Low to moderate certainty: convergence of positive findings across heterogeneous but generally moderate- to high-quality trials supports a beneficial role of PT, although small samples and limited long-term follow-up temper confidence.
Auger et al., 2020 [60]<br><br>**Country:** Canada<br><br>**Type of study:** SR of allied health professional–delivered interventions for post-stroke sexual rehabilitation (no MA)	**Aim of the review:** To document and describe the best available evidence on interventions for post-stroke sexual rehabilitation that can be delivered by allied health professionals.<br><br>**Neurological condition:** Adults with stroke (≥50% of each study sample) receiving sexual rehabilitation during inpatient or outpatient neurorehabilitation.	**Age:** Adult stroke survivors, with mean ages in the included studies generally between 50 and 70 years.<br><br>**Sex:** Mixed-sex cohorts; some PFMT and couples-based interventions mainly involved men with female partners.<br><br>**Setting:** Inpatient and outpatient stroke rehabilitation units, rehabilitation hospitals and community programmes in high-income countries.	Structured PLISSIT-informed sexual rehabilitation sessions, individual sexual counselling, PFMT-based PT programmes, interdisciplinary sexual rehabilitation delivered by occupational therapists, PT and psychologists, scripted sexuality interviews and intensive couples retreats.	**Comparators:** comparators were usual stroke rehabilitation or written information only.<br><br>**Primary study design:** Evidence base comprised 2 RCTs, 1 non-randomised controlled trial, 1 cohort study, 1 pre–post single-group study, 1 case report and 2 implementation studies.	Sexual functioning and satisfaction were assessed using validated questionnaires and study-specific ratings. Small RCTs and controlled studies suggested that brief structured sexual rehabilitation and PFMT can improve sexual function, satisfaction and intimacy beyond usual rehabilitation. Interdisciplinary programmes, scripted interviews and retreats appeared to increase discussion of sexuality and perceived relationship closeness, but estimates were imprecise and follow-up short.	**Total:** 8 intervention studies.<br><br>**By design:** 2 RCTs, 1 non-RCT controlled trial, 1 cohort study, 1 uncontrolled pre–post intervention, 1 detailed case report and 2 implementation studies focused on service delivery.	**Notes:** PRISMA-conform SR with multi-database search (MEDLINE, Embase, PsycINFO, CINAHL, Web of Science, PEDro and OTseeker) and duplicate screening; interventions described using the TIDieR checklist and mapped to occupational therapy evidence-based categories; no formal MA or GRADE.<br><br>**Certainty of evidence:** Overall low certainty because of small, heterogeneous studies and limited blinding; confidence is somewhat higher for structured PLISSIT-based rehabilitation and PFMT than for retreats and scripted interview interventions.

Bahadori et al., 2024 [63]<br><br>**Country:** Iran / international cohorts<br><br>**Type of study:** SR and MA of observational studies of STN-DBS for SD in PD	**Aim of the review:** To evaluate the impact of subthalamic nucleus STN-DBS on SD and QoL in people with Parkinson's disease.<br><br>**Neurological condition:** Adults with idiopathic PD treated with STN-DBS for motor symptom control and assessed for sexual function and QoL pre- and post-operatively.	**Age:** Mean age across meta-analysed cohorts was about 62 years, with mean PD duration of approximately 10 years.<br><br>**Sex:** Mixed-sex samples with a predominance of men; sex-stratified sexual outcomes were rarely reported.<br><br>**Setting:** Specialist movement-disorder and neurosurgical centres performing STN-DBS and providing longitudinal follow-up.	Bilateral STN-DBS implanted for PD, in addition to optimised dopaminergic therapy; stimulation parameters and targets were broadly comparable across cohorts.	**Comparators and primary study design:** All primary studies were non-randomised longitudinal cohorts or case–control series using before–after comparisons of sexual function and QoL after STN-DBS; some included non-operated comparison groups but these were not pooled in MA.	SD scores derived from validated scales showed a small but statistically significant improvement after STN-DBS, while QoL showed a moderate-to-large gain. Heterogeneity for sexual outcomes was modest and there was no clear publication bias. Improvements likely reflected overall motor and non-motor benefit rather than a direct pro-sexual effect, and sex-specific responses remain uncertain.	**Total:** 10 observational studies in the SR; 6 cohorts (532 participants) contributed to MA of sexual function and QoL.<br><br>**By design:** prospective or retrospective cohorts and case–control series with pre–post DBS assessments.	**Notes:** PRISMA-based SR with five-database search to June 2024; protocol registered in PROSPERO; RoB assessed with ROBINS-I, showing mainly low to moderate risk with some concerns about confounding and missing data; random-effects MA conducted using SMDs.<br><br>**Certainty of evidence:** Low to moderate certainty that STN-DBS is associated with modest improvement in sexual function and clearer gains in QoL, limited by non-randomised designs and under-reporting of female-specific outcomes.
Brandão et al., 2025 [61]<br><br>**Country:** Portugal<br><br>**Type of study:** SR (no MA) of psychological interventions to improve sexual health after stroke	**Aim of the review:** To systematically review the efficacy of psychological interventions, including potential digital formats, designed to improve sexual health in adult stroke survivors.<br><br>**Neurological condition:** Adults (≥18 years) with ischaemic or haemorrhagic stroke, sometimes together with partners or spouses, enrolled in interventions targeting sexual or relational health.	**Age:** Stroke survivors in mid- to late adulthood; mean ages in included studies typically between 50 and 70 years.<br><br>**Sex:** Mixed-sex samples with lower representation of women; one study evaluated couples and assessed outcomes for both partners.<br><br>**Setting:** Hospital-based stroke rehabilitation, outpatient services and community settings in Australia, South Korea, the Netherlands and the USA.	Psychological and psychoeducational sexual rehabilitation, predominantly based on the PLISSIT model, including brief individual counselling, structured sexual and relational counselling and interdisciplinary sexual rehabilitation; all interventions were delivered face-to-face, with no digital interventions identified.	**Comparators and primary study design:** Two RCTs compared PLISSIT-based counselling plus written information with written information alone; other designs included a non-equivalent controlled pre–post study, a cross-sectional evaluation of counselling and a single case study of interdisciplinary sexual rehabilitation.	Outcomes included sexual function, sexual satisfaction, relational quality and psychological distress. Most interventions improved sexual knowledge, satisfaction and intimacy, and some increased sexual activity frequency; however, one RCT found little added benefit of a single brief counselling session beyond written information. Effects on anxiety, depression and long-term sexual adjustment were inconsistent and based on small samples.	**Total:** 5 primary psychological intervention studies.<br><br>**By design:** 2 RCTs, 1 quasi-experimental nonequivalent controlled pre–post study, 1 cross-sectional study and 1 case study.	**Notes:** PRISMA-guided SR registered in PROSPERO; literature search in four databases; single-reviewer screening with consultation from co-authors; RoB assessed using Cochrane RoB, ROBINS-I and JBI checklists, indicating substantial concerns about confounding and blinding.<br><br>**Certainty of evidence:** Overall low certainty; the evidence suggests that PLISSIT-based structured interventions are promising but requires larger, methodologically robust trials and exploration of digital delivery formats.
Dusenbury et al., 2017 [62]<br><br>**Country:** USA / Denmark<br><br>**Type of study:** SR (no MA) of quantitative studies on determinants of sexual function after stroke	**Aim of the review:** To examine determinants of sexual function and dysfunction in men and women after stroke and to evaluate the effectiveness of available sexual rehabilitation interventions.<br><br>**Neurological condition:** Adults (≥18 years) with any stroke subtype (ischaemic, intracerebral haemorrhage or subarachnoid haemorrhage) in hospital, outpatient or community settings.	**Age:** Mean age across studies was about 58–61 years, reflecting an older adult stroke population.<br><br>**Sex:** Participants were predominantly male (around 90% of 1883 individuals across studies); women were under-represented and mainly included in descriptive cohorts.<br><br>**Setting:** Hospital-based, outpatient and community stroke populations from Europe, North America, Asia and Africa.	A minority of studies evaluated sexual rehabilitation interventions such as structured education, counselling and position-adaptation advice; most studies were observational analyses of determinants including lesion characteristics, depression, disability and medication effects.	**Comparator:** Intervention studies compared sexual rehabilitation or counselling with usual care or attention control.<br><br>**Primary study design:** Nineteen quantitative studies were included: 13 descriptive cross-sectional or cohort studies, 3 case–control studies and 3 intervention/RCT designs.	SD was common after stroke, with prevalence estimates ranging from about 20% to 75% and frequent reductions in libido, erectile function and orgasm. Depression, higher disability and some lesion locations were associated with worse sexual outcomes. Limited intervention trials suggested that targeted sexual rehabilitation can improve sexual satisfaction, erectile function and coital frequency, but evidence is based on small, often weak-quality studies.	**Total:** 19 studies.<br><br>**By design:** 13 descriptive observational studies, 3 case–control studies and 3 intervention or RCT studies.	**Notes:** Systematic search of PubMed, MEDLINE, CINAHL, Cochrane and PsycINFO for 2000–2016; study quality assessed with the Effective Public Health Practice Project (EPHPP) tool, yielding five moderate-quality and fourteen weak studies, mainly due to design and blinding limitations.<br><br>**Certainty of evidence:** Overall low certainty for determinant–outcome associations and very low certainty for intervention effectiveness because of predominance of weak observational designs and scarce high-quality RCTs.

Esteve-Ríos et al., 2020 [59]<br><br>**Country:** Spain<br><br>**Type of study:** SR of clinical trials of interventions to improve sexuality in women with MS (no MA)	**Aim of the review:** To evaluate the effectiveness of interventions aimed at improving sexuality and reducing SD in women with multiple sclerosis.<br><br>**Neurological condition:** Adult women with MS of any subtype who reported SD and were enrolled in clinical trials of pharmacological or non-pharmacological interventions.	**Age:** Adult women, mostly in mid-life, with mean ages across trials typically between 30 and 50 years.<br><br>**Sex:** Women only (611 participants across all included studies).<br><br>**Setting:** Neurology, rehabilitation and specialist MS clinics in Europe, North America and the Middle East.	Six groups of interventions: sexual therapy based on PLISSIT-style counselling, pharmacological agents (including onabotulinumtoxinA), PFMT with or without electrostimulation, yoga programmes, mindfulness-based interventions and vaginal or clitoral stimulation devices.	**Comparators and primary study design:** Randomised or quasi-randomised clinical trials compared active interventions with placebo, usual care, waiting-list control or alternative active therapies, with pre-post assessment of sexual outcomes.	Primary outcomes were FSFI domains such as desire, arousal, lubrication, orgasm, satisfaction and pain. Sexual therapy, PFMT (alone or with electrostimulation), onabotulinumtoxinA and clitoral devices consistently improved several FSFI domains; evidence for yoga, mindfulness and some pharmacological regimens was more variable. Effects on pain and mood were favourable in a subset of trials, but broader QoL effects were inconsistently reported.	**Total:** 12 trials including 611 women.<br><br>**By design:** mixture of RCTs and quasi-randomised clinical trials, most with small sample sizes per arm.	**Notes:** Comprehensive multi-database and grey-literature search to October 2019 with duplicate screening; methodological quality assessed via the Jadad scale, with nine low-quality and three good-quality trials; heterogeneity in interventions and outcomes precluded MA.<br><br>**Certainty of evidence:** Overall low certainty, tending towards moderate for sexual therapy, PFMT with or without electrostimulation and onabotulinumtoxinA, where multiple trials reported consistent improvements in sexual function domains.
DeForge et al., 2006 [64]<br><br>**Country:** Canada (evidence-based practice centre, Ottawa).<br><br>**Type of study:** SR of male sexuality and ED after SCI with quantitative pooling of case-series data.	**Aim of the review:** To evaluate sexuality in men with SCI and summarise the effectiveness and safety of available erectile interventions.<br><br>**Neurological condition:** Adolescent and adult males with traumatic or non-traumatic SCI and ED of neurogenic origin.	**Age:** Male adolescents and adults; most cohorts included young to middle-aged men with chronic SCI.<br><br>**Sex:** All participants were male; studies focused on male sexuality and erections after SCI.<br><br>**Setting:** Rehabilitation hospitals, SCI speciality centres and outpatient urology/rehabilitation clinics in North America and Europe.	Behavioural therapy and perineal muscle training, topical agents, intraurethral alprostadil, ICIs with papaverine, phentolamine or PGE1, vacuum tumescence devices, penile prostheses and sacral stimulators, together with oral agents including sildenafil.	**Comparators and primary study design:** Broad inclusion of RCTs, cohort studies and non-comparative case series; many interventions were evaluated in uncontrolled single-arm cohorts, whereas sildenafil and some ICI regimens were assessed in comparative or randomised designs.	Sexual outcomes were primarily erections sufficient for intercourse and patient-reported satisfaction. Pooled case-series data suggested very high success rates for ICIs (≈90% response) and slightly lower but still favourable rates for sildenafil (≈80% response), with vacuum devices and penile implants also effective but more invasive. Short-term gains in erectile performance were clear across modalities, whereas long-term psychosocial adjustment and broader sexuality outcomes remained largely unstudied.	**Total:** 49 reports on male sexuality after SCI.<br><br>**By design:** mixture of behavioural, pharmacological and device intervention studies including RCTs, observational cohorts and multiple non-comparative case series; five penile implant series (363 men) contributed detailed outcome data.	**Notes:** Extensive search of six databases and conference proceedings (1966–2003) plus industry data; duplicate screening, Jadad scores for RCTs, Newcastle–Ottawa Scale for observational studies and a 19-item checklist for case series; random-effects pooling of response proportions for ICIs and sildenafil.<br><br>**Certainty of evidence:** Low at the umbrella-review level due to predominant case-series designs, heterogeneous outcome measures and limited long-term follow-up, although consistency of high response rates for ICIs and sildenafil supports at least low to moderate confidence in short-term erectile benefits.
McLoughlin et al., 2023 [65]<br><br>**Country:** USA (University of Pennsylvania) with international primary studies.<br><br>**Type of study:** SR of testosterone therapy in men with SCI or TBI (no MA).	**Aim of the review:** To synthesise evidence on testosterone replacement therapy for men with SCI or TBI, focusing on body composition, metabolic and functional outcomes relevant to rehabilitation.<br><br>**Neurological condition:** Adult men with chronic SCI or TBI and either testosterone deficiency or low–normal testosterone receiving exogenous testosterone.	**Age:** Adults aged ≥18 years; SCI cohorts mostly in early to middle adulthood, with chronic injuries of several years' duration.<br><br>**Sex:** Male-only populations by design, as the review targeted men with SCI or TBI.<br><br>**Setting:** Specialised rehabilitation and research centres in North America, with interventions delivered in inpatient or outpatient programmes.	Transdermal testosterone patches, gels and injectable preparations, often combined with resistance training or FES-based exercise programmes aimed at augmenting lean mass and metabolic health in men with SCI; a single small RCT evaluated testosterone gel in men with TBI.	**Comparators and primary study design:** Mostly small RCTs or controlled trials comparing testosterone plus exercise versus exercise alone, testosterone versus placebo, or testosterone versus no treatment; one non-randomised cohort contributed additional data on long-term replacement in testosterone-deficient men with SCI.	Primary outcomes were lean tissue mass, bone density, REE and cardiometabolic markers, but restoration of physiological testosterone levels is relevant to sexual function and libido. In SCI cohorts, testosterone combined with FES or resistance training improved muscle mass, strength and bone quality, whereas testosterone alone showed smaller effects. The single TBI trial suggested a non-significant trend towards functional gains with testosterone gel. Direct measures of sexual function were rarely reported, so implications for ED or sexual satisfaction remain inferential.	**Total:** 12 primary studies over 30 years.<br><br>**By design:** 11 SCI-focused trials and 1 TBI RCT, including randomised placebo-controlled trials, crossover studies and small prospective cohorts.	**Notes:** PRISMA-compliant search of PubMed and EMBASE (1992–2022) with duplicate screening and predefined PICOS; detailed extraction of body composition and functional outcomes; RoB not formally graded by GRADE but design limitations and small samples were highlighted.<br><br>**Certainty of evidence:** Low to moderate for improvements in body composition and metabolic surrogates in men with SCI receiving testosterone plus exercise, but very low for conclusions about sexual function or TBI owing to scarce and heterogeneous data.

Afferi et al., 2020 [68]<br><br>**Country:** Switzerland / international collaborative group.<br><br>**Type of study:** SR of treatment options for ED in men with SCI (no MA).	**Aim of the review:** To provide an overview of performance and safety of all available ED treatments in men with SCI and to identify knowledge gaps for emerging therapies.<br><br>**Neurological condition:** Adult men with ED secondary to traumatic or non-traumatic SCI, irrespective of lesion level or completeness.	**Age:** Adult men with SCI across a wide age range; many cohorts involved young men injured in early adulthood.<br><br>**Sex:** Male-only SCI populations with neurogenic ED; female sexuality and fertility were outside the review scope.<br><br>**Setting:** Neuro-urology units, SCI rehabilitation centres and urological outpatient clinics in Europe, North and South America.	First-line oral PDE5Is (sildenafil, tadalafil, vardenafil), ICIs with prostaglandin E1 and combination regimens, vacuum erection devices, penile prostheses and emerging modalities such as sacral neuromodulation, TENS and intraurethral suppositories.	**Comparators and primary study design:** Inclusion of RCTs, prospective and retrospective cohort studies and case series without time restriction; most PDE5I and ICI studies used pre–post designs or comparisons against placebo or alternative agents, whereas prosthesis and neuromodulation data were largely observational.	Across studies, PDE5Is and ICIs consistently achieved high rates of erections sufficient for intercourse and high patient and partner satisfaction with acceptable side-effect profiles. Vacuum devices and penile prostheses also restored penetrative capacity but were less preferred by patients and carried higher complication rates, particularly infections and mechanical failure for implants. Evidence for neuromodulation, TENS and intraurethral agents was sparse and inconclusive, precluding firm recommendations.	**Total:** 47 eligible studies.<br><br>**By design:** predominately observational series and small clinical trials evaluating PDE5Is, ICIs, vacuum devices, penile prostheses and other modalities in SCI-related ED.	**Notes:** Systematic PubMed/EMBASE search up to February 2020 following PRISMA guidance, including English, Italian, German and Spanish articles; all human studies with ≥10 SCI patients and ED were eligible; no quantitative MA or formal GRADE appraisal performed.<br><br>**Certainty of evidence:** Low to moderate certainty that PDE5Is and ICIs are effective and relatively safe first-line options, but very low certainty for prostheses, neuromodulation and intraurethral agents owing to uncontrolled designs and limited data.
Chochina et al., 2016 [67]<br><br>**Country:** France (Rennes University Hospital and collaborators).<br><br>**Type of study:** SR and MA of ICIs for ED in men with SCI.	**Aim of the review:** To quantify the efficacy of ICIs for ED in men with SCI and explore demographic or lesion-related predictors of treatment response.<br><br>**Neurological condition:** Adult men with paraplegia or quadriplegia due to SCI and neurogenic ED treated with ICIs.	**Age:** Adults with SCI of varying chronicity; detailed age distributions differed across cohorts.<br><br>**Sex:** Male-only samples with SCI-related ED.<br><br>**Setting:** Rehabilitation and urology departments in Europe and North America providing ICI-based ED therapy.	ICIs with papaverine alone, papaverine plus phentolamine (bi-mix), alprostadil and other vasoactive combinations titrated to response during supervised dose-adjustment sessions.	**Comparators and primary study design:** Prospective and retrospective cohorts, case series and a few RCTs in which men with SCI received ICI and response was documented; most studies lacked formal control groups, focusing instead on within-patient response rates.	The primary endpoint was erection or rigidity sufficient for sexual intercourse. Pooled across 23 studies (713 men), ICIs achieved an overall response rate of 88%, with higher success for papaverine–phentolamine (≈93%) and papaverine alone (≈91%) and somewhat lower rates with alprostadil (≈80%). Adverse events were generally mild, and meta-regression did not identify robust predictors of response by lesion characteristics or drug type.	**Total:** 23 studies involving 713 men.<br><br>**By design:** case series, observational cohorts and a small number of RCTs and non-randomised comparative studies reporting ICI response rates.	**Notes:** PRISMA-based SR with PROSPERO registration (CRD42014009288); multi-database search (Medline, Embase, EBSCO, Web of Science, Cochrane Library) restricted to English, French and Spanish; methodological quality assessed with IHE criteria for case series and STROBE for observational designs; random-effects MA of response proportions with subgroup analyses by drug type.<br><br>**Certainty of evidence:** Low to moderate for high short-term erectile response rates to ICI in men with SCI, tempered by reliance on non-randomised series, heterogeneous dosing regimens and limited data on long-term adherence and complications.
Couper et al., 2025 [66]<br><br>**Country:** Canada (Western University, London, Ontario).<br><br>**Type of study:** SR and MA of ASM effects on sexual hormones and functions in males with epilepsy.	**Aim of the review:** To synthesise evidence on how specific ASMs influence sex hormones, sperm parameters and sexual function in adult males with epilepsy, excluding valproic acid.<br><br>**Neurological condition:** Adult males with epilepsy treated with one or more ASMs (excluding valproic acid) compared with untreated men with epilepsy, healthy controls or pre-treatment baselines.	**Age:** Men aged ≥18 years with focal or generalised epilepsy; age distributions varied, but most cohorts were in young to middle adulthood.<br><br>**Sex:** Male-only samples by design; gender identity was not separately analysed.<br><br>**Setting:** Neurology and epilepsy clinics in Europe, Asia and North America, with hormonal and andrological assessments performed in research or clinical laboratories.	Treatment with individual ASMs (e.g. carbamazepine, phenytoin, oxcarbazepine, lamotrigine, levetiracetam and other second- and third-generation agents) grouped by molecule, generation and enzyme-inducing profile to explore associations with hormone levels and sexual function.	**Comparators and primary study design:** Observational cohort, case–control, cross-sectional and pre–post studies comparing men taking specific ASMs with untreated men with epilepsy, healthy controls or their own pre-treatment values; randomised trials were rare and evaluated short-term switches between ASMs.	Outcomes included serum sex hormones, ED and broader sexual dysfunction. Meta-analyses suggested that oxcarbazepine was associated with higher testosterone, LH and FSH compared with healthy controls, whereas lamotrigine and levetiracetam showed little or no difference from comparison groups. Across most analyses, sexual hormone profiles and ED rates differed little between treated and untreated men with epilepsy, underscoring the multifactorial contribution of epilepsy itself and psychiatric comorbidity to sexual dysfunction.	**Total:** 32 studies in the SR, of which 22 contributed to MA.<br><br>**By design:** mix of cross-sectional, cohort, pre–post and a few randomised trials evaluating hormonal and sexual outcomes in males receiving specific ASMs.	**Notes:** Systematic search of Embase, PubMed and MEDLINE (January 2024) with predefined inclusion criteria; study quality assessed using NIH tools for observational and pre–post designs and RoB 2 for trials; random-effects models used to pool mean differences or rate ratios, with high heterogeneity for several ASM–outcome combinations.<br><br>**Certainty of evidence:** Low certainty overall due to heterogeneous designs, small samples and potential confounding by epilepsy severity and comorbidities, though evidence does

							not support major adverse hormonal effects for lamotrigine or levetiracetam and suggests complex, drug-specific profiles for older enzyme-inducing agents.
Chehensse et al., 2013 [69]<br><br>**Country:** France; literature largely from European and North American SCI centres.<br><br>**Type of study:** SR and MA of ejaculation outcomes after SCI across different stimulation methods.	**Aim of the review:** To synthesise evidence on ejaculation rates in men with SCI stratified by lesion level and completeness, and to test the hypothesis of a spinal generator of ejaculation by comparing success rates of different elicitation techniques.<br><br>**Neurological condition:** Adult men with complete or incomplete traumatic SCI, predominantly at cervical or thoracic level, with chronic anejaculation evaluated in specialised neuro-urology or fertility settings.	**Age:** Adults of reproductive age (mostly 20–50 years); age distribution variably reported but cohorts were predominantly young to middle-aged men living with chronic SCI.<br><br>**Sex:** Male-only samples; gender identity and sexual orientation were not systematically reported.<br><br>**Setting:** Neuro-urology, fertility and rehabilitation clinics in high-income countries, usually tertiary centres with access to specialised andrological assessment.	Spontaneous ejaculation with masturbation or coitus, PVS, and pharmacological augmentation with AchE inhibitors administered before masturbation or PVS.	**Comparators and primary study design:** Primarily observational case series and non-randomised cohorts without formal control groups; analyses compared ejaculation rates across lesion strata, stimulation modalities and completeness of injury.	Primary outcomes were ejaculation success rates and semen quality parameters. The MA showed low ejaculation rates with masturbation alone, substantially higher success with PVS and the highest rates when PVS or masturbation were combined with AchE inhibitors, especially in men with lesions above T10. Findings support the presence of a spinal generator of ejaculation preserved in many men with suprasacral SCI.	**Total:** 45 studies (n≈3851 men with SCI).<br><br>**By design:** Most were retrospective or prospective case series; a minority were small comparative cohorts. No high-quality RCTs were identified.	**Notes:** Comprehensive search with transparent eligibility criteria and structured data extraction, but formal risk-of-bias tools for primary studies were not applied. Considerable clinical heterogeneity in lesion characteristics, stimulation protocols and outcome definitions limited comparability and pooling.<br><br>**Certainty of evidence:** Low to moderate certainty that PVS and pharmacological augmentation improve ejaculation rates in men with suprasacral SCI, downgraded mainly for non-randomised designs, indirectness and inconsistency across heterogeneous case series.
Dunya et al., 2020 [73]<br><br>**Country:** United Kingdom, Turkey and Japan; multi-country academic collaboration drawing on global MS literature.<br><br>**Type of study:** SR of prevalence, clinical features and management options for SD in women with MS.	**Aim of the review:** To summarise prevalence, symptom profiles, contributory factors and available pharmacological and non-pharmacological management strategies for SD in adult women with MS.<br><br>**Neurological condition:** Adult females with clinically defined MS of any subtype, with SD considered as a primary, secondary or tertiary consequence of the neurological condition.	**Age:** Adult women with MS across the reproductive and perimenopausal age range; most samples involved women in early-to-middle adulthood, with occasional inclusion of older age groups.<br><br>**Sex:** Female-only cohorts by design, with occasional mixed-sex studies from which female data were extractable; partner characteristics were rarely reported.<br><br>**Setting:** Neurology, urology and rehabilitation clinics, community cohorts and mixed outpatient samples from Europe, North America, Asia and the Middle East.	Non-pharmacological strategies such as PFMT, electrostimulation, yoga, mindfulness-based programmes, psychoeducation and PLISSIT-model counselling, alongside limited trials of pharmacological agents including PDE5-Is and intradetrusor onabotulinumtoxinA.	**Comparators and primary study design:** Predominantly observational cross-sectional and case–control studies evaluating prevalence and correlates of SD, with a small number of RCTs and uncontrolled intervention studies comparing active treatments with usual care, wait-list or baseline.	SD prevalence in women with MS ranged widely but was consistently higher than in healthy controls, with dominant problems in desire, arousal, lubrication and orgasm. Non-pharmacological interventions, particularly PFMT, structured psychoeducation and mindfulness or yoga-based programmes, yielded short-term improvements in multiple sexual domains, while evidence for pharmacological options was sparse and methodologically weak.	**Total:** 61 studies (33 observational, 14 case–control, 4 follow-up cohorts and 10 intervention trials).<br><br>**By design:** Interventional evidence comprised small RCTs and quasi-experimental studies with short follow-up.	**Notes:** Protocol registered in PROSPERO and PRISMA-compliant methods with extensive multi-database searches. Study quality was graded using the EPHPP tool, revealing frequent selection bias, confounding, inadequate blinding and heterogeneous outcome measures that precluded meta-analysis.<br><br>**Certainty of evidence:** Very low to low certainty for most management strategies due to small samples, non-randomised designs and heterogeneity; moderate certainty that SD is highly prevalent in women with MS and strongly associated with mood, disability and bladder dysfunction.

Gao et al., 2024 [71]<br><br>**Country:** China and international centres providing DBS for PD; literature largely from tertiary neurology and neurosurgery services.<br><br>**Type of study:** SR and MA of DBS effects on urogenital function, including sexual outcomes, in PD.	**Aim of the review:** To evaluate how subthalamic nucleus or globus pallidus internus DBS influences LUTS and sexual function in people with PD.<br><br>**Neurological condition:** Adults with idiopathic PD treated with DBS, with or without concomitant dopaminergic medications, compared with pre-operative status or non-surgical controls.	**Age:** Middle-aged and older adults, typically in their 50s–70s, with long-standing PD; age was similar between DBS and comparator groups when reported.<br><br>**Sex:** Mixed-sex cohorts with predominance of males; only a subset of studies reported sexual outcomes, usually in men using IIEF-based measures.<br><br>**Setting:** Specialised movement-disorder centres and neurosurgical units, with follow-up in outpatient neurology or rehabilitation clinics.	Bilateral DBS targeting the subthalamic nucleus or globus pallidus internus, with post-operative optimisation of stimulation and medication regimens.	**Comparators and primary study design:** Self-controlled before–after designs comparing pre- and post-DBS assessments, plus a small number of prospective cohorts and controlled studies versus medically treated PD patients.	Sexual function was assessed mainly with the IIEF and patient-reported items within urological questionnaires. Pooled analyses suggested that DBS did not consistently improve erectile function or global sexual satisfaction, despite modest benefits on LUTS and urinary QoL. Individual studies reported heterogeneous effects, with some patients experiencing de novo or worsened sexual difficulties after surgery.	**Total:** 14 studies in the SR of which a minority reported sexual outcomes<br><br>**By design:** design included prospective or retrospective self-controlled cohorts and small controlled studies, with sample sizes typically <100 participants per study.	**Notes:** Meta-analytic methods were applied for several urinary outcomes, but sexual function data were too sparse and heterogeneous for robust pooling. Risk-of-bias assessment highlighted limitations in blinding, outcome reporting and control of medication changes around DBS.<br><br>**Certainty of evidence:** Low certainty regarding the effect of DBS on sexual function in PD due to small, heterogeneous samples, inconsistent results and potential confounding by medication adjustments and disease progression.
Parittotokkaporn et al., 2020 [72]<br><br>**Country:** Thailand and international SCI literature; studies conducted mainly in high-income rehabilitation settings.<br><br>**Type of study:** SR of non-invasive neuromodulation for neurogenic bowel, bladder and sexual (NBBS) dysfunction after SCI.	**Aim of the review:** To review the efficacy and safety of non-invasive neuromodulation techniques for improving NBBS function in individuals with SCI.<br><br>**Neurological condition:** Adults with traumatic or non-traumatic SCI of varying levels and completeness, living with chronic neurogenic bowel, bladder and/or sexual dysfunction.	**Age:** Adults across a broad age range, generally young to middle-aged; paediatric cases were rare and usually excluded from primary analyses.<br><br>**Sex:** Predominantly male cohorts reflecting SCI epidemiology, although several studies included mixed-sex samples without sex-stratified reporting of sexual outcomes.<br><br>**Setting:** Inpatient and outpatient rehabilitation units and neuro-urology clinics, mostly in tertiary centres with expertise in neuromodulation.	Non-invasive neuromodulation modalities including TENS, tibial nerve stimulation, sacral or lumbar transcutaneous spinal cord stimulation and vibratory stimulation targeting genital or perineal afferents.	**Comparators and primary study design:** Heterogeneous designs comprising RCTs, non-randomised controlled studies, crossover trials and uncontrolled pre–post series, with comparators ranging from sham stimulation and usual care to alternative neuromodulation protocols.	Sexual outcomes, reported in a subset of studies, included erectile response, ejaculatory success, sexual satisfaction and composite NBBS scores. Across modalities, several small studies suggested short-term improvements in erectile rigidity or ejaculation with genital vibration or spinal stimulation, but data were inconsistent and often not powered for sexual endpoints. Benefits for bowel and bladder symptoms were more consistently reported than for sexual function.	**Total:** 46 studies<br><br>**By design:** (7 RCTs, 36 non-randomised or uncontrolled intervention studies and 3 case reports), of which only a minority explicitly evaluated sexual outcomes alongside NBBS measures.	**Notes:** Systematic multi-database search with predefined eligibility criteria, but risk-of-bias was generally high due to small samples, lack of blinding and incomplete reporting. Diverse stimulation parameters and outcome instruments limited cross-study comparisons and prevented formal meta-analysis for sexual endpoints.<br><br>**Certainty of evidence:** Very low certainty for the effect of non-invasive neuromodulation on sexual outcomes after SCI, although signals of benefit justify well-designed RCTs targeting sexual function as a primary endpoint.
		**Age:** Adult participants, mostly				**Total:** 7 controlled trials	

Yavaş et al., 2022 [70]<br><br>**Country:** Turkey; review of international evidence on PFMT-based interventions in adults with MS.<br><br>**Type of study:** SR of PFMT for urinary and sexual outcomes in people with MS.	**Aim of the review:** To evaluate the effectiveness of PFMT-based interventions, alone or combined with behavioural or mindfulness components, on urinary incontinence, OAB symptoms and sexual function in adults with MS.<br><br>**Neurological condition:** Adults with clinically definite MS and concomitant urinary incontinence or OAB, with or without coexisting SD, treated in neurological or rehabilitation settings.	women in early-to-middle adulthood; mean ages across trials ranged approximately from the mid-30s to mid-50s.<br><br>**Sex:** Samples were predominantly female, with only one trial including men; none of the studies reported gender-diverse participants or stratified outcomes by sex.<br><br>**Setting:** Outpatient rehabilitation, physiotherapy or uro-neurology clinics delivering supervised PFMT programmes with home-based continuation.	PFMT protocols delivered individually or in groups, sometimes combined with electromyographic biofeedback, electrical stimulation or mindfulness-based training, targeting pelvic floor strength, endurance and awareness.	**Comparators and primary study design:** Small RCTs and controlled clinical trials comparing PFMT-based interventions with usual care, sham stimulation, education-only control or alternative exercise regimens, with follow-up typically limited to 8–24 weeks.	Sexual outcomes, assessed mainly with the FSFI, were reported in a minority of trials and suggested that PFMT, especially when combined with mindfulness or electrostimulation, can improve desire, arousal, lubrication and satisfaction alongside reductions in urinary leakage. However, effect sizes were imprecise and durability beyond the intervention period remains uncertain.	**By design:** (most randomised) including approximately 248 participants with MS; five trials focused primarily on urinary outcomes and two reported sexual function measures.	**Notes:** Methodological quality was mixed, with TESTEX scores in the low-to-moderate range and frequent issues around allocation concealment, blinding and adherence reporting. Heterogeneity in PFMT protocols and concomitant interventions complicated cross-trial synthesis.<br><br>**Certainty of evidence:** Low certainty that PFMT-based programmes can improve sexual function in adults with MS, with more consistent moderate-certainty evidence for benefits on urinary symptoms and related QoL domains.

Legend: Systematic Review (SR); Sexual Dysfunction (SD); Central Nervous System (CNS); Phosphodiesterase Type 5 Inhibitors (PDE5Is); Spinal Cord Injury (SCI); Multiple Sclerosis (MS); Parkinson's Disease (PD); Erectile Dysfunction (ED); Intracavernosal Injection (ICI); Randomised Controlled Trial (RCT); International Index of Erectile Function (IIEF); Neurogenic Sexual Dysfunction (NSD); Sexual Encounter Profile (SEP); Pelvic Floor Muscle Training (PFMT); Lower Urinary Tract Symptoms (LUTS); Quality of Life (QoL); Risk of Bias (RoB); Female Sexual Function Index (FSFI); Cognitive Behavioural Therapy (CBT); Multiple Sclerosis Quality of Life-54 (MSQOL-54); Clitoral Vacuum Suction Device (CVSD); Vibration Device (VD); Global Assessment Question (GAQ); Global Efficacy Question (GEQ); Network Meta-analysis (NMA); Surface Under the Cumulative Ranking Curve (SUCRA); Physical Therapy (PT); Acetylcholinesterase (AchE); Effective Public Health Practice Project Quality Assessment Tool (EPHPP); Deep Brain Stimulation (DBS); Subthalamic Nucleus Deep Brain Stimulation (STN-DBS); Neurogenic Bowel, Bladder and Sexual Dysfunction (NBBS); Transcutaneous Electrical Nerve Stimulation (TENS); Overactive Bladder (OAB); Functional Electrical Stimulation (FES); Resting Energy Expenditure (REE); Traumatic Brain Injury (TBI); Luteinising Hormone (LH); Follicle-Stimulating Hormone (FSH); Anti-seizure Medications (ASMs); Rehabilitation in Multiple Sclerosis Network (RIMS); Template for Intervention Description and Replication (TIDieR); Joanna Briggs Institute (JBI); Critical Appraisal Skills Programme (CASP); International Prospective Register of Systematic Reviews (PROSPERO); Physiotherapy Evidence Database (PEDro); Occupational Therapy Seeker Database (OTseeker); National Institutes of Health (NIH); Strengthening the Reporting of Observational Studies in Epidemiology (STROBE); Institute of Health Economics (IHE); Tool for the Assessment of Study Quality and Reporting in Exercise (TESTEX); Newcastle-Ottawa Scale (NOS).
